# Evidence-Based Quality Improvement: a Scoping Review of the Literature

**DOI:** 10.1007/s11606-022-07602-5

**Published:** 2022-09-29

**Authors:** Susanne Hempel, Maria Bolshakova, Barbara J. Turner, Jennifer Dinalo, Danielle Rose, Aneesa Motala, Ning Fu, Chase G. Clemesha, Lisa Rubenstein, Susan Stockdale

**Affiliations:** 1 Southern California Evidence Review Center, University of Southern California, Los Angeles, CA USA; 2 Gehr Family Center for Health Systems Science and Innovation, University of Southern California, Los Angeles, CA USA; 3 Center for the Study of Healthcare Innovation, Implementation & Policy, VA Greater Los Angeles Healthcare System, Los Angeles, CA USA; 4 School of Economics, Shanghai University of Finance and Economics, Shanghai, China; 5 University of Southern California, Los Angeles, CA USA; 6 RAND Health, RAND Corporation, Santa Monica, CA USA

**Keywords:** quality improvement, evidence-based, scoping review

## Abstract

**Background:**

Quality improvement (QI) initiatives often reflect approaches based on anecdotal evidence, but it is unclear how initiatives can best incorporate scientific literature and methods into the QI process. Review of studies of QI initiatives that aim to systematically incorporate evidence review (termed evidence-based quality improvement (EBQI)) may provide a basis for further methodological development.

**Methods:**

In this scoping review (registration: https://osf.io/hr5bj) of EBQI, we searched the databases PubMed, CINAHL, and SCOPUS. The review addressed three central questions: How is EBQI defined? How is evidence used to inform evidence-informed QI initiatives? What is the effectiveness of EBQI?

**Results:**

We identified 211 publications meeting inclusion criteria. In total, 170 publications explicitly used the term “EBQI.” Published definitions emphasized relying on evidence throughout the QI process. We reviewed a subset of 67 evaluations of QI initiatives in primary care, including both studies that used the term “EBQI” with those that described an evidence-based initiative without using EBQI terminology. The most frequently reported EBQI components included use of evidence to identify previously tested effective QI interventions; engaging stakeholders; iterative intervention development; partnering with frontline clinicians; and data-driven evaluation of the QI intervention. Effectiveness estimates were positive but varied in size in ten studies that provided data on patient health outcomes.

**Conclusions:**

EBQI is a promising strategy for integrating relevant prior scientific findings and methods systematically in the QI process, from the initial developmental phase of the IQ initiative through to its evaluation. Future QI researchers and practitioners can use these findings as the basis for further development of QI initiatives.

**Supplementary Information:**

The online version contains supplementary material available at 10.1007/s11606-022-07602-5.

## BACKGROUND

Evidence-based quality improvement (EBQI) is one of a growing number of strategies used to enhance quality improvement (QI) initiative impacts in clinical practice. EBQI aims to integrate scientific evidence and methods into the QI process while maintaining focus on team-based innovation and problem-solving within real-world settings. Standard healthcare QI approaches focus powerfully on the need for measurement to determine innovation effects, and teams are advised to consult subject matter experts to strengthen their work.^1–5^ There currently is no standard approach, however, for integrating evidence from relevant pre-existing scientific literature into QI innovation and evaluation. Comprehensive review and critical appraisal of relevant research, for example, is not typically emphasized or conducted.^6^ In practice, QI teams often use anecdotal evidence alone to shape innovations, and low-validity methods to evaluate them.^7^ EBQI initiatives, as a subset of all QI initiatives, aim to systematically incorporate pre-existing scientific evidence and methods into the QI process as a core focus.

Given its foundation in applying best evidence and distinct focus on collaboration with the practice, EBQI is increasingly recognized as a valuable approach to structure implementation of advances in healthcare delivery.^7^ Among other factors, the rapid evolution of partnership improvement initiatives between healthcare organizations and researchers, and the increasing availability of embedded healthcare researchers within healthcare organizations have made EBQI more accessible and attractive to healthcare organizations.^3–5^

To date, core elements of EBQI have not been well documented, leaving a critical knowledge gap about components of EBQI and how it differs from other QI approaches. In addition, evidence of the effects of employing EBQI has yet to be synthesized. We found no prior systematic reviews of EBQI, and while individual studies have shown promising results^8^ to our knowledge, EBQI has not been evaluated in an evidence synthesis across studies.

This scoping review explores the EBQI literature. We document how EBQI is defined in publications and aimed to identify key components that characterize this methodology across studies. The review catalogues definitions and characteristics of EBQI as currently used in practice. Particular emphasis was on the definition, scope, and use of evidence, i.e., the core aspect of EBQI. We also examined evidence of effectiveness of EBQI. The scoping review was guided by these review questions^10^:

- Review question 1: How is EBQI defined?
- Review question 2: How is evidence used to inform evidence-informed quality improvement initiatives?
  - Review question 2a: How is evidence defined in these initiatives?
  - Review question 2b: What are the components of EBQI?
- Review question 3: What is the effectiveness of EBQI to promote uptake of evidence-based practices?

Our objective was to conduct a systematic search to identify the available knowledge, provide a clear description of the methodology, and inform further development of methods for incorporating research evidence into QI initiatives.

## METHODS

The scoping review followed a detailed review protocol. We followed the steps outlined by Arksey and Malloy: (1) determining the research question; (2) identifying relevant studies; (3) selecting studies; (4) charting the data; and (5) collating, summarizing, and reporting the results.^9^ In addition, we conducted a consultation exercise to inform and validate findings. The project was deemed exempt by our institutional Human Subject Committee. The protocol was registered in the Open Science Framework and is publicly available.^10^ The reporting follows PRISMA-ScR, a PRISMA adaptation for scoping reviews.^11,12^

### Search Strategy

The literature searches are documented in the supplemental digital content (SDC). First, a search using the exact terms (“evidence based quality improvement,” “evidence-based quality improvement,” or “EBQI”) was employed to identify publications published to March 2020 that explicitly refer to EBQI in the title, abstract, or keyword of the publication (i.e., the elements that are searchable in research databases). All retrieved publications that used the terminology were included.

Second, we used a broader search strategy aimed at identifying QI initiative evaluations that were not explicitly labeled as EBQI. We assumed that some authors may not use the term “EBQI” even when they have used an evidence-based QI strategy and describe a similar approach in the full-text publication. We applied a string of exclusion criteria to arrive at a manageable sample (see eligibility section), and given the large literature on QI interventions,^19^ we searched only for studies published between 2017 and 2020.

### Sources

We searched PubMed (biomedical literature), CINAHL (nursing and allied health profession literature), and SCOPUS (social sciences). We searched for EBQI publications without date restriction, other QI studies were limited to three years of QI publications as described below in more detail.

### Eligibility Criteria

Eligibility criteria were organized in a SPIOS (study design, participants, intervention, outcome, setting) framework; full details are shown in the SDC. Briefly, we applied the following:

- EBQI–labeled publications: All publications using EBQI terminology were included in the data abstraction.
  - Primary care effectiveness subsample: Among EBQI publications, we identified studies reporting effectiveness results for the evaluation of an EBQI initiative. Studies had to report on patient health, and we restricted to primary care to identify a more homogenous sample of research studies.
- EBQI–compatible studies: Empirical studies involving U.S. healthcare professionals, reporting on an evaluation of a QI initiative in primary care, and documenting evidence review as part of their methodology to select, design, or implement a QI intervention. Evidence review was defined as a literature review undertaken at the beginning of the project, documentation of locally generated data to determine the need for the intervention (practice-based evidence), and/or utilizing of authoritative sources such as evidence-based clinical practice guidelines. Two independent literature reviewers screened citations and full-text publications; discrepancies were resolved through discussion in the team. Reviewers first excluded all citations that did not indicate an empirical evaluation of a QI initiative. The remaining citations were screened as full-text publication, applying all eligibility criteria described in the SDC (e.g., U.S.-based).

### Data Abstraction and Synthesis

Data abstraction was tailored to the review questions. We used ten features in total to characterize the included studies (described in more detail in the SDC):

- *Evidence to identify target*: using evidence (data) to identify the target of the QI initiative
- *Iterative*: conducting an interactive process for selecting the QI intervention
- *Engagement of stakeholders*: reaching out within the organization to ensure a collaborative process
- *Evidence to identify intervention*: reviewing evidence (research literature or local data) to select effective QI interventions
- *QI facilitation*: use of facilitation of the QI process
- *Leadership involvement*: involving clinical operations leadership in the QI initiative
- *Priority setting with leadership*: setting priorities for the QI initiative together with clinical operations leadership
- *Frontline engagement*: engaging frontline personnel early in the QI initiative
- *Evidence to determine success*: using data to determine the success of the QI initiative
- *Analytic support*: using analytic support to help QI teams

The abstraction domains had been developed by the QI content expert team members drawing on practical and research expertise (SH, ST, BT). The information was collated in evidence tables and component tables allow a concise overview. Effectiveness outcomes were summarized in a random-effects meta-analysis.

### Expert Consultation

The preliminary scoping review results were presented to Dr. Lisa Rubenstein, a proponent and conceptual originator of EBQI. The formal consultation step aimed to ensure that the review addresses the right questions, identified all relevant literature, and synthesized the included material appropriately. Dr. Rubenstein was not involved in the planning of the review and assessed methods and results de novo. The consultation exercise resulted in one additional domain (priority setting with leadership) that was added to the data abstraction (see SDC).

## RESULTS

**Figure 1:**
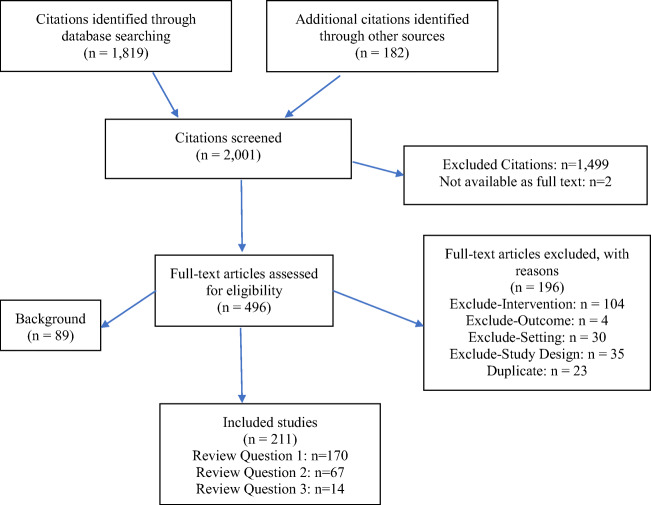
Flow diagram.

The literature searches identified 2001 citations. Of these, we obtained 496 for full-text inclusion screening. Figure 1 shows the flow diagram.

We included 211 publications, detailed in the evidence tables in the SDC. In total, we identified 170 diverse publications that used the term EBQI. SDC Figure 1 plots the number of EBQI publications over time and shows the rapid increase in frequency and popularity of EBQI. Two peaks emerged, one around 2006–2008, the other after 2016. The 170 identified publications are described in detail in an evidence table in the appendix (see SDC Table 1) and were used to address review question 1.

### Review Question 1 Synthesis: How Is EBQI Defined?

**Figure 2:**
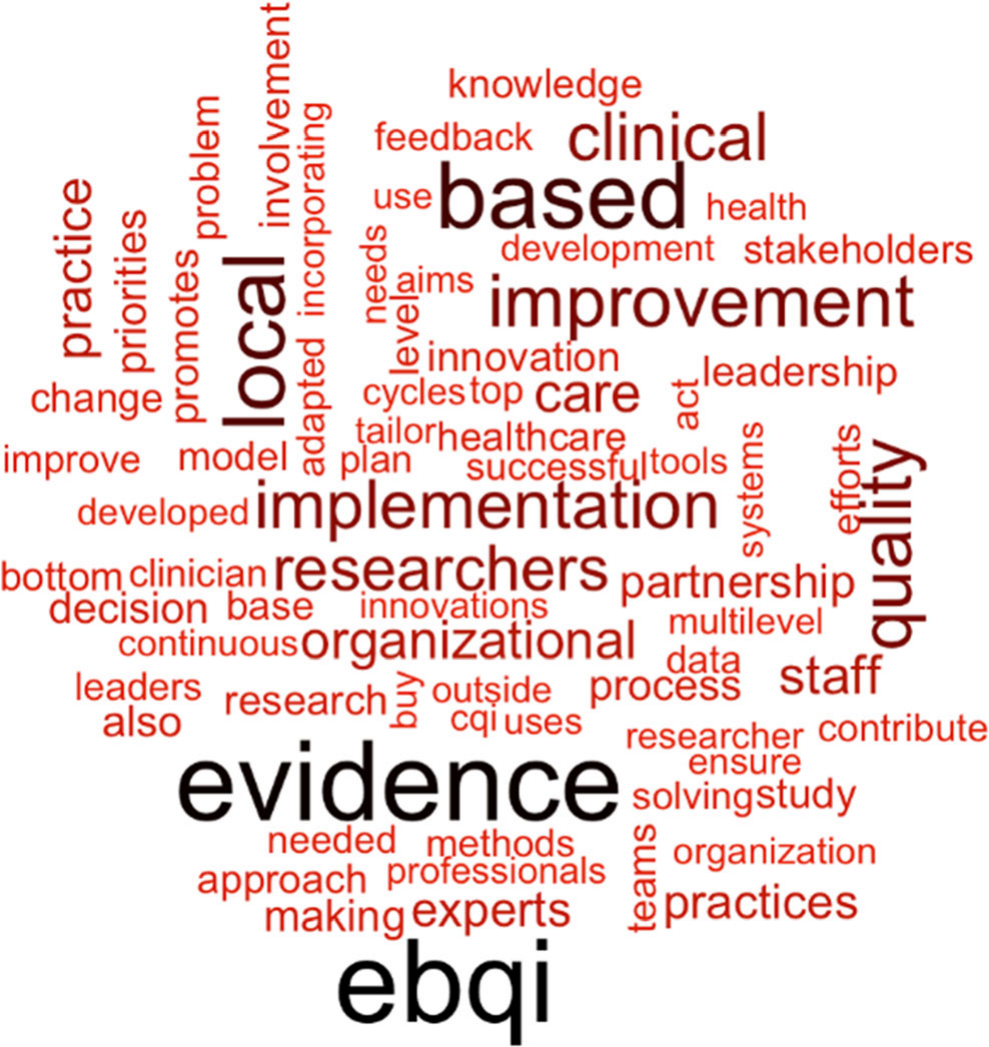
EBQI semantic definition overview.

The majority of EBQI–labeled publications did not define EBQI; only 23 of the 170 studies provided a definition or detailed description of the EBQI process. Studies highlighted different aspects of EBQI such as stakeholder engagement^13^ or described EBQI broadly as a continuous quality improvement method.^14^ Rubenstein et al.^15^ defined EBQI as “a continuous quality improvement approach whose goal is translation of research on care delivery models into routine practice.” Figure 2 shows the terms used in the identified publications.

### Review Question 2 Synthesis: How Is Evidence Used to Inform Evidence-Informed Quality Improvement Initiatives?

The second evidence table (SDC Table 2) shows all 25 EBQI–labeled studies that reported on an evaluation of a QI initiative (listed first), followed by the 42 EBQI–compatible primary care evaluations, for a total of 67 EBQI–labeled or EBQI–compatible studies. The table shows the wide range of clinical topic areas and interventions addressed and describes their implementation strategy in detail. Across studies, most used published research literature to select interventions to be implemented in the QI initiative.

### Review Question 2a Synthesis: How Is Evidence Defined?

In the 25 EBQI–labeled evaluations, 17 studies that provided information on the utilized evidence referred to published literature identified in a literature review. Ten EBQI studies referred to the use of local data. Six studies used expert panels and consensus meetings. Six studies referred to clinical practice guidelines that were reviewed to identify the QI intervention. Studies used these sources either alone or in combination.

### Review Question 2b Synthesis: What Are the Components of EBQI?

Table 1 shows the 10 potential EBQI features that we abstracted for each study, the number of features characterizing each study, and the overall frequency of features across studies. EBQI–labeled studies (top half of Table 1) are followed by EBQI–compatible studies (bottom half of Table 1). Table 2 provides a summary of features across all 67 studies. Across studies, two thirds of studies reported having used evidence to identify an effective intervention, engaging stakeholders, using an iterative development, and involving frontline clinicians. In addition, all 67 identified studies used data to determine the success of the QI initiative.

**Table 1:** Components in EBQI Studies and Components in EBQI–Compatible Studies

ID	Evidence to identify target	Iterative	Stakeholder engagement	Evidence to identify intervention	QI facilitation	Leadership involvement	Priority setting with leadership	Frontline engagement	Evidence to determine success	Analytic support	**Total criteria met within study (*N*** **= 10)** **(%)**
EBQI studies											
Badru, 2017^20^	Yes	Yes	Yes	Yes	No	No	No	Yes	Yes	Yes	**7** **(70%)**
Bennett, 2016^21^	Yes	Yes	Yes	Yes	No	Yes	No	Yes	Yes	No	**7** **(70%)**
Chaney, 2011^14^	No	Yes	Yes	Yes	No	Yes	Yes	Yes	Yes	Yes	**8** **(80%)**
Cohen, 2013^22^	Yes	Yes	Yes	No	Yes	No	No	Yes	Yes	No	**6** **(60%)**
Dumphy, 2016^16^	Yes	No	No	Yes	No	No	No	No	Yes	No	**3** **(30%)**
Fortney, 2012^23^	Yes	Yes	Yes	Yes	Yes	Yes	Yes	Yes	Yes	Yes	**10** **(100%)**
Fortney, 2013^24^	No	Yes	Yes	Yes	Yes	Yes	Yes	Yes	Yes	Yes	**9** **(90%)**
Fox, 2016^25^	No	Yes	Yes	Yes	Yes	Yes	Yes	Yes	Yes	Yes	**9** **(90%)**
Gadbois, 2016^26^	Yes	Yes	Yes	Yes	Yes	Yes	No	Yes	Yes	No	**8** **(80%)**
Gottlieb, 2018^17^	Yes	Yes	No	Yes	No	Yes	Yes	No	Yes	No	**6** **(60%)**
Klause, 2020^27^	Yes	Yes	No	Yes	No	No	No	Yes	Yes	No	**5** **(50%)**
Le Flore, 2017^28^	Yes	Yes	Yes	Yes	Yes	Yes	No	No	Yes	No	**7** **(70%)**
Meredith, 2018^29^	No	Yes	Yes	Yes	Yes	Yes	Yes	Yes	Yes	Yes	**9** **(90%)**
Ong, 2017^30^	Yes	Yes	Yes	No	No	No	No	Yes	Yes	No	**5** **(50%)**
Rizzo, 2018^31^	Yes	No	Yes	Yes	No	No	No	Yes	Yes	No	**5** **(50%)**
Rubenstein, 2006^32^	No	Yes	Yes	Yes	Yes	Yes	Yes	Yes	Yes	Yes	**9** **(90%)**
Rubenstein, 2010^15^	Yes	Yes	Yes	Yes	Yes	Yes	Yes	Yes	Yes	Yes	**10** **(100%)**
Sherman, 2004^33^	Yes	Yes	Yes	Yes	No	Yes	Yes	Yes	Yes	No	**8** **(80%)**
Starkey, 2016^34^	Yes	Yes	Yes	Yes	Yes	No	No	Yes	Yes	No	**8** **(80%)**
Walker, 2019^35^	No	No	Yes	Yes	No	No	No	Yes	Yes	No	**5** **(50%)**
Walker-Smith, 2020^36^	Yes	Yes	Yes	Yes	No	No	No	Yes	Yes	No	**7** **(70%)**
Whitten, 2013^37^	Yes	No	No	Yes	No	No	No	No	Yes	No	**3** **(30%)**
Yano, 2008^13^	No	Yes	Yes	Yes	Yes	Yes	Yes	Yes	Yes	No	**8** **(80%)**
Yoon, 2016^38^	Yes	Yes	Yes	Yes	Yes	Yes	Yes	Yes	Yes	Yes	**10** **(100%)**
Young, 2018^39^	Yes	No	No	Yes	No	No	No	No	Yes	No	**3** **(30%)**
**Total studies meeting EBQI criteria (*N*** **= 25)** ***N*** **(%)**	**18** **(72%)**	**20** **(80%)**	**20** **(80%)**	**23** **(92%)**	**12** **(48%)**	**14** **(56%)**	**11** **(44%)**	**20** **(80%)**	**25** **(100%)**	**9** **(36%)**	**N/A**
EBQI–compatible studies											
Barclay, 2019^40^	No	Yes	Yes	Yes	Yes	Yes	No	Yes	Yes	No	**7** **(70%)**
Bowen, 2020^41^	No	Yes	No	Yes	Yes	No	No	No	Yes	No	**4** **(40%)**
Breaux, Shropshire, 2017^42^	Yes	Yes	Yes	Yes	Yes	No	No	Yes	Yes	No	**7** **(70%)**
Brodie, 2018^43^	Yes	Yes	Yes	Yes	Yes	Yes	No	Yes	Yes	Yes	**9** **(90%)**
Burge, 2019^44^	Yes	Yes	Yes	No	No	Yes	No	Yes	Yes	No	**5** **(50%)**
Buschkoetter, 2019^45^	No	Yes	No	Yes	No	No	No	No	Yes	No	**3** **(30%)**
Camp, 2017^46^	Yes	No	No	Yes	No	No	No	No	Yes	No	**3** **(30%)**
Campbell, 2017^47^	No	Yes	No	No	No	No	No	No	Yes	No	**2** **(20%)**
Colborn, 2019^48^	No	Yes	Yes	Yes	No	No	No	No	Yes	No	**4** **(40%)**
Daaleman, 2018^49^	No	Yes	Yes	No	Yes	Yes	No	No	Yes	No	**5** **(50%)**
Fabre, 2020^50^	No	No	No	Yes	Yes	Yes	No	No	Yes	No	**4** **(40%)**
Fisher-Borne, 2018^51^	No	No	Yes	Yes	No	No	No	Yes	Yes	No	**4** **(40%)**
Fortney, 2018^52^	Yes	Yes	Yes	Yes	Yes	Yes	Yes	Yes	Yes	Yes	**10** **(100%)**
Garza, 2017^53^	No	Yes	No	Yes	No	No	No	No	Yes	No	**3** **(30%)**
Gold, 2017^54^	Yes	Yes	Yes	Yes	No	Yes	No	Yes	Yes	Yes	**8** **(80%)**
Green, 2017^55^	Yes	Yes	Yes	Yes	No	No	Yes	Yes	Yes	Yes	**9** **(90%)**
Hanlin, 2018^56^	No	Yes	Yes	Yes	No	No	No	No	Yes	No	**4** **(40%)**
Hawk, 2017^57^	No	No	Yes	Yes	No	No	No	Yes	Yes	No	**4** **(40%)**
Jonas, 2017^58^	No	Yes	No	Yes	No	No	No	No	Yes	No	**3** **(30%)**
Knierim, 2019^59^	No	No	No	Yes	Yes	No	No	No	Yes	Yes	**4** **(40%)**
Lu, 2019^60^	Yes	No	Yes	No	No	No	No	Yes	Yes	No	**4** **(40%)**
Makelarski, 2019^61^	No	No	Yes	Yes	Yes	Yes	No	Yes	Yes	No	**6** **(60%)**
Minsky, 2017^62^	Yes	No	Yes	No	No	No	No	Yes	Yes	No	**4** **(40%)**
Modica, 2019^63^	No	Yes	Yes	Yes	Yes	Yes	Yes	Yes	Yes	Yes	**9** **(90%)**
Nagykaldi, 2017^64^	No	No	Yes	Yes	Yes	No	Yes	No	Yes	Yes	**6** **(60%)**
Nowalk, 2017^65^	No	No	Yes	Yes	No	No	No	Yes	Yes	No	**4** **(40%)**
Ober, 2017^66^	No	Yes	Yes	Yes	Yes	Yes	No	No	Yes	No	**6** **(60%)**
Quanbeck, 2018^67^	No	Yes	Yes	Yes	Yes	Yes	No	Yes	Yes	Yes	**8** **(80%)**
Regan, 2017^68^	Yes	No	No	Yes	Yes	No	No	No	Yes	Yes	**5** **(50%)**
Richards, 2019^69^	No	Yes	Yes	Yes	Yes	Yes	No	Yes	Yes	No	**7** **(70%)**
Roderick, 2017^70^	No	Yes	Yes	Yes	Yes	No	No	No	Yes	Yes	**6** **(60%)**
Savas, 2019^71^	Yes	Yes	Yes	Yes	Yes	No	Yes	Yes	Yes	No	**8** **(80%)**
Schaeffer, 2019^72^	Yes	Yes	Yes	Yes	No	Yes	Yes	Yes	Yes	No	**8** **(80%)**
Schiff, 2017^73^	Yes	Yes	Yes	No	Yes	Yes	No	No	Yes	No	**6** **(60%)**
Schurman, 2017^74^	Yes	Yes	Yes	Yes	Yes	No	No	Yes	Yes	No	**7** **(70%)**
Senger, 2018^75^	Yes	No	Yes	Yes	No	Yes	Yes	Yes	Yes	No	**7** **(70%)**
Shah, 2019^76^	Yes	Yes	Yes	Yes	Yes	Yes	Yes	Yes	Yes	Yes	**10** **(100%)**
Sloand, 2019^77^	No	Yes	No	Yes	No	Yes	No	Yes	Yes	No	**5** **(50%)**
van Eeghen, 2020^78^	Yes	Yes	Yes	Yes	Yes	No	No	Yes	Yes	No	**7** **(70%)**
Weiner, 2017^79^	No	No	Yes	Yes	Yes	No	Yes	No	Yes	No	**5** **(50%)**
Williams, 2018 ^80^	Yes	Yes	Yes	Yes	Yes	No	No	Yes	Yes	No	**7** **(70%)**
Yusupov, 2019^81^	Yes	Yes	No	Yes	No	No	No	No	Yes	No	**4** **(40%)**
**Total studies meeting EBQI–compatible criteria (*N*** **= 42)** ***N*** **(%)**	**18** **(43%)**	**29** **(69%)**	**31** **(74%)**	**36** **(86%)**	**23** **(55%)**	**17** **(41%)**	**9** **(21%)**	**24** **(57%)**	**42** **(100%)**	**11** **(26%)**	**N/A**

Note: Evidence to identify target: using data to identify the target of the QI intervention; Iterative: iterative and interactive process for selecting the intervention within the discussion; Engagement of stakeholders: reaching out to stakeholders within the organization in a collaborative process; Evidence to identify intervention: literature review to identify effective interventions in the research literature; QI facilitation: quality improvement facilitation may refer to an external facilitator, internal QI coordinator, or learning collaborative; Leadership involvement: involvement of organizational leadership beyond one-time approval or briefing at the end; Priority setting with leadership: organizational leadership was involved in prioritizing the target of the QI intervention; Frontline engagement: engagement of frontline personnel from the start, not only after the process change was decided; Evidence to determine success: use of evidence to determine the effect of the intervention; Analytic support: support from a dedicated statistician or analyst beyond of the clinical team

**Table 2:** Summary of Components Across Studies

Components	All studies meeting criteria (*N* = 67) *N* (%)	EBQI studies (*N* = 25) *N* (%)	EBQI–compatible studies (*N* = 42) *N* (%)
Evidence to identify target	36 (54%)	18 (72%)	18 (43%)
Iterative development	49 (73%)	20 (80%)	29 (69%)
Engagement of stakeholders	51 (76%)	20 (80%)	31 (74%)
Evidence to identify intervention	59 (88%)	23 (92%)	36 (86%)
QI facilitation	35 (52%)	12 (48%)	23 (55%)
Leadership involvement	31 (46%)	14 (56%)	17 (41%)
Priority setting with leadership	19 (28%	11 (44%)	9 (21%)
Frontline engagement	44 (66%)	20 (80%)	24 (57%)
Evidence to determine success	67 (100%)	25 (100%)	42 (100%)
Analytical support	20 (30%)	9 (36%)	11 (26%)

Note: Evidence to identify target: using data to identify the target of the QI intervention; Iterative: iterative and interactive process for selecting the intervention within the discussion; Engagement of stakeholders: reaching out to stakeholders within the organization in a collaborative process; Evidence to identify intervention: literature review to identify effective interventions in the research literature; QI facilitation: quality improvement facilitation may refer to an external facilitator, internal QI coordinator, or learning collaborative; Leadership involvement: involvement of organizational leadership beyond one-time approval or briefing at the end; Priority setting with leadership: organizational leadership was involved in prioritizing the target of the QI intervention; Frontline engagement: engagement of frontline personnel from the start, not only after the process change was decided; Evidence to determine success: use of evidence to determine the effect of the intervention; Analytic support: support from a dedicated statistician or analyst beyond the clinical team

When we compared features across the subsets, evidence to identify the target of the quality improvement intervention was more frequently reported in EBQI–labeled studies than in EBQI–compatible studies (72% vs 43%). Across EBQI–labeled and EBQI–compatible studies, involvement of leadership in priority setting for the quality improvement target (44% vs 21%) and the provision of analytic support (36% vs 26%) were least frequently reported. EBQI studies consistently reported more EBQI features: the median number of components used within study was 7 for the EBQI–labeled sample (maximum of 10) and 5.5 for the EBQI–compatible sample. The distributions in the two sets differed statistically significantly (*p* = 0.037; Mann-Whitney *U* test).

### Review Question 3 Synthesis: EBQI Effectiveness

**Figure 3:**
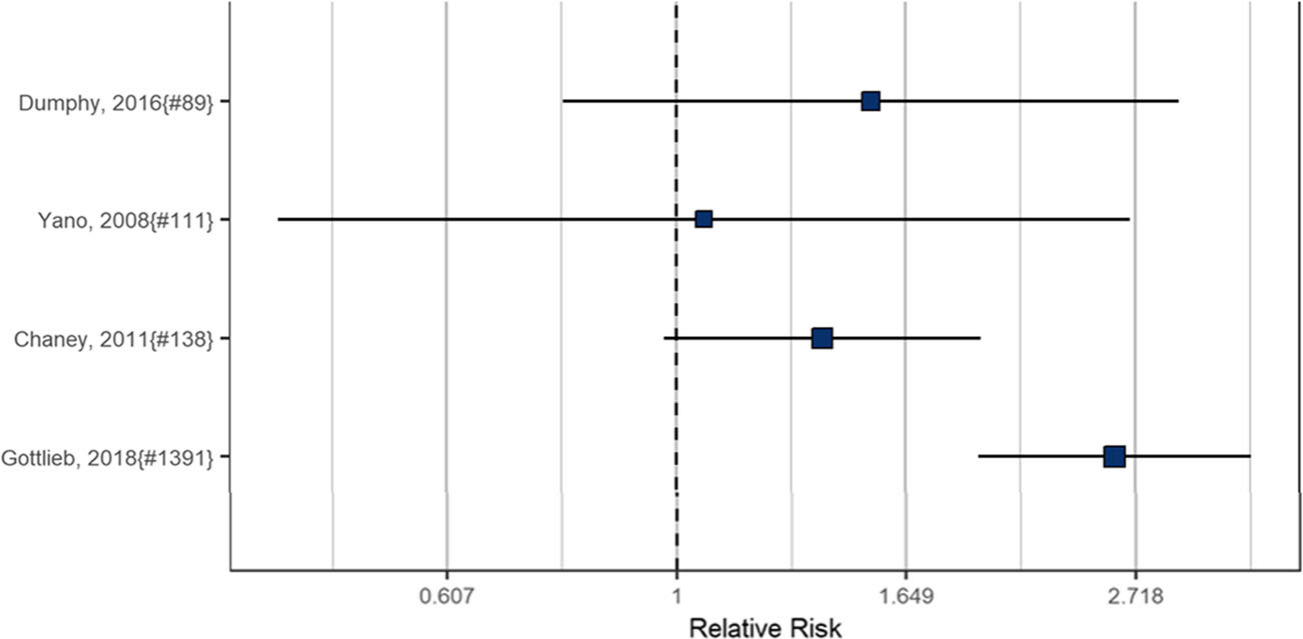
EBQI effectiveness.

We abstracted data from all 14 evaluations of primary care QI initiatives that used the term EBQI and that reported on a patient health outcome (SDC Table 3). Not all studies provided sufficient detail to allow effect size calculation. None of the studies compared two quality improvement strategies in a head-to-head comparison; hence, the documented effectiveness represents the effectiveness of the combined EBQI and implemented intervention. The forest plot in Figure 3 shows effect estimates for four studies reporting categorical outcomes, expressed as relative risk (RR), that could be combined in a meta-analysis. Studies assessed the implementation of a breast-feeding protocol in primary care,^16^ an intervention targeting primary care referrals to smoking cessation clinics,^13^ the implementation of collaborative care for depression,^14^ and a program to increase adherence to immunization guidelines for adults with diabetes.^17^ The effectiveness estimates varied widely by quality improvement target and study, only one of the studies reported a statistically significant effect, but all suggested more improvements in the EBQI group.

## DISCUSSION

The scoping review shows that the evidence base for EBQI is growing, and to our knowledge, this is the first study that provides an overview of the available EBQI literature.

We identified EBQI components and their relative frequency, both across EBQI–labeled studies and in comparison to studies that were similar in approach to EBQI without using EBQI terminology. The focus on evidence at multiple stages of the QI initiative and the strong emphasis on engaging stakeholders were key features.

However, “evidence” was often not systematically described in the identified studies. Not all studies reported a review of the evidence to identify a target for the QI initiative (54% across EBQI–labeled and EBQI–compatible studies). This gap calls into question the focus of these studies on using evidence to identify and define QI aims, a critical entry point for introducing evidence into the QI process. Most, but not all (88%) of the studies reviewed evidence to select and shape the QI intervention design, another critical entry point for applying published research, local data, and implementation science knowledge. More complete reporting on evidence use across studies would promote assessment of fidelity to the EBQI process, which is critical to evaluation of the success of the QI initiative and our ability to learn from initiatives across settings.^18^

Our review also shows that overall, there is still insufficient information regarding the effectiveness of EBQI. We only found a small number of studies using EBQI that reported on key and patient-centered outcomes, i.e., patient health, and studies addressed substantially different intervention targets, ranging from breast-feeding to depression treatment. We did not find studies that compared EBQI with other quality improvement strategies in head-to-head comparisons; hence, the effect of EBQI in the included studies was invariably confounded with the QI content. It is not known yet how EBQI compares to other quality improvement strategies, in particular quality improvement interventions that are based on anecdotal evidence. Future research should evaluate the comparative effectiveness of EBQI to provide more information on this critical aspect.

Our review has several limitations. While we systematically identified all known EBQI publications, we sampled the literature for EBQI–compatible studies and restricted to those published in recent years and limited to primary care given the large QI literature.^19^ The sampling strategy was chosen to obtain a systematic and pragmatic sample that would serve as an exemplar of EBQI–compatible studies. However, it should be noted that earlier approaches were not included, which undoubtedly left out important approaches, and EBQI–compatible approaches in other fields, such as improvements in hospitals in international settings, could have provided additional important information.

We show that EBQI is a promising and growing strategy that aims to integrate prior scientific findings and methods into QI initiatives. Commonly used EBQI features integrate evidence throughout the improvement process, from the initial developmental phase of the QI initiative through to its evaluation. Future research should clearly document EBQI processes to enable better characterization of core initiative features and should assess the comparative effectiveness and success in addressing patient-centered goals.

## Supplementary Information

ESM 1 (DOCX 656 kb)
