# Supplementary material for: Evidence-Based Quality Improvement: a Scoping Review of the Literature
Source: J Gen Intern Med. 2022 Sep 29;37(16):4257–67. doi: 10.1007/s11606-022-07602-5 (PMC9708973; doi:10.1007/s11606-022-07602-5)
Supplement: Supplementary file 1 — (DOCX 656 kb) [file 11606_2022_7602_MOESM1_ESM.docx]

Supplemental Digital Content

Search Strategies

EBQI Search

**PubMed**

Publication dates: not restricted

Date: 3/20/2020

**Strategy:**

"Evidence based quality improvement" OR “Evidence-based quality improvement” OR EBQI

**CINAHL**

Publication dates: not restricted

Date: 3/20/2020

**Strategy:**

"Evidence based quality improvement" OR “Evidence-based quality improvement” OR EBQI

**Scopus**

Publication dates: not restricted

Date: 3/20/2020

**Strategy:**

"Evidence based quality improvement" OR “Evidence-based quality improvement” OR EBQI

Results (deduplicated): 172 citations

Quality Improvement Interventions Search

**PubMed**

Publication dates: 2015-current

Results on 3/13/2020: 587 citations

**Strategy:**

("Diffusion of Innovation"[Mesh] OR "Evidence based quality improvement" OR EBQI OR “implementation strategy” OR “implementation strategies” OR (("Evidence-Based Practice"[MeSH Terms] OR “evidence” OR “evidences”) AND ("Quality Improvement"[Mesh] OR “quality improvement” OR “quality improvements” OR “QI”)))

AND

("Primary Health Care"[Mesh] OR “Primary Care” OR “Primary Health Care” OR “Primary Healthcare”)

**CINAHL**

Publication Dates: 2015-current

Results on 3/13/2020: 1318 citations

**Strategy:**

(MH "Diffusion of Innovation+” OR "Evidence based quality improvement" OR EBQI OR “implementation strategy” OR “implementation strategies” OR ((MH "Professional Practice, Evidence-Based+" OR “evidence” OR “evidences”) AND (MH "Quality Improvement+" OR “quality improvement” OR “quality improvements” OR “QI”)))

AND

(MH "Primary Health Care" OR “Primary Care” OR “Primary Health Care” OR “Primary Healthcare”)

**Scopus**

Publication Dates: 2015-current

Results on 3/13/2020: 617 citations

**Strategy:**

("Evidence based quality improvement" OR EBQI OR “implementation strategy” OR “implementation strategies” OR ((“evidence” OR “evidences”) AND (“quality improvement” OR “quality improvements” OR “QI”)))

AND

(“Primary Care” OR “Primary Health Care” OR “Primary Healthcare”)

Detailed Eligibility Criteria

Inclusion criteria EBQI sample to inform Review Question 1

- Citations using the term “evidence-based quality improvement” or “evidence based quality improvement” in the title, abstract, and keyword of the publication were eligible for inclusion regardless of study design, publication year, participants, intervention, outcome, or setting. We accepted the authors’ definition of EBQI. Publications where EBQI did not stand for evidence based quality improvement (e.g., Ecosystem-Based Quality Index) were excluded. Publications were included regardless of the publication year.

Inclusion criteria broader QI sample (including EBQI) to inform Review Question 2

- Studies meeting KQ1 criteria
  - Studies explicitly describing the approach as EBQI, reporting an evaluation of a quality improvement initiative in primary care were eligible. Searches for these studies were restricted to 2017-2020. We restricted to primary care to identify a more homogenous sample.
- Other studies
  - Study design and publication type: Eligible publications needed to report on results of an intervention and published in the last three years when the term EBQI has been more widely used in publications.^1-21^ Empirical studies could use concurrent (e.g., randomized controlled trials, clinical trials) or historic comparators (e.g., pre-post studies, organizational case series, time series) but studies reporting post-only data were excluded. EBQI publications (see KQ1) that evaluate an intervention in primary care were also eligible regardless of the publication year. We retained relevant systematic reviews for reference-mining.
  - Participants: Eligible publications needed to involve U.S. healthcare professionals and quality improvement in healthcare delivery organizations.
  - Intervention: Publications addressing evaluation of implementing quality improvement initiative(s) in healthcare delivery organizations and learning health systems were eligible. Quality improvement was defined as a structural change in how healthcare is delivered. We excluded educational interventions without any structural changes. Additionally, studies needed to refer to an evidence review in the development of the quality improvement intervention by the healthcare delivery organization. Evidence review was defined as a literature review undertaken for the project, documentation of locally generated data to determine the need for the intervention, and/or utilizing authoritative sources such as clinical practice guideline that were based on an evidence review. Furthermore, we required projects to document continued examination of evidence during or after the implementation of the intervention such as (e.g., using data to evaluate the effects of the intervention; Plan-Do-Study-Act [PDSA] cycles / statistical process control, or other evaluation methods).
  - Outcome: Empirical studies of the effect of a QI intervention that was developed using an evidence-based strategy were eligible. Studies needed to report on structural measures (e.g., organizational changes, costs), process measures (e.g., performance measures such as number of tests ordered; utilization, unintended consequences), and/or outcomes (e.g., health outcomes, patient satisfaction, adverse events). Studies not reporting empirical data from a structured assessment were excluded.
  - Setting: Studies evaluating US primary care interventions were eligible. Studies in secondary care facilities, hospitals, nursing homes, dentist facilities, and laboratories, or other facilities without direct contact to patients were excluded. In addition, we excluded non-US studies.

Inclusion criteria effectiveness sample to inform Review Question 3

- Publication type: Studies reporting effectiveness results for evaluation of an EBQI initiative were eligible for inclusion. Publications meeting both, KQ1 as well as KQ2 criteria, were eligible. Empirical studies may have used concurrent (e.g., randomized controlled trials, clinical trials) or historic comparators (e.g., pre-post studies, organizational case series, time series) to evaluate changes. Studies had to evaluate a primary care intervention. Ineligible studies included: did not use an EBQI method (KQ1), did not use an evidence-based approach to the intervention (KQ2), reported only on measures other than patient health, and conducted in non-primary care settings.

Data Abstraction

Data for the scoping review were abstracted in online software for systematic reviews. Data abstraction forms were piloted and included detailed instructions to reduce ambiguity. One reviewer abstracted the data and a second, experienced evidence reviewer checked the data for accuracy and consistency.

Data extraction Review Question 1 (EBQI studies)

For all EBQI citations we abstracted the author, publication year, unique study identifier, additional publications with more information on the study. Furthermore, the definition of EBQI or the first sentence where EBQI is mentioned; other relevant concepts associated with EBQI; and reference to any other EBQI publication in lieu of defining EBQI. We also abstracted funding by the Department of Veterans Affairs (VA) and any reference to Lisa Rubenstein’s group (known EBQI researchers).

Data abstraction Review Question 2 (EBQI-compatible studies)

We abstracted from the EBQI intervention evaluations as well as the broader EBQI-consistent sample how “evidence” was used according to the study authors. In addition, we abstracted how “evidence” was defined (KQ2a). We abstracted additional information for context including the Study ID, the topic area, the type of outcome, the definition of evidence, the engagement target (who was part of the initiative), and the named quality improvement and implementation strategy where available.

In addition, we recorded EBQI components to answer subquestions 2a and 2b. We abstracted whether ten components were reported:

- Evidence to identify target: Using evidence (data) to identify the target of the QI intervention. A description of local data demonstrating the need for the intervention. Documenting baseline data in the research article was not considered sufficient to meet this criterion.
- Iterative: Iterative, interactive process for selecting the intervention and identifying evidence. Indicating discussions within the organization, interactive selection of the intervention.
- Stakeholder engagement: Reaching out to stakeholders within the organization; collaborative process. Engaging diverse organizational stakeholders; top-down/bottom-up approach.
- Evidence review to identify intervention: Describes a literature review to identify effective interventions in the research literature, finds an authoritative source that recommends specific interventions based on evidence review, or shows existing local data that suggests effectiveness. A background section in the research journal was not considered using evidence, the evidence review had to be described as part of the quality improvement initiative.
- QI facilitation: Use of QI facilitation; Quality improvement facilitation may refer to an external facilitator, an established internal coordinator dedicated to quality improvement facilitation or learning collaborative; providing coaching and mentoring for quality improvement processes. The criterion was met when a quality improvement initiative toolkit was described but not when a (local) champion was designated.
- Leadership involvement: Involving clinical operations or management. These may include medical directors, nurse leadership, primary care leadership, chief medical officer, or nurse managers. Publications had to describe ongoing involvement. To meet this criterion, more than one-time approval or briefing at the end was needed.
- Priority setting with leadership: Setting priorities with clinical operations leadership. Organizational leadership was involved in priority setting for the target of the quality improvement initiative. This domain was added as a result of the consultation exercise.
- Frontline engagement: Engaging frontline personnel early in the quality improvement initiative. Frontline may include clinicians (physician, nurse practitioner, physician assistants), allied clinical staff (nurses, licensed vocational nurses, medical assistants), or administrative staff (e.g., front desk, scheduling). Engagement had to start at selecting the intervention, not just when change was decided.
- Evidence to determine success: Use of evidence (empirical data) to determine the success of the quality improvement initiative. Evaluation data of the intervention (feasibility, intervention adherence, effectiveness, performance measures; quantitative or qualitative data); this may include an iterative approach, PDSA cycles or collaborative learning sessions.
- Analytic support: Use of a dedicated statistician or additional analyst beyond the clinical team providing statistical support to analyze the results of the quality improvement initiative, for example, to support an analysis of multiple data sources, analysis of performance feedback, or formative feedback analysis.

The domains had been developed by a team of quality improvement content experts.

Data abstraction Review Question 3 (EBQI effectiveness)

For EBQI studies meeting inclusion criteria for the review question, we abstracted the study ID, study design, setting, participant characteristics, EBQI / implementation strategy, intervention (clinical area, type, description), outcome and results (number of participants in the intervention group, number of participants in the control group, outcome type, exact measure, follow-up relative to the start of the intervention rollout, results in comparison to the control group [point estimate and measure of dispersion]).

Supplemental Digital Content Table 1: Evidence Table EBQI Definitions

| **Study ID** | **Definition of EBQI** | **Other relevant information**  **OR**  **The first sentence where EBQI is mentioned** | **VA Funds** | **LR Group** | **Other EBQI reference** |
| --- | --- | --- | --- | --- | --- |
| Agee, 2017 ^9^ | N/A | EBQI in subtitle, referencing the chronic care model as the evidence basis for the QI program | No | No | NA |
| Ainsworth, 2014 ^22^ | N/A | "Evidence-based quality improvement" only appears in the abstract of this paper under "Design" | No | No | N/A |
| Arnold, 2014 ^23^ | N/A | "Evidence-based quality improvement" only appears in the title of this paper. | No | No | N/A |
| Bacidore, 2017 ^10^ | N/A | EBQI in subtitle | No | No | NA |
| Badru, 2017 ^24^ | N/A | "The purpose of this EBQI project was to create a clinical practice guideline based on social cognitive theory to provide new strategies for managing veterans with PTSD" | Not VA funded but in the VA (DNP project) | No | N/A |
| Bashford, 2005 ^25^ | N/A | "There is a small but growing evidence base for QI in low resource settings, but this needs to be locally accessible to allow self-sustaining evidence-based quality improvement." | No | No | No |
| Bauer, 2016 ^4^ | N/A | This quality improvement project used best available evidence for skin care in patients undergoing radiation therapy and a theoretical framework, 4MATing, to provide a structured approach to patient education designed to enhance adherence to skin care recommendations. | No | No | N/A |
| Becker, 2018 ^26^ | N/A | Through a series of courses, students develop and conduct an evidence?based quality improvement (EBQI) project. | No | No | N/A |
| Bennett, 2016 ^27^ | N/A | "Evidence-based quality improvement" term in the abstract of citation but does not appear in the full-text. | No | No | N/A |
| Berman, 2015 ^28^ | N/A | This article guides the reader through the process of interacting with administrative leadership to gain support for evidence-based quality improvement endeavors. | No | No | N/A |
| Berta, 2019 ^29^ | N/A | "Evidence-based quality improvement" is only mentioned in the title, and is not used in the full-text, although "facilitation quality improvement" and "evidence-based practice interventions" are used. | No | No | N/A |
| Blakeman, 2015 ^1^ | N/A | The results of this project revealed that practicing nurses on telemetry units can implement fairly straightforward and simple evidence-based quality improvement projects and make a positive impact on unit practice. | No | No | N/A |
| Bonner, 2005 ^30^ | N/A | N/A | Yes | In Acknowledgements | N/A |
| Briscoe, 2018 ^31^ | N/A | This project examined the effectiveness of an evidence-based quality improvement process in providing post-discharge telephone follow up to adult patients on hemodialysis by experienced nephrology nurses through standardized unit workflow and leveraging of the electronic medical record (EMR). | No | No | N/A |
| Britto, 1997 ^32^ | N/A | "Using evidence-based quality improvement techniques, including literature synthesis and presentation of clinic-specific data" | No | No | N/A |
| Brown, 2016 ^33^ | N/A | This paper outlines the development and psychometric evaluation of an Internet survey to measure features of clinical networks and provides descriptive results from a sample of members of 19 diverse clinical networks responsible for evidence-based quality improvement across a large geographical region. | No | No | No |
| Burden, 2017 ^34^ | EBQI is an adaptation of the Plan-Do-Study-Act (PDSA) cycle of continuous quality improvement (CQI) that emphasizes empirical evidence and the co-involvement of clinical and implementation experts (i.e., researchers) and an organization’s own healthcare professionals; EBQI builds upon traditional CQI techniques (e.g., team-based approach and PDSA cycle) by incorporating outside clinical and implementation experts to ensure that QI efforts are evidence-based while also emphasizing the involvement of an organization’s own healthcare professionals and staff improving their own systems. More specifically, researchers contribute knowledge of the evidence base about a particular evidence-based practice, as well as materials, procedures, and tools needed for successful implementation.; Local clinicians and administrators contribute local knowledge needed to tailor the evidence-based practice for their own particular needs and organizational capabilities.; In addition to providing expertise, researchers in the EBQI model also facilitate problem solving and provide ongoing technical support for developing data collection/analysis tools, informatics tools, and training materials. EBQI fosters a researcher/clinician partnership and promotes buy-in.” | N/A | Yes | Yes | Curran, 2008^35^ |
| Chaney, 2008 ^36^ | N/A | Implementation research in health care is characterized by a focus on evaluating evidence-based quality improvement and often involves partnerships between researchers and clinicians across multiple sites | Yes | Yes | N/A |
| Chaney, 2011 ^37^ | Evidence-based quality improvement (EBQI) uses QI approaches within a research/clinical partnership to redesign care A more structured form of CQI. | "The study intervention is EBQI as applied to CCM implementation". | Yes | Chaney, 2008 | Shojania, 2005 |
| Chien, 2019 ^38^ | N/A | "In addition to innovation and new breakthrough discoveries, curriculum development and EBQI projects, although may not seem so novel, are also important areas of nursing research" | No | No | N/A |
| Ciocson, 2018 ^39^ | N/A | A nurse-led evidence-based quality improvement study was conducted in a pediatric outpatient clinic of a tertiary university hospital in Riyadh, Saudi Arabia | No | No | None |
| Cohen, 2013 ^40^ | Evidence-based quality improvement (EBQI) aims to systematically incorporate evidence and data regarding care provision and patient outcomes into quality improvement efforts. | N/A | Yes | Fortney, 2012 | Shojania, 2004 |
| Cordasco, 2015 ^41^ | N/A | Given the variability in EDs’ intrinsic characteristics, needs, and local contexts, evidence-based quality improvement methods, which engage and harness the creativity of local leaders to construct interdisciplinary solutions addressing overall organizational priorities, would be well suited for building this capacity | Yes | Yes | N/A |
| Curran, 2008 ^35^ | The goals of EBQI are to: 1) ensure evidence-based clinical care, 2) tailor the care model to local conditions, 3) minimize clinician time spent on materials or procedure development, and 4) ensure development of local expertise in implementing the care model”; EBQI fosters an active researcher-clinician partnership and takes advantage of features known to facilitate innovation, including directly working through the decision-making process regarding intervention design with organizational stakeholders, local adaptation, use of diffusion networks (i.e., opinion leaders), and involvement of researchers as change agents | Operationalization of the EBQI model for this study included: Local development teams made up of clinicians and administrators from each site and the study PI. Meetings held by conference call over a series of 8 weeks. Consideration of barrier/facilitator data from observations/ interviews Drafting of a locally-customized clinical care and implementation strategy Expert consultation on the clinical care and implementation strategies Iterative re-drafting of the strategies until Development Panel members and experts "approved" the interventions. "Launch date" planning | Yes | Yes | Rubenstein, 2002^42^ |
| Curran, 2011 ^43^ | EBQI builds upon traditional Continuous Quality Improvement techniques by incorporating input from outside clinical experts to ensure that implementation efforts are evidence-based. It engages local participants in the planning and implementation process; EBQI fosters a researcher/clinician partnership and promotes clinician buy-in; Researchers contribute knowledge from the clinical and implementation science evidence bases as well as materials, procedures, and tools needed for successful implementation.; Clinicians and administrators contribute local knowledge needed to tailor the evidence-based practices for their own contexts. | Plan-Do-Study-Act rapid cycles were used during the EBQI process to adapt the intervention and tailor implementation efforts | Yes | Yes | N/A |
| Dick, 2019 ^44^ | N/A | "Evidence-Based Quality Improvement" only appears in the title. | No | No | N/A |
| Driscoll, 2011 ^45^ | N/A | The Heart Failure Intervention Score provides a potential evidence?based quality improvement tool through which a set of minimum standards can be developed. | No | No | N/A |
| Dumphy, 2016 ^46^ | N/A | The overall aim of this evidence-based quality improvement (QI) project was to increase breastfeeding rates by refining the care provided to a diverse patient population with historically low breastfeeding rates. | No | No | N/A |
| Epstein, 2013 ^47^ | N/A | An evidence-based quality improvement intervention has been developed that effectively improves the quality of ADHD care delivered by community-based pediatricians | No | No | N/A |
| Estes, 2014 ^48^ | N/A | The COPD case-finding program was planned as an evidence-based quality improvement project with a prospective, descriptive design | No | No | N/A |
| Faris, 2015 ^49^ | N/A | We examined the presurgical evaluation of suspected lung cancer patients in a community-based health care system to establish current benchmarks of care that will lay the groundwork for an evidence-based quality improvement project. | No | No | N/A |
| Feng, 2018 ^50^ | N/A | This study will address the gap in research by adapting and evaluating an Aged Care Clinical Mentoring Model that has been tested as an effective model of change in evidence-based quality improvement in nursing homes | No | No | N/A |
| Fortney, 2012 ^51^ | EBQI was developed by Rubenstein and colleagues based on the findings of the Mental Health Awareness Project, which compared two quality-improvement strategies for depression in primary care (characterized by centralized strategic decision making and local tactical decision making and incorporating both top-down and bottom-up approaches hold the most promise for sustained implementation of evidence-based practices). In EBQI, both researchers (clinical experts, implementation experts) and local staff participate fully in the quality-improvement process, with the researchers facilitating rather than dictating implementation efforts. It promotes buy-in from leadership and while emphasizing the involvement of outside experts and empirical evidence, EBQI stresses that an organization's own healthcare professionals and staff are best positioned to improve their systems. EBQI also emphasizes continuously revising the adapted evidence-based practice based on feedback during Plan-Do-Study-Act cycles and, thus, should lead to adapted evidence-based practices that are robust, user friendly, and feasible to deploy in real-world practice settings. | N/A | Yes | Yes | N/A |
| Fortney, 2013 ^52^ | "EBQI was developed by Rubenstein and colleagues based on the findings of the Mental Health Awareness Project, which compared two quality-improvement strategies for depression in primary care. Centralized strategic decision making and local tactical decision making.Incorporating both top-down and bottom-up approaches hold the most promise for sustained implementation of evidence-based practices.In EBQI, both researchers (clinical experts, implementation experts) and local staff participate fully in the quality-improvement process, with the researchers facilitating rather than dictating implementation efforts. Promotes buy-in from leadership. While emphasizing the involvement of outside experts and empirical evidence, EBQI stresses that an organization's own healthcare professionals and staff are best positioned to improve their systems. EBQI also emphasizes continuously revising the adapted evidence-based practice based on feedback during Plan-Do-Study-Act cycles and, thus, should lead to adapted evidence-based practices that are robust, user friendly, and feasible to deploy in real-world practice settings." | N/A | Yes | Fortney, 2012 | N/A |
| Fox, 2016 ^5^ | Involves adapting evidence-based practices to meet local needs -can improve the uptake of evidence-based programs and practices into routine practice. -EBQI uses bottom-up engagement of local site personnel to adapt the evidence base to the local context through consensus development and group decision–making among researchers, key stakeholders, and local-level providers and staff. A key tenet of EBQI is that local hospital leadership and clinical staff are best equipped to determine how to improve outcomes within their health care system -EBQI engages managers and staff in adapting evidence to local contexts, embedding an efficacious training in a large organizational change initiative with multi-level involvement and support. -EBQI also has the potential to enhance the effectiveness of evidence-based innovations by tailoring them to the specific needs and preferences of local site staff. | N/A | Yes | Fortney, 2012 | N/A |
| Gadbois, 2016 ^53^ | N/A | "The guiding framework for this evidence-based performance improvement project was the (PDSA) cycle" | No | No | N/A |
| Gammack, 2012 ^54^ | N/A | "Using EBQI initiatives, meaningful clinical outcomes have been demonstrated for elderly populations across a multitude of care settings and across the world." | No | No | N/A |
| Gellert, 2019 ^19^ | N/A | Did not include “evidence-based quality improvement” anywhere in abstract or full text. | No | No | N/A |
| Giberson, 2017 ^55^ | N/A | "This EBQI project aimed to determine whether implementation of a 'time out tool' for emergency intubations would improve patient safety and outcomes" | No | No | N/A |
| Goldstein, 2018 ^14^ | Uses a systematic, multilevel approach to incorporating scientific findings into clinical settings driven by the partnership of researchers and local healthcare leaders, managers and clinical staff. -By enlisting clinical partners from organizational leadership and quality improvement (QI) teams using a “top-down and bottom-up” approach, EBQI infuses evidence into a structured process that is relevant and specific to local organizational needs and resources -makes use of behavior change theory, coupled with rigorous measurement strategies and formal feedback to local partners at all levels, to fuel the process of bringing objective evidence to the clinical setting -partners tailor the delivery of a particular intervention according to their environment while keeping the crucial elements of the evidence-base intact. | N/A | Yes | Yes | Fortney, 2012^51^ |
| Goodman, 2017 ^56^ | N/A | As evidence-based quality improvement is an iterative strategy requiring alternating action and reflection, it is important for all stakeholders to understand systems and the problems they create, while developing and evaluating solutions to these concerns | No | Hamilton, 2017 | N/A |
| Gottlieb, 2018 ^57^ | N/A | "The purpose of this EBQI project was to implement processes to facilitate provider's adherence to immunization guidelines for adults with T2DM." | No | No | N/A |
| Goulding, 2015 ^58^ | N/A | A collaborative evidence-based quality improvement project was therefore undertaken by the iMobile team at KCH in conjunction with researchers from King's Improvement Science (KIS). Plan, Do, Study, Act (PDSA) methodology was used. Three PDSA cycles were undertaken. | No | No | N/A |
| Griffin, 2014 ^59^ | N/A | "Evidence-based quality improvement" only appears in the abstract of this paper under "Design" | No | No | N/A |
| Grimshaw, 2006 ^60^ | N/A | Makes a case for EBQI but does not define it | No | No | N/A |
| Güreşen, 2020 ^61^ | N/A (EBQI stands for Ecosystem Based Quality Index) | NA | NA | NA | NA |
| Gwatirisa, 2015 ^62^ | N/A | An evidence-based QI project (EBP QI) was designed and implemented as a review of paper and electronic charts and documentation with subsequent data analysis. | No | No | N/A |
| Hameed, 2010 ^63^ | N/A | In the near future, cooperation of ACS services from community and academic hospitals across the country will lead to the formation of systems of acute surgical care whose development will be informed by rigorous data collection and research and evidence-based quality-improvement initiatives. | No | No | N/A |
| Hamilton, 2017 ^12^ | N/A | Response to the commentary provided by Goodman and Sanders Thompson about Hamilton, 2017 | N/A | N/A | N/A |
| Hamilton, 2017 ^11^ | EBQI involves engaging multilevel, inter-professional leaders and staff as stakeholders in reviewing evidence and setting QI priorities. It is a systematic implementation strategy that fosters multilevel, inter-professional engagement. EBQI is a form of continuous quality improvement designed specifically to improve uptake of evidence-based programs (EBPs) in routine practice. To promote evidence-based priority setting for development of local site level QI efforts, EBQI researchers assemble key literature and local data on the problem to be addressed. To set priorities, stakeholder groups come to consensus using modified Delphi methods. The consensus process is informed by both data and facilitated discussion. | Rubenstein and colleagues describe four key components of EBQI: (1) research-clinical partnerships; (2) top-down (leaders) and bottom-up (staff) engagement of local site personnel to adapt EBPs to the local context through consensus development and group decision-making among researchers and local-level leaders, providers, and staff; (3) use of research evidence for QI targets and behavior change methods; and (4) ongoing support for EBQI teams from researchers serving as technical experts | Yes | Fortney, 2012  Yano, 2016  Yoon, 2016 | N/A |
| Hanna-Bull, 2016 ^64^ | N/A | An interdisciplinary skin and wound care team designed an evidence-based quality improvement initiative based on a systematic literature review and standardization of heel offloading methods. | No | No | N/A |
| Harrison, 2019 ^65^ | N/A | The novel model promotes a decentralized care program that brings emergency obstetric care to women instead of the converse through four concepts: the care should be cloSe(community-based), it should be very dedicated to Action (transfer of care), it should be Focused on and highly specific to labor and delivery (cesarean birth center), and finally, it should be committed to high-quality care through iterative Evidence-based quality improvement programming and data collection. | No | No | N/A |
| Horbar, 1999 ^66^ | N/A | Building on the lessons learned in that initial project, the Network now is conducting the Vermont Oxford Network Evidence-Based Quality Improvement Collaborative for Neonatology, known as NIC/Q 2000. | No | No | N/A |
| Horbar, 2006 ^67^ | NA | "Evidence-based quality improvement" only appears in title | No | No | N/A |
| Hunt, 2012 ^68^ | the EBQI model was developed which involves both centralized strategic decision-making and local tactical decision-making - In EBQI, both researchers (clinical experts, implementation experts) and local staff participate fully in the quality improvement process, with the researchers facilitating rather than dictating implementation efforts - EBQI is intended to foster a researcher/clinician partnership that promotes buy-in - While emphasizing the involvement of outside experts and empirical evidence, EBQI stresses that an organization’s own healthcare professionals and staff are best positioned to improve their systems -Clinicians and administrators contribute local knowledge needed to tailor the evidence based practice for their own particular needs and organizational capabilities. Researchers contribute knowledge of the evidence base and tools needed for successful implementation. -In addition to providing expertise, researchers in the EBQI model also facilitate problem solving and provide ongoing technical support. -EBQI also emphasizes continuously revising the adapted evidence based practice based on feedback during Plan-Do-Study-Act cycles, and thus should lead to adapted evidence based practices that are robust, user-friendly, and feasible to deploy in real-world practice settings. | EBQI was developed by Rubenstein and colleagues based on the findings of the Mental Health Awareness Project, which compared two quality improvement strategies for depression in PC | No | Yes | N/A |
| Hurwitz, 2015 ^69^ | N/A | In response, a group of ED staff nurses at a multihospital health system conducted an evidence-based quality improvement project to determine the best practice for accurate temperature measurement in children younger than five years who presented to the ED. | No | No | N/A |
| Huynh, 2016 ^6^ | N/A | Stepped wedge designs have gained recognition as a method for rigorously assessing implementation of evidence-based quality improvement interventions (QIIs) across multiple healthcare sites. | Yes | Yoon, 2016 | N/A |
| JOGNN, 2016 ^70^ | No definition provided | Conference proceedings of the 2016 Association of Women's Health, Obstetric and Neonatal Nurses (AWHONN) convention, poster presentations for "Evidence-Based Quality Improvement Projects". | No | No | N/A |
| JOGNN, 2017 ^71^ | No definition provided | Conference proceedings of the 2017 Association of Women's Health, Obstetric and Neonatal Nurses (AWHONN) convention, poster presentations for "Evidence-Based Quality Improvement Projects". | No | No | N/A |
| Kasting, 2018 ^72^ | N/A | To increase rates, the CDC recommends clinics adhere to components of their evidence-based quality improvement program, AFIX (Assessment, Feedback, Incentives, and eXchange of information). | No | No | N/A |
| Katon, 2008 ^73^ | N/A | To describe evidence-based quality improvement interventions in the primary care system that have been shown in randomized trials to the improve quality of care and outcomes of patients with depression. | No | No | N/A |
| Katona, 2005 ^74^ | N/A | Evidence based Quality Improvement was one of the author's key words | No | No |  |
| Khalil, 2017 ^13^ | N/A | Editorialin which the author clarifies the scope of the journal and its requirements for reporting articles submitted to the journal for publication. | No | No | N/A |
| Kilbride, 2003 ^75^ | N/A | Six neonatal intensive care units (NICUs) that are members of the Vermont Oxford National Evidence-Based Quality Improvement Collaborative for Neonatology collaborated to reduce infection rates | No | No | N/A |
| Kilbride, 2003 ^76^ | N/A | Six neonatal intensive care units (NICUs) that are members of the Vermont Oxford National Evidence-Based Quality Improvement Collaborative for Neonatology collaborated to reduce infection rates. | No | No | N/A |
| Kitson, 2000 ^77^ | N/A | -What to do, how to do it, who should do it and whether the systems and processes are available to do it correctly are the basis of new ways of thinking about evidence-based quality improvement. | No | No | N/A |
| Klause, 2020 ^78^ | N/A | The purpose of this evidence-based quality improvement project was to improve the rate of NPs addressing IPV during annual women’s health visits | No | No | N/A |
| Kleczka, 2018 ^79^ | N/A | These data can form the basis for evidence-based quality improvement efforts at large scale, and help deliver on the SDG promise of quality essential healthcare services for all. | No | No | N/A |
| Koenig, 2016 ^80^ | EBQI methods use techniques derived from quality improvement to ensure that study implementation is consistent with the intervention evidence base and sufficiently acceptable to stakeholders. EBQI also helps foster partnerships among researchers, clinicians, staff, and administration to help align goals and expectations over the course of the study period. | N/A | Yes | Fortney, 2012; Hamilton, 2013 | N/A |
| Kramer, 2017 ^81^ | N/A | The EBQI strategy evolved from earlier studies of quality improvement for depression25– 27 consisting of centralized strategic decision- making about which EBPs to implement in combination with local tactical decision- making about how to adapt and deploy the specific EBPs. | No | Fortney, 2012 | N/A |
| Krug, 2016 ^82^ | N/A | The primary purpose of this evidence-based quality improvement practice project was to increase anesthesia providers’ knowledge and awareness of the common elements of the taping practice for securing the ET tube that increase the patient’s exposure to pathogens and the risk of nosocomial infection. | No | No | N/A |
| Le Flore, 2017 ^83^ | N/A | "The purpose of this project was to evaluate an EBQI program implemented in 2016 in a clinical practice setting to reduce overprescribing of opioids for non-cancer pain management." | No | No | No |
| Lennox, 2001 ^84^ | N/A | In fact, these developments suggest the substance abuse treatment field has the potential to move not only toward a research-based quality improvement system that accommodates the quality management demands, but also to an evidence-based quality improvement (EBQI) system--a system that is capable of tracking, scoring, and feeding back quality-of-care (QOC) information to providers, as well as using near real-time collected data to research continuously the quality-related elements of substance abuse treatment over time | No | No | N/A |
| Levine, 2016 ^85^ | N/A | EBQI only appears in the title of this conference abstract | No | No | N/A |
| Lipshutz, 2008 ^86^ | N/A | Did not define EBQI explicitly, but article is regarding QI initiatives using PDSA cycles | No | No | N/A |
| Lockwood, 2020 ^87^ | N/A | "EBQI/evidence-based quality improvement" only in the title | No | No | N/A |
| Loftus, 2015 ^88^ | N/A | This study describes the successful application of the Define-Measure-Analyze-Improve-Control model in the creation and implementation of evidence-based quality improvement. | No | No | N/A |
| Lozito, 2018 ^89^ | N/A | The purpose of this evidence?based quality improvement project was to encourage personnel in the perioperative department, which included the preoperative, intraoperative, and postoperative areas, to report good catches, learn from these reports, and institute process improvements. | No | No | N/A |
| Mainz, 2010 ^90^ | NA | Modern quality development should be evidence-based, so that clinical practice rests on a well-documented basis | No | No | NA |
| Manheim, 2016 ^91^ | N/A | An evidence-based quality improvement project with two PDSA cycles was implemented to increase the completion rate of advance directives. | No | No | None |
| Manns, 2013 ^92^ | N/A | We identified important barriers to achieving optimal outcomes in diabetes that may be overcome through the use of evidence-based quality improvement interventions | No | No | N/A |
| Mapes, 2005 ^93^ | N/A | "Fistula First" is a nationwide, evidence-based, quality improvement effort that is striving to improve vascular access outcomes by increasing the percentage of patients who dialyze with arteriovenous fistulae | No | No | N/A |
| Marang-van de Mheen, 2016 ^94^ | N/A | The central line bundle to reduce central line-associated bloodstream infections (CLABSI) is widely regarded as one of the most evidence-based quality improvement (QI) interventions. | No | No | Shojania, 2005 |
| Maru, 2012 ^95^ | N/A | Implement evidenced-based quality improvement programs: There is an acute need to close the "implementation gap" in global healthcare delivery--that disconnect between what is known and what is actually available in resource-limited settings | No | No | N/A |
| Marx, 2018 ^96^ | N/A | There is an increasing demand – not only in Kenya – to implement evidence-based QI across health systems to ensure that QI approaches, standards and indicators adhere to scientific standards | No | No | N/A |
| Massengale, 2020 ^97^ | N/A | This is a book review for a book titled "Intervention Research and Evidence-Based Quality Improvement: Designing, Conducting, Analyzing, and Funding, 2nd Edition"; the term "EBQI" only appears in the title. | No | No | None |
| Matulewicz, 2015 ^98^ | N/A | RC is a complex and morbid procedure that may benefit from care redesign. Evidence based quality improvement is integral to this process. | No | No | N/A |
| McAllen, 2018 ^99^ | N/A | " A limitation of this project was that the EBQI design prevents generalization of findings to other settings" | No | No | N/A |
| McCarthy, 2013 ^100^ | N/A | Many studies have commented on the varying levels of care and outcomes of these admissions and highlighted the need for more systematic, consistent, evidence-based quality improvement approaches to improving outcomes from COPD exacerbations. | No | No | N/A |
| McFrederick, 2015 ^101^ | N/A | "Aligned with the CCM, the Assessment of Chronic Illness Care is an evidence-based, practical QI tool that aids organizations in assessing their chronic care delivery system's strength and weaknesses across six CCM categories." | No | No | N/A |
| Melnyk, 2015 ^2^ | N/A | In the effort to deliver high quality, safe and cost‐effective care, quality improvement (QI) and evidence‐based practice (EBP) have become common strategies to drive enhancements in clinical practice and improvements in patient outcomes. | No | No | N/A |
| Mendel, 2011 ^102^ | N/A | Specifically activating interest and participation in CPIC’s randomized trial of two different ways to implement evidence-based quality improvement (QI) programs for depression across diverse community agencies | No | No | N/A |
| Meredith, 2018 ^15^ | EBQI aims to engage front-line clinical teams in developing innovations that reflect interdisciplinary input and are aligned with multi-level healthcare system leadership priorities-EBQI promotes cross-discipline, data-driven problem solving in local primary care practices. -EBQI aligns these local practices with organizational priorities to sustain successful QI innovations over time and spread them across teams and clinics. -Specifically, the EBQI intervention focused on engaging and empowering front-line primary care teams with multi-level, interdisciplinary stakeholders in structured EBQI, and facilitated provider and staff initiated innovation projects. | N/A | Yes | Fortney, 2012Stockdale, 2018Yano, 2016Yoon, 2016 | N/A |
| Meyer, 2018 ^16^ | N/A | Title states Evidence-Based Quality Improvement | No | No | N/A |
| Montgomery, 2018 ^103^ | NA | "A number of evidence-based quality improvement frameworks have been implemented to address patient safety." | No | No | N/A |
| Moore, 2017 ^104^ | N/A | An evidence-based quality improvement project made AA available to all adult surgical patients, and the results were recorded in a database. | No | No | N/A |
| Morrison, 2007 ^105^ | N/A | "Evidence-based quality improvement" term in the citation, no other mention | No | No | N/A |
| Motz, 2016 ^106^ | N/A | "The purpose of this EBQI project was to evaluate reduction of readmission rates after implementation of the Rothman Index in team discharge decision-making." | No | No | N/A |
| Nichols, 2008 ^107^ | Did not provide specific EBQI definition | The abstract states The National Registry of Cardiopulmonary Resuscitation was developed with a goal to enhance patient safety and reduce patient disability and death through "providing an evidence-based, quality improvement program of patient safety." | No | No | N/A |
| Ong, 2017 ^108^ | N/A | "We discuss an EBQI project to evaluate the outcome of a newly implemented shared governance environment on RN engagement and turnover. " | No | No | N/A |
| Owen, 2013 ^109^ | N/A | The EBQI/F [facilitation] intervention, which involves researchers partnering with clinical stakeholders, is designed in a manner that is consistent with available research evidence and reflects the knowledge and expertise of site participants regarding how to tailor the strategy to local needs, priorities and resources. -The intervention combines: EBQI processes in the EBQI Design Phase to design and tailor the initial implementation strategy to the local context, and external facilitation to support, monitor, problem-solve and refine implementation (as needed) during the study’s Implementation Phase. | Yes | Yes | N/A |
| Palm, 2012 ^110^ | N/A | Developed metrics demonstrated that the surveillance capacity and evidence-based quality improvement initiatives disseminated through the JTTS were associated with improved identification and mitigation of complications following battlefield injury. | No | No | N/A |
| Palm, 2012 ^111^ | N/A | Developed metrics demonstrate that evidence-based quality improvement initiatives disseminated through the JTTS were associated with improved clinical practice of resuscitation following battlefield injury. | No | No | N/A |
| Personnic, 2014 ^112^ | N/A (EBQI stands for ecosystem-based index of the quality) |  |  |  |  |
| Post, 2009 ^113^ | N/A | Similarly, efforts to implement sustainable evidence-based quality improvement (QI) strategies for depression cannot occur without an understanding of the relevant organizational contexts within primary care practices | Yes | Yes | N/A |
| Potier, 2015 ^3^ | N/A | Did not include “evidence-based quality improvement” anywhere in abstract or full text. | No | No | N/A |
| Prince, 2014 ^114^ | N/A | To improve the overall performance of our hospital’s code team, we implemented an evidence-based quality improvement restructuring plan. | No | No | N/A |
| Pulver, 2011 ^115^ | N/A | -Drug usage evaluation (DUE) is an evidence-based quality improvement methodology, designed to improve the quality, safety and cost-effectiveness of drug use | No | No | N/A |
| Qureshi, 2016 ^116^ | N/A | In the United Kingdom, General Practitioners are incentivised through a national Pay-forPerformance scheme to adopt evidence-based quality improvement initiatives through a portfolio of Quality and Outcomes Framework indicators. | No | No | N/A |
| Radbron, 2019 ^20^ | N/A | Did not include “evidence-based quality improvement” anywhere in abstract or full text. | No | No | N/A |
| Rahman, 2017 ^117^ | N/A | The setting was an evidence-based quality improvement programme at two hospitals | No | No | N/A |
| Rastorgueff, 2015 ^118^ | N/A (EBQI stands for Ecosystem-Based Quality Index) |  |  |  |  |
| Reiter, 2007 ^119^ | N/A | Investments in qualiyt enhancing interventions is a critical threshold issue to widespread adoption of evidence-based quality improvements | No | No | N/A |
| Rizzo, 2018 ^120^ | "Evidence-based health care forms and implements treatment plans based on current knowledge derived from evidence based research and expert opinion" | Evidence-based Quality Improvement Project | No | No | N/A |
| Rubenstein, 2006 ^121^ | N/A | "We termed the modification evidence-based quality improvement (EBQI), and evaluated its impacts by assessing the performance of experimental and usual care practices on measures of depression related care and outcomes." | Yes | Sherman, 2004 | N/A |
| Rubenstein, 2010 ^122^ | EBQI is a continuous quality improvement approach whose goal is translation of research on care delivery models into routine practice. -EBQI is a more structured form of CQI that (1) incorporates a research/clinical partnership (2) uses top-down and bottom-up features to engage organizational senior leaders and quality improvement teams in adapting and implementing improvements and (3) focuses on prior research evidence not only in terms of clinical guidelines for treatment, but in terms of previously validated care models and provider behavior change methods that promote adherence to appropriate treatment-Researchers worked in partnership with clinical and administrative leaders, and had no authority over clinical implementation decisions.-PDSA cycles involve planning (Plan), carrying out (Do), evaluating success (Study), and putting in place (Act) improvements. | N/A | Yes | Yes | N/A |
| Rubenstein, 2014^123^ | EBQI promotes bottom-up local innovation and spread within top-down organizational priorities. EBQI innovations are supported by a research-clinical partnership, use continuous quality improvement methods, and are developed in regional demonstration sites.  -A multilevel approach that has the potential to focus and empower local QI innovation. The approach integrates system-level and region-level improvement priorities with locally driven, bottom-up, evidence-based problem-solving that is supported by embedded health services researchers. By doing so, EBQI aims to promote both evidence-enriched local innovation and a culture in which local problem-solving is the norm. | N/A | Yes | Fortney, 2012 | N/A |
| Salera-Vieira, 2016 ^124^ | N/A | EBQI only appears as the title of the poster session for conference | No | No | N/A |
| Sangam, 2019 ^125^ | N/A | In conclusion, the awareness of the problem with evidence-based quality improvement measures may help in early detection of intravenous infiltrates and decrease the severe intravenous infiltration in infants. | No | No | N/A |
| Santiago, 2012 ^126^ | N/A | "EBQI" only appears in the title | No | No | None |
| Sarff, 2019 ^21^ | N/A | Did not include “evidence-based quality improvement” anywhere in abstract or full text. | No | No | N/A |
| Saunders, 2015 ^127^ | N/A | The goal of knowledge translation is evidence?based quality improvement to raise the consistency and quality of care delivery. | No | No | Grimshaw, 2006^60^ |
| Schultz, 2005 ^128^ | N/A | It is the premise of this evidence-based quality improvement project that IV cannulas should be removed based on clinical evaluation rather than a pre-set time. | No | No | N/A |
| Schwing, 2019 ^129^ | N/A | This evidence-based quality improvement project set out to determine the most efficient method of Emergency Medical Services (EMS) intake. | No | No | N/A |
| Sherman, 2004 ^130^ | N/A | The team used a "central expert" approach, wherein content experts helped adapt the guidelines locally in an effort to decrease the amount of time and the number of staff necessary to come up with a QI plan . It involved 4 steps: setting priorities, expert adaptation of priorities, development of QI plan, implementation of QI plan | Yes | Yes | Rubenstein, 2002^42^ |
| Shojania, 2005 ^131^ | N/A | Makes a case for EBQI but does not define it | No | No | N/A |
| Smith, 2003 ^132^ | N/A | "Evidence based quality assessment and improvement are a reasonable paradigm for healthcare QI" | No | No | N/A |
| Smith, 2008 ^133^ | The EBQI process allows tailoring of collaborative care implementation to local priorities and resources, while maintaining fidelity to the evidence base for model design EBQI takes advantage of features known to facilitate innovation, including directly working through decision-making processes with organizational stakeholders, allowance for contextual adaptation that does not result in deviations from the evidence base, and involvement of researchers as change agents | N/A | Yes | Yes | N/A |
| Soltis, 2018 ^17^ | N/A | "To address this gap in care, we used the Institute for Healthcare Improvement’s Model for Improvement as the framework to guide the planning, implementation, and evaluation phases of an evidence-based practice quality improvement project. " | No | No | N/A |
| Starkey, 2016 ^134^ | N/A | Although barriers to improving depression care exist, this study suggests that evidence-based quality improvement programs can positively affect practice. | No | No | N/A |
| Stearns, 2018 ^135^ | N/A | Enhanced recovery after surgery (ERAS) protocols are evidence-based quality improvement pathways reported to be associated with improved patient outcomes, specifically shortened length of hospital stay (LOS) and decreased incidence of postoperative complications for several types of surgery. | No | No | N/A |
| Stetler, 2008 ^136^ | N/A | QUERI used an evidence-based organizational framework focused on three contextual elements: 1) cultural norms and values, in this case related to the role of health services researchers in evidence-based quality improvement; 2) capacity, in this case among researchers and key partners to engage in implementation research; 3) supportive infrastructures to reinforce expectations for change and to sustain new behaviors as part of the norm | Yes | Yes | N/A |
| Stevans, 2015 ^137^ | N/A | "The purpose of this case report is threefold: (1) to outline the historical implementation of an evidence-based quality improvement project, (2) to describe the program's future direction using a systems perspective to identify implementation barriers, and (3) to provide implications for the profession as it works toward closing the evidence-to-practice gap." | No | No | N/A |
| Stevens, 2011 ^138^ | N/A | Did not include “evidence-based quality improvement” anywhere in abstract or full text. | No | No | N/A |
| Stevens, 2013 ^139^ | N/A | The call for evidence-based quality improvement and healthcare transformation underscores the need for redesigning care that is effective, safe, and efficient | No | No | Shojania, 2005 |
| Stockdale, 2018 ^18^ | Successful quality improvement implementation requires an organizational infrastructure that supports alignment of leadership priorities and resources with frontline problem-solving, development of new relationship systems that bridge organizational boundaries, and a culture shift favorable toward quality improvement. Specifically, the design elements address familiar quality improvement problems, including lack of interdisciplinary leadership for quality improvement alignment of top management, quality improvement priorities with locally developed quality improvement projects, efficient quality improvement resource management/use, and dissemination of effective solution | N/A | Yes | Fortney, 2012  Yoon, 2016 | N/A |
| Stockdale, 2020 ^140^ | Evidence-based quality improvement (EBQI) is a multi-faceted implementation strategy that is based on a clinical-researcher partnership. It promotes organizational change by fostering innovation and the spread of those innovations that are successful. | N/A | Yes | Yes | N/A |
| Stuart, 2018 ^141^ | N/A | Advanced education for nursing leaders is driven by a growing need for cost-effective implementation of evidence-based practices. | No | No | N/A |
| Swindle, 2017 ^142^ | N/A | Informed by the PARIHS framework, we will use a stakeholder-driven evidence-based quality improvement (EBQI) process to apply information gathered in qualitative interviews on barriers and facilitators to practice to inform the design of implementation strategies | No | No | Curran, 2008^35^ |
| Tabrizi, 2019 ^143^ | N/A | The purpose of this evidence-based quality improvement (QI) project was to implement an evidence-based guideline for the management of PONV in female adults undergoing GYN and/or breast surgery and anesthesia in an ambulatory setting, and to measure changes in the anesthesia providers' compliance with documentation of the Apfel risk score assessment as a means to guide PONV prophylaxis. | No | No | N/A |
| Tan, 2020 ^144^ | N/A | This evidence-based quality improvement (EBQI) project was conducted in two orthopedic wards of a tertiary hospital in Singapore. | No | No | N/A |
| Teeter, 2019 ^145^ | N/A | We will follow that by selecting a pharmacist-physician collaborative model and identify implementation strategies through an Evidence Based Quality Improvement (EBQI) process with key stakeholders. | No | No | Curran, 2008^35^ |
| Thibaut, 2017 ^146^ | N/A (EBQI stands for Ecosystem Based Quality Index) |  |  |  |  |
| Ting, 2013 ^147^ | N/A | A multidisciplinary evidence-based quality improvement collaborative resulted in a significant reduction in the CLABSI rate. | No | No | N/A |
| Titsworth, 2012 ^148^ | N/A | To date, there has been a shortage of evidence-based quality improvement initiatives that have shown positive outcomes in the neurosurgical patient population. | No | No | N/A |
| Tunkel, 2020 ^149^ | N/A | This guideline is intended to focus on evidence-based quality improvement opportunities judged most important by the guideline development group. | No | No | N/A |
| Uhrig-Hitchcock,2013 ^150^ | N/A | "The Interventions to Reduce Acute Care Transfers (INTERACTII) program is an evidence-based quality improvement that can reduce hospital transfers from nursing facilities, especially when fully implemented." | No | No | N/A |
| Unger-Ullmann, 2018 ^151^ | N/A | Not relevant to medicine; "This report describes the opportunities, challenges and limits of evidence-based quality improvement in university language teaching" | No | No | N/A |
| Unroe, 2015 ^152^ | N/A | This clinical demonstration project, funded by the Centers for Medicare and Medicaid Services Innovations Center, places a registered nurse in each nursing facility to implement an evidence?based quality improvement program with clinical support from nurse practitioners | No | No | N/A |
| Unutzer, 2006 ^153^ | N/A | Successful implementation of evidence-based quality improvement can be strengthened by increasing the accountability of health care providers through performance measurement. | No | Yes | N/A |
| Volpe, 2011 ^154^ | N/A | Discusses "Evidence-based practice change" but no direct mention of EBQI | No | No | N/A |
| Walker, 2019 ^155^ | N/A | "To institutionally implement an EBQI initiative to standardize and optimize pain treatment following neurosurgical procedures." | No | No | N/A |
| Walker-Smith, 2020 ^156^ | N/A | The purpose of this paper was to design an evidence-based quality improvement project to improve the screening mammogram completion rates by implementing education and a clinical referral tool in a primary care clinic | No | No | N/A |
| Warren, 2019 ^157^ | N/A | The purpose of this evidence-based quality improvement (QI) project was to implement an oral care protocol in the adult in-patient care areas of a level 1 trauma hospital and to evaluate its impact on the incidence of hospital-acquired pneumonia (HAP). | No | No | N/A |
| Weddle, 2013 ^158^ | N/A | A project team at the ASC conducted an evidence-based, quality improvement project to improve the effectiveness and efficiency of mydriasis. | No | No | N/A |
| Weiner, 2015 ^159^ | N/A | The objective of Heart Health NOW (HHN) is to determine if primary care practice support—a comprehensive evidence-based quality improvement strategy involving practice facilitation, academic detailing, technology support, and regional learning collaboratives—accelerates widespread dissemination and implementation of evidence-based guidelines for cardiovascular disease (CVD) prevention in small- to medium-sized primary care practices and, additionally, increases practices’ capacity to incorporate other evidence-based clinical guidelines in the future. | No | No | N/A |
| Welch, 2014 ^160^ | N/A | This evidence-based quality improvement project used an online approach to introduce continuing education regarding brief motivational interviewing within the constraints of the inpatient setting. | No | No | N/A |
| Whitfield, 2013 ^161^ | N/A | "Lewin's Change Theory guided the implementation of an EBQI project to improve the efficiency of the triage process." | No | No | N/A |
| Whitten, 2013 ^162^ | N/A | To enhance outcomes of patients with chronic noncancer pain treated with opioids in a primary care setting by implementing an evidence based quality improvement project. | Yes | No | N/A |
| Wilson, 2019 ^163^ | N/A | This article proposes an evidence-based quality improvement intervention that can be adopted by paramedics at individual, service-wide, or national levels to promote smoking cessation | No | No | None |
| Wong, 2019 ^164^ | N/A | The objective of this evidence-based quality improvement project was to improve clinical nursing handovers between registered nurses. | No | No | N/A |
| Wood, 2014 ^165^ | N/A | This evidence-based quality improvement project describes how a mobility program, devised for and put to use on a general medical unit in a large Midwestern academic health care system, improved patient outcomes. | No | No | N/A |
| Yano, 2008 ^166^ | Evidence‐based quality improvement (EBQI) methods are based on the premise that practices will have greater success in achieving true improvements through organizational change using prior evidence from the literature as a guide for their activities. | N/A | Yes | Yes | Shojania, 2005 |
| Yano, 2016 ^7^ | EBQI is a systematic approach to developing a multilevel research-clinical partnership that engages senior organizational leaders and local quality improvement (QI) teams in adapting and implementing new care models in the context of prior evidence and local practice conditions, with researchers providing technical support, formative feedback, and practice facilitation -EBQI also uses team-based CQI methods to help teams structure their aims and measures and conduct plan-do-study-act (PDSA) cycles, in addition to convening topic-focused workgroups with research/clinical expertise with periodic across-site meetings for training and sharing data and lessons learned.EBQI’s value-added contribution is an emphasis on (a) applying objective evidence, with (b) theory review and synthesis integrated into aspects of innovation design and implementation, (c) valid and reliable measurement, and (d) formal measurement feedback to stakeholders at all levels | N/A | Yes | Yano, 2008; Fox, 2016 | N/A |
| Yoon, 2016 ^8^ | EBQI aims to increase the rapidity and completeness of evidence-based care model implementation by aligning interdisciplinary QI in practices with the broader goals of leadership at national and regional levels. | N/A | Yes | Fortney, 2012 Yano, 2008 | N/A |
| Young, 2018 ^167^ | N/A | We designed and piloted an evidence-based quality-improvement project in four urgent care clinics. | No | No | N/A |
| Zabari, 2006 ^168^ | N/A | Five NICUs from the Vermont Oxford Network’s Evidence-Based Quality Improvement Collaborative in Neonatal and Perinatal Medicine tested potentially better practices. | No | No | N/A |
| Zeeman, 2019 ^169^ | N/A | In an era of medical education reform and increasing accountability at all levels of higher education, there is a need to understand how the time in which students engage in academic activities can inform evidence?based quality improvement of the curriculum. | No | No | N/A |
| Zeyzus-Johns, 2019 ^170^ | N/A | CMQCC has taken a 3-pronged approach to reduce maternal deaths and increase quality of maternity care delivered in the state of California: (1) improved maternal data tracking through the Maternal Data Center, (2) the creation of a statewide Pregnancy-Associated Mortality Review, and (3) implementation of evidence-based quality improvement initiatives. | No | No | N/A |
| Zhang, 2009 ^171^ | N/A | Evidence-based quality improvement in nursing homes relies heavily on administrative data | No | No | N/A |

Supplemental Digital Content Figure 1: Frequency of EBQI Publications


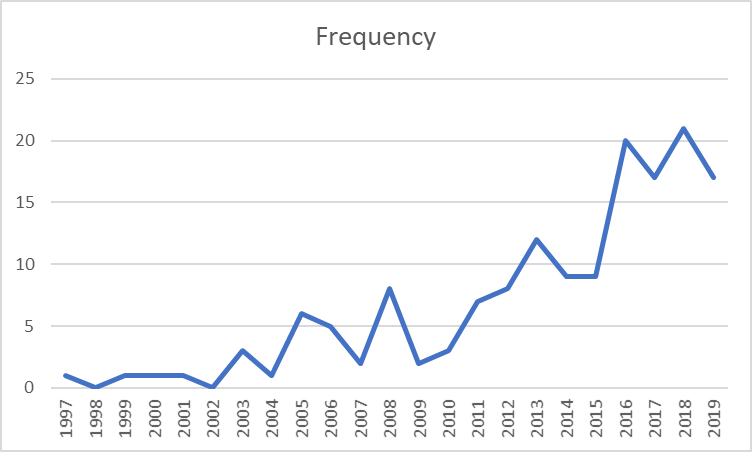


Supplemental Digital Content Table 2: Evidence Table EBQI and EBQI-Compatible Intervention Evaluations

| **Author, Date**  **Reference to “EBQI”** | **Topic** | **Outcome measures** | **Definition of evidence** | **Engagement - composition of initiative team** | | **Quality Improvement intervention** |
| --- | --- | --- | --- | --- | --- | --- |
| Badru, 2017 ^24^  EBQI | Managing PTSD | Nurse confidence | Literature review results | Healthcare providers, nurses, chief of quality management, mental health practitioners, Nurse Practice Council | Evidence-based newly developed clinical practice guideline based on social cognitive theory implemented | |
| Bennett, 2016 ^27^  EBQI | Lipid screening | Prevalence of screening | Literature review results, evidence-based guidelines | Doctor of nursing practitioner student, 3 nurse practitioners, 2 licensed practical nurses, lab assistant, medical director | Evidence-based guidelines | |
| Chaney, 2011 ^37^  EBQI | Depression care | Anti-depressant use | Results of modified Delphi panel | VA- regional directors, care managers, nursing leadership, primary care leadership | EBQI collaborative care model | |
| Cohen, 2013 ^40^  EBQI | Weight management for schizophrenic patients | Use of weight services | Literature review results; evidence-based psychosocial program | Clinicians and managers; clinical champions; patient and clinician education; social marketing; and evidence-based quality improvement teams | Patient facing kiosks, health-information technology | |
| Dumphy, 2016 ^46^  EBQI | Breastfeeding | Breastfeeding rate | Local data; clinical protocol | Staff and providers of rural pediatric primary care clinic | The Academy of Breastfeeding Medicine (ABM) Clinical Protocol | |
| Fortney, 2012 ^51^  EBQI | Depression in VA clinics | Depression treatment/referral | Results from randomized trials; anecdotal evidence from clinical experiences; guide for implementing collaborative-care management | Stakeholders from mental health, primary care, and the community based outpatient clinics, and the principal investigator | Collaborative-care management | |
| Fortney, 2013 ^52^  EBQI | Depression care | Treatment response rates (anti-depressants) | VA data; guideline-concordant care | Implementation team included one on site telephone depression nurse care manager and supervising telepsychiatrist | Collaborative care management | |
| Fox, 2016 ^5^  EBQI | Culturally sensitive healthcare/cultural competence training | Gender sensitivity and knowledge | Expert panel discussion and results of engaging local context | 4 geographically diverse VA facilities, multi-disciplinary teams | EBQI vs standard web-based implementation | |
| Gadbois, 2016 ^53^  EBQI | Health promotion/disparities in opioid treatment | Comprehensive health assessment, attention to nursing coordination | Literature review results; evidence-based tool | Advanced practice public health nurse, co-investigators, a PhD faculty mentor, agency Executive Director | Evidence-based comprehensive health assessment | |
| Gottlieb, 2018 ^57^  EBQI | Vaccinations | Vaccination rate | Pre-intervention medical record review; clinical guidelines | Physicians, project leaders, medical assistant, receptionist | Four Pillars Transformation Program | |
| Klause, 2020 ^78^  EBQI | Intimate partner violence prevention | Nurses addressing intimate partner violence during health visits | Chart review of patient data; microsystem analysis; evidence-based tool | Nurse practitioners | CUES tool, EHR template, nurse education | |
| Le Flore, 2017 ^83^  EBQI | Opioid prescribing | Opioid prescriptions | Literature review results, chart audits, peer review | Doctor of nurse practitioner student, risk manager, QI manager | CDC opioid guidelines | |
| Meredith, 2018 ^15^  EBQI | Quality improvement | Staff morale and job satisfaction | Results of literature review | Regional and local health system leaders and 2 frontline primary care practices from each of 3 of 5 local medical center-based Veterans Health Administration healthcare systems in the region | EBQI-engaged practices vs non-EBQI-engaged practices | |
| Ong, 2017 ^108^  EBQI | Nurse engagement | Nurse engagement and turnover | Focus groups results | Registered nurses from different departments | Shared governance environment | |
| Rizzo, 2018 ^120^  EBQI | Diabetes care | Knowledge and HgA1c levels | Literature review results; secondary data analysis from clinic | Doctor of nursing practice student | Nurse led education sessions | |
| Rubenstein, 2006 ^121^  EBQI | Depression in primary care | Appropriate treatment, depression, functional status, and satisfaction | Clinical practice guidelines; expert panel results | Researchers, senior system leaders; each practice had their own team and staffing, QI facilitators | EBQI | |
| Rubenstein, 2010 ^122^  EBQI | Depression care | Patient adherence and depression outcomes | Results of modified Delphi panel | Researchers, clinical and administrative leaders; participants in panels included regional administrative, mental health, primary care, nursing, quality improvement leaders, selected local primary care practice mental health and primary care leaders | Translating Initiatives in Depression into Effective Solution (TIDES) | |
| Sherman, 2004 ^130^  EBQI | Depression care | Physician recognition of depression in patients | AHRQ depression guidelines; panel of top administrators and service chiefs to arrive at a local consensus of priorities for managing depression | 2 primary care physicians, a consultation and liaison psychiatrist, a nurse practitioner, the administrative team manager, and a research assistant | Evidence-based mental health awareness project (using AHRQ guidelines) | |
| Starkey, 2016 ^134^  EBQI | Depression care | Physician practice pattern | Survey of physician practice and patient chart audit; evidence-based educational module | American College of Physicians staff, and 2 faculty members | Online educational module supplemented by national expert conference call coaching | |
| Walker, 2019 ^155^  EBQI | Pain control and reduce opioid consumption after surgery | Pain scores, opioid consumption, hospital stay length | Literature search results | Neurosurgery providers, pharmacists, pain management  specialists, and anesthesiologists | Locally generated pain-management protocol | |
| Walker-Smith, 2020 ^156^ Waker-Smith, 2018 ^172^  EBQI | Mammogram screening | Mammogram screening initiation | Literature search results, local data, evidence-based screening tools | Administrators, health providers, nurses, and community health workers | Education; Breast Cancer Risk Assessment Tool; National Health Interview Survey | |
| Whitten, 2013 ^162^  EBQI | Chronic pain treatment | Patient mood and function status, patient perception of treatment benefit | Literature search results and published clinical practice guidelines | Trained psychotherapist and a primary care nurse practitioner | Cognitive-behavioral therapy program | |
| Yano, 2008 ^166^  EBQI | Smoking cessation | Attendance at smoking clinics, smoking cessation | US Public Health Service smoking cessation guidelines; structured evidence review results; expert panel results | Site leaders, multi-disciplinary teams, research assistance | EBQI group-randomized trial design | |
| Yoon, 2016 ^8^  EBQI | Health care utilization | Mortality | Evidence review results; improvement proposals | Interdisciplinary regional leaders, local quality councils, veteran representatives, steering committee to review proposals | EBQI vs standard PACT implementation | |
| Young, 2018 ^167^  EBQI | Opioid prescribing | Rate of opioid prescribing | Local data; CDC guideline | 4 urgent care centers, no further details | Opioid-prescribing guideline | |
| Barclay, 2019 ^173^ | Alcohol use screening | Percent patients screened; facilitators and barriers encountered at clinic | National recommendations; findings from a systematic review; process map | Project lead (primary care clinician and researcher), clinic medical director, project coordinator, clinic project assistant, nurse manager, social workers/counselors, patient/family advisor | | Screening questions recommended by NIAAA |
| Bowen, 2020 ^174^ | Depression care | Depression reduction/remission; provider satisfaction | Recommendation from the Indian Health Service; evidence from Collaborative Care Management RCTs | 3 clinics; primary care provider, psychiatric consultant, behavioral health manager | | SIIF-CoCM (Social Innovation Fund Collaborative Care Management) |
| Breaux, Shropshire, 2017 ^175^ | Hypertension control | Controlled blood pressure | Local data, literature search results, SWOT (Strengths, Weaknesses, Opportunities, Threats) analysis | Not specified beyond 'interprofessional team'; a QI facilitator (VA Quality Scholar), and an MD | | Hypertension bundle intervention-- use of electronic medical record, non-traditional clinic visits, medication adherence assessment, reminders |
| Brodie, 2018 ^176^ | Vaccinations | Vaccination rate, missed vaccine opportunity rate | Local data; review of literature | Data scientist, resident physician, physician clinical champion, medical director, medical assistant champion | | Education, weekly reminders |
| Burge, 2019 ^177^ | Depression screening | Screening rates | Chart review results | Advanced practice nursing, a physician, Director of Informatics, Director of Nursing, Chief Executive Officer | | Biphasic secondary depression screening process |
| Buschkoetter, 2019 ^178^ | Diabetes care | Percent of patients screened | Retrospective chart review; evidence-based screening guidelines | 4 rural health centers; team included physician, nurse practitioners | | Comprehensive diabetic foot exam |
| Camp, 2017 ^179^ | Childhood obesity | Provider recognition of overweight/obese, assessment of health habits, counseling, and goal setting | Local data, clinical guidelines | Not specified | | Childhood Healthy Behaviors Intervention (CHBI) |
| Campbell, 2017 ^180^ | Autism screening | Accurate documentation, appropriate action | Evidence-based screening tool | Not specifed | | Digital version of Modified Checklist for Autism in Toddlers with follow-up |
| Colborn, 2019 ^181^ | Asthma care | Process of care, medication management | Clinical guidelines | Local stakeholders, investigators from National Jewish Health, nurse practice coach, asthma champion | | Colorado Asthma Toolkit Program (CATP) |
| Daaleman, 2018 ^182^ | Academic primary care | Quality of care, patient appointment cycle time, practice productivity | Evidence review results | Content experts, clinical and administrative leaders, champions | | Lean: a group of organizational philosophies, methods, and tools which create value for patients by improving quality and efficiency of care |
| Fabre, 2020 ^183^ | Electronic referrals | Referral rates | Care coordination model developed by the MacColl Institute for Healthcare Innovation (MIHI) | Chief health information officer, director of quality, referral coordinator | | Care coordination model |
| Fisher-Borne, 2018 ^184^ | Vaccinations | Vaccine series initiation and completion | Clinical guidelines; site self assessment | 30 federally qualified health center systems receiving 3 different interventions; QI teams | | HPV Vaccinate Adolescents against Cancers Program |
| Fortney, 2018 ^185^ | Mood disorders | Depression and bipolar symptoms, screening, engagement, correct prescriptions | Needs assessment results; clinical guidelines | Clinician, 2 patients, 2 researchers, 2 clinical content experts | | Screening, consultation, prescribing guidelines, on-line therapy and peer support |
| Garza, 2017 ^186^ | Cardiovascular disease | Assessment of cardiovascular disease risk score, diet/exercise counseling, statin therapy | Local data; literature review; clinical guidelines | Multidisciplinary team | | Education, electronic alert, and QI sessions |
| Gold, 2017 ^187^ Gold, 2012 ^188^ | Cardiovascular care | Recommended prescribing | Internal review, clinical guidelines | Researchers, EHR programers, community health center staff and providers | | ALL Initiative (guideline-based cardiovascular guidelines for prescribing) |
| Green, 2017 ^189^ | Cancer screening | Colorectal cancer screening rate | Local data, results of ongoing RCT | Physician, clinical operations manager, colorectal screening expert | | Mailing fecal immunochemical test kits to patients |
| Hanlin, 2018 ^190^ | Hypertension | Blood pressure control | Clinical guidelines | The American Medical Association partnered with John Hopkins Medicine to develop the framework | | Measure Accurately, Act Rapidly, and Partner With Patients (MAP) |
| Hawk, 2017 ^191^ | Vaccinations | Vaccination rate | Evidence-based intervention (4 Pillars Practice Transformation Program) | Study investigators, immunization champion, physicians, medical assistants, practice managers | | 4 Pillars Practice Transformation Program |
| Jonas, 2017 ^192^ | Alcohol use screening | Percent of patients receiving screening and interventions | Clinical guidelines, evidence-based counseling (motivational interviewing) | Not specified | | Screening instruments, evidence-based counseling intervention, shared decision making, support materials, and motivational interviewing training |
| Knierim, 2019 ^193^ | Cardiovascular health | Time to report electronic clinical quality measures | Health Information Technology for Economic and Clinical Health Act; AHRQ-selected specifications | Research team, practice facilitator, clinical health information technology advisor | | EvidenceNOW Southwest |
| Lu, 2019 ^194^ | Psychiatric consultation via the electronic health record | Provider's management of issues, knowledge, and patient care | Local needs assessment | Family medicine/psychiatric residents, integrated attending psychiatrist | | Psychiatric E-Consult model |
| Makelarski, 2019 ^195^ | Cardiovascular disease | Appropriateness, feasibility, adoption | Evidence-based QI strategies (Healthy Hearts in the Heartland; H3) | Facilitator, practice champions (providers, clinic managers, medical assistants) | | Community Rx-H3: Community resource referral system |
| Minsky, 2017 ^196^ | Endocrine e-consults | Primary care provider satisfaction | Local needs assessment | Clinical champion, primary care providers | | Endocrine eConsult service |
| Modica, 2019 ^197^ | Cancer screening | Eligible patients screened | The Community Prevention Services Task Force recommendations; pre-intervention local data | 4 health centers; multidisciplinary teams at a minimum including nurse care manager, provider, QI, health information technology expert, finance representative | | Value Transformation Network, Learning Community Model |
| Nagykaldi, 2017 ^198^ | Preventive care | Delivery of preventive services | Institute of Medicine report on collaborative models | Academic study team, full-time wellness coordinator, practice enhancement assistant, local non-profit County Health Improvement Organization, | | Community-based patient outreach model |
| Nowalk, 2017 ^199^ | Vaccinations | Vaccination rate | Evidence-based key domains (4 Pillars Transformation Program) | Study investigators, immunization champion, physicians, medical assistants, practice managers | | 4 Pillars Practice Transformation Program |
| Ober, 2017 ^200^ | Substance abuse treatment | Organizational readiness: acceptability, feasibility, intent to adopt evidence-based treatments | Focus groups and team meetings | Research team (PI and investigator), clinic medical director, behavioral health director, clinic leaders | | Organizational readiness intervention |
| Quanbeck, 2018 ^201^ | Opioid prescribing | Morphine-equivalent daily dose, urine drug tests, treatment agreements, opioid-benzodiazepine co-prescribing | Clinical guidelines, delphi process | Physician consultant, systems consultant, prescriber, researcher, facilitator, experts on implementation research | | Systems consultation |
| Regan, 2017 ^202^ | Chronic kidney disease | Detection, evaluation, referral of patients | Literature review, clinical guidelines | Nurse practitioner (team leader), three directors of medical informatics, QI coordinator, information technology support, local nephrologist | | Evidence-based clinical algorithm, clinical decision support (CDS) tool |
| Richards, 2019 ^203^ | Substance use disorder screening | Proportion of patients diagnosed with and treated for substance use disorder | Screening guidelines and substance use disorder checklist | 3 large outpatient clinics; included site leaders, social workers, practice coaches, QI consultants, research staff | | Behavioral Health Integration Practice coaching, EHR decision support, performance feedback |
| Roderick, 2017 ^204^ | Behavioral health integration | Level of behavioral health integration | Baseline needs assessment | Practice facilitator, integrated BH provider, physician champion, nurse care manager, lead medical assistant, office manager and data support specialist. | | Behavioral health integration with a practice facilitator into patient-centered medical homes |
| Savas, 2019 ^205^ | Electronic Health Record improvement | Quality measure adherence scores | Literature review results, expert evidence, needs assessment | Project coordinators, stakeholders, providers, support staff, interoffice champion | | Information technology process improvement |
| Schaeffer, 2019 ^206^ | Depression screening | Screening, adherence to follow-up | Local data (gap analysis), evidence-based guidelines | Nurse midwife (project leader), clinic medical director, multidisciplinary team of clinical staff | | Screening tools, Option Grid, tracking log |
| Schiff, 2017 ^207^ | Ambulatory malpractice | Documentation of results, days between lab test date and completed action/treatment plan | Chart review | Frontline staff, senior leader, clinical champion, day-to-day champion | | Proactive Reduction of Outpatient Malpractice: Improving Safety, Efficacy and Satisfaction Project (PROMISES) |
| Schurman, 2017 ^208^ | Vaccinations | Use and satisfaction with pain prevention strategies, staff and caregiver attitudes | Clinical guidelines; local data | Pediatric psychologists with expertise in pain, certified  pain management nurse, nursing administrator, physician, and QI specialist | | Educational videos and modules, specific intervention strategies |
| Senger, 2018 ^209^ | Management of concussions | Provider knowledge and satisfaction, diagnosis and management of concussions | Literature review results, evidence-based practice guideline | Doctor of nursing practice, graduate student, family nurse practitioner, graduate school faculty member, graduate school faculty member, graduate school appointed faculty member | | Educational sessions and concussion toolkit |
| Shah, 2019 ^210^ | Electronic Health Record notifications | Number of daily EHR notifications | Local data, expert consensus, prior review of evidence | Chief Medical Officer, Chief Health Informatics Officer, or primary care leader | | Nationally developed toolkit |
| Sloand, 2019 ^211^ | Vaccinations | Vaccination rate | Health People 2020 endorsement | Clinic medical director, nurse practitioner | | mHealth text Messaging |
| van Eeghen, 2020 ^212^ | Opioid prescribing | Process measures, provider satisfaction, toolkit completion | Clinical guidelines, local data | Study authors, QI facilitator, and various teams (multiple clinics in QI intervention) included clinic providers and staff and a champion | | Opioid management toolkit |
| Weiner, 2017 ^213^ | Cancer screening | Percentage of eligible patients receiving cancer screening | Clinical guidelines, evidence-based toolkit, key informants | Practice facilitator, clinic staff, research staff | | Practice facilitation and evidence-informed toolkit |
| Williams, 2018 ^214^ | Anxiety treatment | Patient anxiety improvement | Local data; RCT data (CALM model) | Desk staff, nursing, primary care provider champion, social workers, psychologist, psychiatry, QI facilitator | | Coordinated Anxiety Learning and Management |
| Yusupov, 2019 ^215^ | Hypertension care | Patient education, physical activity, and weight reduction | Local data, clinical guidelines | Not specified other than electronic medical record support team | | Hypertension QI initiative- patient registry, peer education, and electronic health record improvements |

Notes: The table lists studies explicitly described as EBQI first, followed by EBQI-compatible studies in alphabetical order

Abbreviations: CDC Centers for Disease Control and Prevention, EBQI evidence-based quality improvement, EHR electronic health record, KQ key question, RCT randomized controlled trial, PI principle investigator, QI quality improvement

Supplemental Digital Content Table 3: EBQI Effectiveness

| **ID** | **Intervention** | **Number of participants** | **Outcome Measure**  **Follow up** | **Effect estimate and other results** |
| --- | --- | --- | --- | --- |
| Chaney, 2011 ^37^ Rubenstein, 2010 ^122^ | Depression care management protocol, followed by DCMs for 6 months, follow-up calls that included patient self-management support | Intervention N: 288  Control N: 258 | Antidepressants % of patients receiving appropriate antidepressant care (adequate dosage)  Follow up in months: 7 | RR: 1.37; CI 0.97, 1.94 |
| Cohen, 2013 ^40^ | Mental health; Patient-facing kiosks, continuous data feedback, clinical champions, education. | Intervention N: 281  Control N: 290 | Weight management visit Number of weight management per year  Follow up in months: 12 | SMD 2.84; CI 2.61, 3.07 |
| Dumphy, 2016 ^46^ | Increase breast-feeding initiation and continuation rates by using ABM's protocol | Intervention N: 45  Control N: 43 | Breast-feeding Breast feeding at 4 months  Follow up in months: 4 | RR: 1.53; CI 0.78, 2.99 |
| Fortney, 2013 ^52^ Fortney, 2012 ^51^ | Telephone depression nurse, supervising telepsychiatrist, VA Depression Case Finder, requesting consults and new anti-depressant medications for patients, referrals to care manager | Intervention N: 3848  Control N: 2394 | Anti-depressant medication Medication possession ratio of antidepressants  Follow up in months: 24 | SMD 0.18; CI 0.12, 0.23 |
| Fortney, 2018 ^185^ | Screening, consultation, prescribing guidelines, on-line therapy and peer support | Intervention N: 104  Control N: | Clinical response 50% reduction in depression symptoms  Follow up in months: 3 | N/A |
| Gottlieb, 2018 ^57^ | Immunization standing orders, vaccine needs screening, patient education, electronic medical record alerts, immunization champion, QI meetings | Intervention N: 182  Control N: 155 | Vaccination rate Pneumococcal polysaccharide vaccine 23 vaccine administered  Follow up in months: 2.5 | RR: 2.6; CI 1.93, 3.5 |
| Le Flore, 2017 ^83^ | 2016 CDC guidelines for opioid prescribing including-- patient risk assessment, pain contract, drug screens, CURES report, discussing expected duration of treatment and outcomes | Intervention N:  Control N: | Opioid prescribing Number of patients actively being prescribed opioid agonists  Follow up in months: 18 | 41% (p < .01) reduction in prescribing rates |
| Rizzo, 2018 ^120^ | Three 45 minutes sessions focusing on advanced technological training specific to Continuous Subcutaneous Insulin Infusion (CSII) therapy; development of self-management skills | Intervention N: 26  Control N: 34 | Glycated hemoglobin Overall HgA1c  Follow up in months: 3 | SMD -0.66; CI -1.19, -0.14 |
| Rubenstein, 2006 ^121^ Rubenstein, 2012 ^42^ | Identifying priorities, initiating EBQI process, and providing references and tools (depression toolkit), guideline-based goals, orientation sessions. Provider education and support, patient education, screening/detection, assessment, care management, collaboration with mental health specialists | Intervention N: 369  Control N: 198 | Appropriate depression treatment Appropriate treatment with antidepressants or psychotherapy, and patient depression education  Follow up in months: 12 | RR: 0.62; CI 0.42, 0.91 |
| Rubenstein, 2010 ^122^ | Depression care; collecting patient adherence to intervention and outcomes, patients are followed by a depression care manager | Intervention N: 178  Control N: 208 | Patient health PHQ-9  Follow up in months: 36 | SMD -2.26; CI -2.52, -2.01 |
| Walker-Smith, 2020 ^156^ Waker-Smith, 2018 ^172^ | Mammogram screening initiation , breast cancer screening tools | Intervention N:  Control N: | Screening rate Mammogram completion rate  Follow up in months: 3 | The 3-month total for mammogram completion rates increased by 7.21% |
| Whitten, 2013 ^162^ | 6 week group cognitive behavioral therapy facilitated by psychotherapist and primary care nurse practitioner | Intervention N: 22  Control N: 22 | Mood/depression Beck Depression Inventory II score  Follow up in months: 1.5 | SMD -0.74; CI -1.35, -0.13 |
| Yano, 2008 ^166^ | 30 minute didactic sessions, promoting detection of smokers through screening, clinical practice guidelines, referral to smoking cessation programs and counseling | Intervention N: 925  Control N: 1016 | Smoking cessation % of enrolled smokers who quit smoking  Follow up in months: 12 | RR: 1.06; CI 0.42, 2.68 |
| Yoon, 2016 ^8^ Rubenstein, 2014 ^123^ | Team-based care, non-visit based medication renewal, secure messaging, telephone management of access, telephone follow-ups. | Intervention N: 41111  Control N: 95745 | Mortality Mortality by date of death  Follow up in months: 48 | There was no independent effect of EBQI on mortality rate relative to comparison practices |

Notes: CI confidence interval, RR relative risk, SMD standardized mean differences

Supplemental Digital Content Figure: 2: Effect Size Estimates, Continuous Outcomes


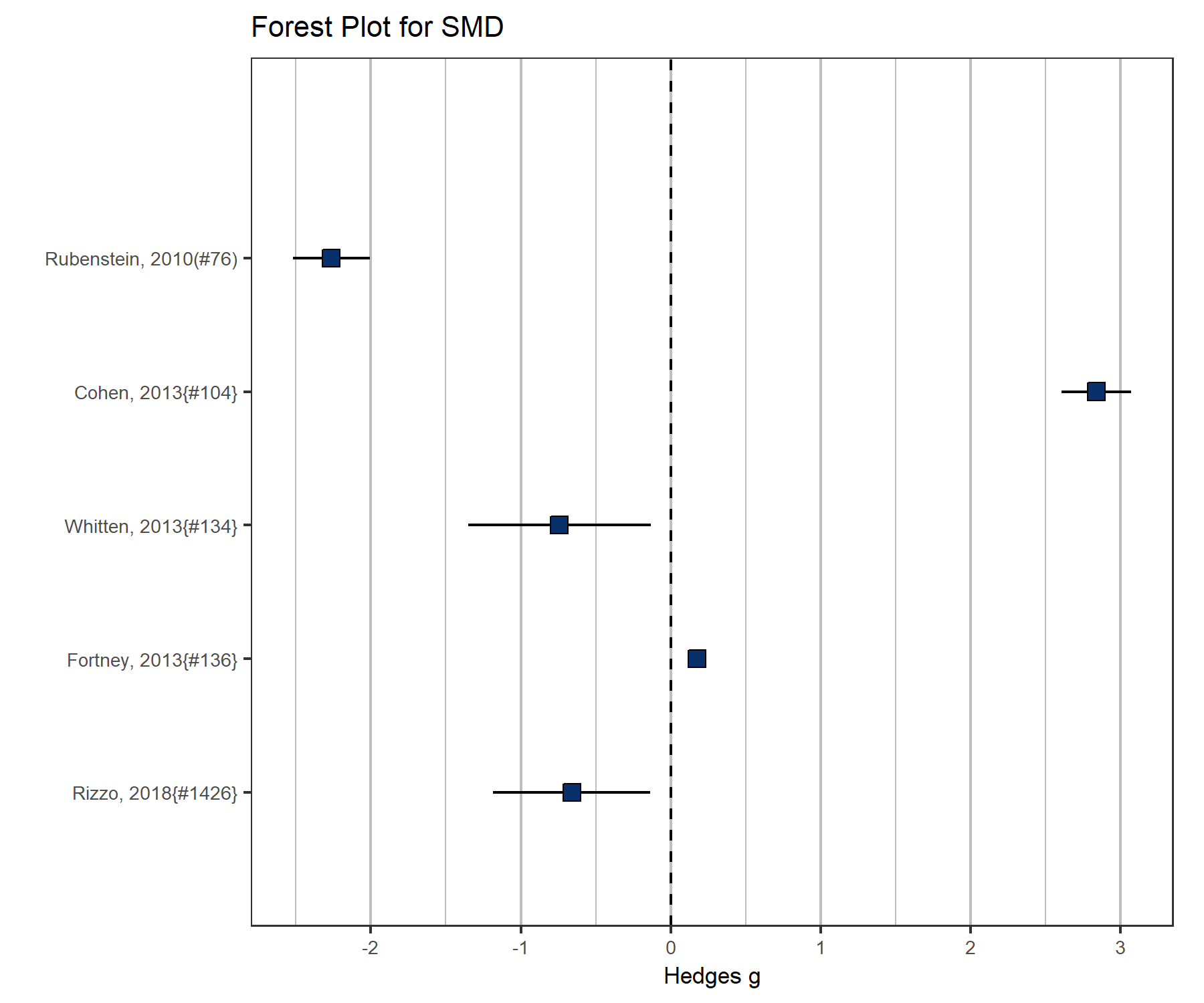


## References

1. Blakeman JR, Sarsfield K, Booker KJ. Nurses' Practices and Lead Selection in Monitoring for Myocardial Ischemia: An Evidence-Based Quality Improvement Project. *Dimensions of critical care nursing : DCCN.* 2015;34(4):189-195.

2. Melnyk BM, Buck J, Gallagher-Ford L. Transforming Quality Improvement Into Evidence-Based Quality Improvement: A Key Solution to Improve Healthcare Outcomes. *Worldviews on evidence-based nursing.* 2015;12(5):251-252.

3. Potier T, Tims E, Kilbride C, Rantell K. Evaluation of an evidence based quality improvement innovation for patients with musculoskeletal low back pain in an accident and emergency setting. *BMJ quality improvement reports.* 2015;4(1).

4. Bauer C, Magnan M, Laszewski P. Use of 4MAT Learning Theory to Promote Better Skin Care During Radiation Therapy: An Evidence-Based Quality Improvement Project. *Journal of wound, ostomy, and continence nursing : official publication of The Wound, Ostomy and Continence Nurses Society.* 2016;43(6):610-615.

5. Fox AB, Hamilton AB, Frayne SM, et al. Effectiveness of an Evidence-Based Quality Improvement Approach to Cultural Competence Training: The Veterans Affairs' "Caring for Women Veterans" Program. *The Journal of continuing education in the health professions.* 2016;36(2):96-103.

6. Huynh AK, Lee ML, Farmer MM, Rubenstein LV. Application of a nonrandomized stepped wedge design to evaluate an evidence-based quality improvement intervention: a proof of concept using simulated data on patient-centered medical homes. *BMC medical research methodology.* 2016;16(1):143.

7. Yano EM, Darling JE, Hamilton AB, et al. Cluster randomized trial of a multilevel evidence-based quality improvement approach to tailoring VA Patient Aligned Care Teams to the needs of women Veterans. *Implement Sci.* 2016;11(1):101.

8. Yoon J, Chow A, Rubenstein LV. Impact of Medical Home Implementation Through Evidence-based Quality Improvement on Utilization and Costs. *Medical care.* 2016;54(2):118-125.

9. Agee J. Reducing Chronic Obstructive Pulmonary Disease 30-Day Readmissions: A Nurse-Led Evidence-Based Quality Improvement Project. *The Journal of nursing administration.* 2017;47(1):35-40.

10. Bacidore V, Letizia M, Mitchel AM. Implementing Interprofessional Alcohol Screening, Brief Intervention, and Referral to Treatment in the Emergency Department: An Evidence-Based Quality Improvement Initiative. *Advanced emergency nursing journal.* 2017;39(3):199-216.

11. Hamilton AB, Brunner J, Cain C, et al. Engaging multilevel stakeholders in an implementation trial of evidence-based quality improvement in VA women's health primary care. *Translational behavioral medicine.* 2017;7(3):478-485.

12. Hamilton AB, Yano EM. The importance of symbolic and engaged participation in evidence-based quality improvement in a complex integrated healthcare system: response to "The science of stakeholder engagement in research". *Translational behavioral medicine.* 2017;7(3):492-494.

13. Khalil H. Evidence-based quality improvement. *International journal of evidence-based healthcare.* 2017;15(3):81.

14. Goldstein KM, Vogt D, Hamilton A, et al. Practice-based research networks add value to evidence-based quality improvement. *Healthcare (Amsterdam, Netherlands).* 2018;6(2):128-134.

15. Meredith LS, Batorsky B, Cefalu M, et al. Long-term impact of evidence-based quality improvement for facilitating medical home implementation on primary care health professional morale. *BMC family practice.* 2018;19(1):149.

16. Meyer MJ, Dzik WH, Levine WC. Reduction in Operating Room Plasma Waste After Evidence-Based Quality Improvement Initiative. *Anesthesia and analgesia.* 2018;126(5):1662-1665.

17. Soltis TM, Milner KA, Buonocore D. Transitions in Care From Acute Care Telemetry Unit to Home: An Evidence-Based Quality Improvement Project. *Critical care nurse.* 2018;38(5):77-80.

18. Stockdale SE, Zuchowski J, Rubenstein LV, et al. Fostering evidence-based quality improvement for patient-centered medical homes: Initiating local quality councils to transform primary care. *Health care management review.* 2018;43(2):168-180.

19. Gellert GA, Davenport CM, Minard CG, Castano C, Bruner K, Hobbs D. Reducing pediatric asthma hospital length of stay through evidence-based quality improvement and deployment of computerized provider order entry. *The Journal of asthma : official journal of the Association for the Care of Asthma.* 2019:1-13.

20. Radbron E, Wilson V, McCance T, Middleton R. The Use of Data Collected From mHealth Apps to Inform Evidence-Based Quality Improvement: An Integrative Review. *Worldviews on evidence-based nursing.* 2019;16(1):70-77.

21. Sarff L, O'Brien R. Evidence-Based Quality Improvement Training Programs: Building Staff Capability and Organizational Capacity. *Journal of nursing care quality.* 2019.

22. Ainsworth RM, Mog C, Summerlin-Long S. A Comprehensive Newborn Falls Initiative One Year Later. *JOGNN: Journal of Obstetric, Gynecologic & Neonatal Nursing.* 2014;43(Supp 1):S66-S66.

23. Arnold CS. Evidence-Based Quality Improvement Measures Contributed to Saving a Woman and Experiencing Amniotic Fluid Embolism and her Fetus in the Second Stage of Labor. *JOGNN: Journal of Obstetric, Gynecologic & Neonatal Nursing.* 2014;43(Supp 1):S91-S92.

24. Badru M. A Clinical Practice Guideline to Reduce Behavioral Outbursts in Veterans with Posttraumatic Stress Disorder. *Clinical Practice Guideline to Reduce Behavioral Outbursts in Veterans with Posttraumatic Stress Disorder.* 2017:1-1.

25. Bashford T. Quality improvement in low resource settings: an Ethiopian experience. *British journal of hospital medicine (London, England : 2005).* 2013;74(5):286-288.

26. Becker KD, Johnson S, Rucker D, Finnell DS. Dissemination of scholarship across eight cohorts of doctor of nursing practice graduates. *Journal of clinical nursing.* 2018;27(7-8):e1395-e1401.

27. Bennett JG. Implementing Lipid Screening Guidelines for Children in a Rural Health Clinic. *Implementing Lipid Screening Guidelines for Children in a Rural Health Clinic.* 2016:1-1.

28. Berman L, Vinocur CD. Improving quality on the pediatric surgery service: Missed opportunities and making it happen. *Seminars in pediatric surgery.* 2015;24(6):307-310.

29. Berta WB, Wagg A, Cranley L, et al. Sustainment, Sustainability, and Spread Study (SSaSSy): Protocol for a study of factors that contribute to the sustainment, sustainability, and spread of practice changes introduced through an evidence-based quality-improvement intervention in Canadian nursing homes. *Implementation Science.* 2019;14(1).

30. Bonner L, Felker B, Chaney E, et al. Advances in Patient Safety Suicide Risk Response: Enhancing Patient Safety Through Development of Effective Institutional Policies. In: Henriksen K, Battles JB, Marks ES, Lewin DI, eds. *Advances in Patient Safety: From Research to Implementation (Volume 3: Implementation Issues).* Rockville (MD): Agency for Healthcare Research and Quality (US); 2005.

31. Briscoe GT, Heerschap A, Kane CF, Quatrara BD. Using Post-Discharge Telephone Follow-Up by Nephrology Nurses to Reduce 30-Day Readmissions and Post-Discharge Complications for Adult Patients on Hemodialysis. *Nephrology nursing journal : journal of the American Nephrology Nurses' Association.* 2018;45(3):243-267.

32. Britto MT. Using evidence-based techniques to modify anemia screening practice. *The American journal of managed care.* 1997;3(10):1507-1509.

33. Brown BB, Haines M, Middleton S, et al. Development and validation of a survey to measure features of clinical networks. *BMC health services research.* 2016;16(1):531.

34. Burden JL, Parker JD, Williams DC, Lash SJ. Pre-Implementation Review of Contracts, Prompts, and Reinforcement in SUD Continuing Care. *The journal of behavioral health services & research.* 2017;44(1):135-148.

35. Curran GM, Mukherjee S, Allee E, Owen RR. A process for developing an implementation intervention: QUERI Series. *Implement Sci.* 2008;3:17.

36. Chaney E, Rabuck LG, Uman J, et al. Human subjects protection issues in QUERI implementation research: QUERI Series. *Implement Sci.* 2008;3:10.

37. Chaney EF, Rubenstein LV, Liu CF, et al. Implementing collaborative care for depression treatment in primary care: a cluster randomized evaluation of a quality improvement practice redesign. *Implement Sci.* 2011;6:121.

38. Chien L-Y. Evidence-Based Practice and Nursing Research. *Journal of Nursing Research (Lippincott Williams & Wilkins).* 2019;27(4):e29-e29.

39. Ciocson MAFR. A Nurse-Led Evidence-Based Quality Improvement Program on Childhood Obesity Prevention. *Nurse-Led Evidence-Based Quality Improvement Program on Childhood Obesity Prevention.* 2018:1-1.

40. Cohen AN, Chinman MJ, Hamilton AB, Whelan F, Young AS. Using patient-facing kiosks to support quality improvement at mental health clinics. *Medical care.* 2013;51(3 Suppl 1):S13-20.

41. Cordasco KM, Huynh AK, Zephyrin L, et al. Building capacity in VA to provide emergency gynecology services for women. *Medical care.* 2015;53(4 Suppl 1):S81-87.

42. Rubenstein LV, Parker LE, Meredith LS, et al. Understanding team-based quality improvement for depression in primary care. *Health services research.* 2002;37(4):1009-1029.

43. Curran GM, Pyne J, Fortney JC, et al. Development and implementation of collaborative care for depression in HIV clinics. *AIDS care.* 2011;23(12):1626-1636.

44. Dick AG, Pinder RJ, Lyle SA, Ember T, Mallinson C, Lucas J. Reducing Allogenic Blood Transfusion in Pediatric Scoliosis Surgery:: Reporting 15 Years of a Multidisciplinary, Evidence-Based Quality Improvement Project. *Global Spine Journal.* 2019;9(8):843-849.

45. Driscoll A, Tonkin A, Stewart A, et al. Development of an evidence-based scoring system (HF-IS) to assess the quality of heart failure programmes for patients postdischarge from hospital. *Journal of clinical nursing.* 2011;20(21-22):3011-3019.

46. Dumphy D, Thompson J, Clark M. A Breastfeeding Quality Improvement Project in Rural Primary Care. *Journal of human lactation : official journal of International Lactation Consultant Association.* 2016;32(4):633-641.

47. Epstein JN, Langberg JM, Lichtenstein PK, Kolb R, Simon JO. The myADHDportal.com Improvement Program: An innovative quality improvement intervention for improving the quality of ADHD care among community-based pediatricians. *Clinical practice in pediatric psychology.* 2013;1(1):55-67.

48. Estes TS, Short N, Bowser D, Boyle A. An evidence-based quality improvement perspective for a chronic obstructive pulmonary disease case-finding program. *Chronic respiratory disease.* 2014;11(3):131-138.

49. Faris N, Yu X, Sareen S, et al. Preoperative Evaluation of Lung Cancer in a Community Health Care Setting. *The Annals of thoracic surgery.* 2015;100(2):394-400.

50. Feng H, Li H, Xiao LD, et al. Aged care clinical mentoring model of change in nursing homes in China: study protocol for a cluster randomized controlled trial. *BMC health services research.* 2018;18(1):816.

51. Fortney J, Enderle M, McDougall S, et al. Implementation outcomes of evidence-based quality improvement for depression in VA community based outpatient clinics. *Implement Sci.* 2012;7:30.

52. Fortney JC, Enderle MA, Clothier JL, Otero JM, Williams JS, Pyne JM. Population level effectiveness of implementing collaborative care management for depression. *General hospital psychiatry.* 2013;35(5):455-460.

53. Gadbois C, Chin ED, Dalphonse L. Health promotion in an opioid treatment program an evidence-based nursing quality improvement project. *Journal of Addictions Nursing.* 2016;27(2):127-142.

54. Gammack JK, Philpot CD. Improving Quality of Care. In: *Pathy's Principles and Practice of Geriatric Medicine: Fifth Edition.* Vol 2.2012:1651-1672.

55. Giberson N. Implementation of A "Time Out" Tool to Decrease the Amount of Time Required from Anesthesia Arrival to Intubation for Emergent Intubations Located Outside of the Operating Room [OR] Supporting an Improvement in Patient Safety. *Implementation of A "Time Out" Tool to Decrease the Amount of Time Required from Anesthesia Arrival to Intubation for Emergent Intubations Located Outside of the Operating Room [OR] Supporting an Improvement in Patient Safety.* 2017:1-1.

56. Goodman MS, Sanders Thompson VL. The science of stakeholder engagement in research: classification, implementation, and evaluation. *Translational behavioral medicine.* 2017;7(3):486-491.

57. Gottlieb RP, Dols JD. Improving vaccination rates in adults with type 2 diabetes in a family practice setting through the use of evidence-based interventions. *Journal of Doctoral Nursing Practice.* 2018;11(2):151-159.

58. Goulding L, Parke H, Maharaj R, et al. Improving critical care discharge summaries: a collaborative quality improvement project using PDSA. *BMJ quality improvement reports.* 2015;4(1).

59. Griffin D, Hyrkas K. The Development and Implementation of an Obstetrical Triage Tool to Prioritize Patients and Track Process Times by Risk Categories. *JOGNN: Journal of Obstetric, Gynecologic & Neonatal Nursing.* 2014;43(Supp 1):S67-S67.

60. Grimshaw J, Eccles M, Thomas R, et al. Toward evidence-based quality improvement. Evidence (and its limitations) of the effectiveness of guideline dissemination and implementation strategies 1966-1998. *J Gen Intern Med.* 2006;21 Suppl 2:S14-20.

61. Güreşen A, Güreşen SO, Aktan Y. Combined synthetic and biotic indices of Posidonia oceanica to qualify the status of coastal ecosystems in the North Aegean. *Ecological Indicators.* 2020;113.

62. Gwatirisa JJ. Trimodal venous thromboembolism prophylaxis in total knee replacement: A quality improvement project for best care practices. *Journal of vascular nursing : official publication of the Society for Peripheral Vascular Nursing.* 2015;33(3):119-126.

63. Hameed SM, Brenneman FD, Ball CG, et al. General surgery 2.0: the emergence of acute care surgery in Canada. *Canadian journal of surgery Journal canadien de chirurgie.* 2010;53(2):79-83.

64. Hanna-Bull D. Preventing Heel Pressure Ulcers: Sustained Quality Improvement Initiative in a Canadian Acute Care Facility. *Journal of wound, ostomy, and continence nursing : official publication of The Wound, Ostomy and Continence Nurses Society.* 2016;43(2):129-132.

65. Harrison MS, Goldenberg RL. Making cesarean delivery SAFE in low- and middle-income countries. *Seminars in perinatology.* 2019;43(5):260-266.

66. Horbar JD. The Vermont Oxford Network: evidence-based quality improvement for neonatology. *Pediatrics.* 1999;103(1 Suppl E):350-359.

67. Horbar JD, Plsek PE, Schriefer JA, Leahy K. Evidence-based quality improvement in neonatal and perinatal medicine: The Neonatal Intensive Care Quality Improvement Collaborative experience. *Pediatrics.* 2006;118(SUPPL. 2):S57-S64.

68. Hunt JB, Curran G, Kramer T, et al. Partnership for implementation of evidence-based mental health practices in rural federally qualified health centers: theory and methods. *Progress in community health partnerships : research, education, and action.* 2012;6(3):389-398.

69. Hurwitz B, Brown J, Altmiller G. Improving Pediatric Temperature Measurement in the ED. *The American journal of nursing.* 2015;115(9):48-55.

70. Evidence-Based Quality Improvement Project. 2016/06//Jun2016 Supplement; Philadelphia, Pennsylvania.

71. Evidence-Based Quality Improvement Projects. *JOGNN: Journal of Obstetric, Gynecologic & Neonatal Nursing.* 2017;46:N.PAG.

72. Kasting ML, Christy SM, Sutton SK, et al. Florida physicians' reported use of AFIX-based strategies for human papillomavirus vaccination. *Preventive medicine.* 2018;116:143-149.

73. Katon WJ, Seelig M. Population-based care of depression: team care approaches to improving outcomes. *Journal of occupational and environmental medicine.* 2008;50(4):459-467.

74. Katona Z. Continuous quality improvement with the use of new, evidence based quality indicators in the primary health care: There is a real possibility to restrain the unnecessary raising of antibiotic resistance. *Orvosi Hetilap.* 2005;146(39):2005-2010.

75. Kilbride HW, Wirtschafter DD, Powers RJ, Sheehan MB. Implementation of evidence-based potentially better practices to decrease nosocomial infections. *Pediatrics.* 2003;111(4 Pt 2):e519-533.

76. Kilbride HW, Powers R, Wirtschafter DD, et al. Evaluation and development of potentially better practices to prevent neonatal nosocomial bacteremia. *Pediatrics.* 2003;111(4 Pt 2):e504-518.

77. Kitson A. Towards evidence-based quality improvement: perspectives from nursing practice. *Int J Qual Health Care.* 2000;12(6):459-464.

78. Klause KT, Dodds VA, Selleck C, Deupree JP. Addressing Intimate Partner Violence at a Safety-Net Clinic for Adults. *Journal for Nurse Practitioners.* 2020;16(2):154-157.

79. Kleczka B, Musiega A, Rabut G, et al. Rubber stamp templates for improving clinical documentation: A paper-based, m-Health approach for quality improvement in low-resource settings. *International journal of medical informatics.* 2018;114:121-129.

80. Koenig CJ, Abraham T, Zamora KA, et al. Pre-Implementation Strategies to Adapt and Implement a Veteran Peer Coaching Intervention to Improve Mental Health Treatment Engagement Among Rural Veterans. *The Journal of rural health : official journal of the American Rural Health Association and the National Rural Health Care Association.* 2016;32(4):418-428.

81. Kramer TL, Drummond KL, Curran GM, Fortney JC. Assessing Culture and Climate of Federally Qualified Health Centers: A Plan for Implementing Behavioral Health Interventions. *Journal of health care for the poor and underserved.* 2017;28(3):973-987.

82. Krug L, Machan MD, Villalba J. Changing Endotracheal Tube Taping Practice: An Evidence-based Practice Project. *AANA journal.* 2016;84(4):261-270.

83. Le Flore G. Applying Clinical Guidelines to Curtail Opioid Overprescribing in Primary Care. *Applying Clinical Guidelines to Curtail Opioid Overprescribing in Primary Care.* 2017:1-1.

84. Lennox RD, Mansfield AJ. A latent variable model of evidence-based quality improvement for substance abuse treatment. *The journal of behavioral health services & research.* 2001;28(2):164-176.

85. Levine R, Russo S, Su LM, Stringer T, Crispen P, Zlotecki RA. An Evaluation the Implementation of Evidence-Based Quality Improvement Metrics for Prostate Cancer Management in a Multidisciplinary Tertiary Care Setting. *International Journal of Radiation Oncology, Biology, Physics.* 2016;96:E275-E276.

86. Lipshutz AK, Fee C, Schell H, et al. Strategies for success: A PDSA analysis of three QI initiatives in critical care. *Jt Comm J Qual Patient Saf.* 2008;34(8):435-444.

87. Lockwood C, Stannard D, Jordan Z, Porritt K. The Joanna Briggs Institute clinical fellowship program: a gateway opportunity for evidence-based quality improvement and organizational culture change. *International journal of evidence-based healthcare.* 2020;18(1):1-4.

88. Loftus K, Tilley T, Hoffman J, Bradburn E, Harvey E. Use of Six Sigma strategies to pull the line on central line-associated bloodstream infections in a neurotrauma intensive care unit. *Journal of trauma nursing : the official journal of the Society of Trauma Nurses.* 2015;22(2):78-86.

89. Lozito M, Whiteman K, Swanson-Biearman B, Barkhymer M, Stephens K. Good Catch Campaign: Improving the Perioperative Culture of Safety. *AORN journal.* 2018;107(6):705-714.

90. Mainz J, Johnsen SP, Bartels PD. [Towards evidence-based quality improvement]. *Ugeskrift for laeger.* 2010;172(10):790-794.

91. Manheim S, Qian Hong L, Ekpo E. Improving Advance Directive Completion Rates for Patients Followed in the Melanoma Clinic Using the Advancing Research and Clinical Practice Through Close Collaboration Model(ARCC)...28th Annual Scientific Session, June 2-6, 2017, Baltimore, Maryland. *Nursing Research.* 2016;65(2):E32-E33.

92. Manns B, Braun T, Edwards A, et al. Identifying strategies to improve diabetes care in Alberta, Canada, using the knowledge-to-action cycle. *CMAJ open.* 2013;1(4):E142-150.

93. Mapes D. Nurses' impact on the choice and longevity of vascular access. *Nephrology nursing journal : journal of the American Nephrology Nurses' Association.* 2005;32(6):670-674.

94. Marang-van de Mheen PJ, van Bodegom-Vos L. Meta-analysis of the central line bundle for preventing catheter-related infections: a case study in appraising the evidence in quality improvement. *BMJ Qual Saf.* 2016;25(2):118-129.

95. Maru DS, Andrews J, Schwarz D, et al. Crossing the quality chasm in resource-limited settings. *Globalization and health.* 2012;8:41.

96. Marx M, Nitschke C, Nafula M, et al. If you can't measure it- you can't change it - a longitudinal study on improving quality of care in hospitals and health centers in rural Kenya. *BMC health services research.* 2018;18(1):246.

97. Intervention Research and Evidence-Based Quality Improvement: Designing, Conducting, Analyzing, and Funding, 2nd Edition. *Medicine & Science in Sports & Exercise.* 2020;52(1):267-267.

98. Matulewicz RS, Brennan J, Pruthi RS, Kundu SD, Gonzalez CM, Meeks JJ. Radical Cystectomy Perioperative Care Redesign. *Urology.* 2015;86(6):1076-1086.

99. McAllen ER, Jr., Stephens K, Swanson-Biearman B, Kerr K, Whiteman K. Moving shift report to the bedside: An evidence-based quality improvement project. *Online journal of issues in nursing.* 2018;23(2):1-1.

100. McCarthy C, Brennan JR, Brown L, et al. Use of a care bundle in the emergency department for acute exacerbations of chronic obstructive pulmonary disease: a feasibility study. *International journal of chronic obstructive pulmonary disease.* 2013;8:605-611.

101. McFrederick PC. *Participatory action research study to pursue excellence in diabetic management care*, Capella University; 2015.

102. Mendel P, Ngo VK, Dixon E, et al. Partnered evaluation of a community engagement intervention: use of a kickoff conference in a randomized trial for depression care improvement in underserved communities. *Ethnicity & disease.* 2011;21(3 Suppl 1):S1-78-88.

103. Montgomery A, Riley T, Tranter S, Manning V, Fernandez RS. Effect of an evidence based quality improvement framework on patient safety. *Australian Journal of Advanced Nursing.* 2018;35(4):6-16.

104. Moore CB, Hickey AH. Increasing Access to Auricular Acupuncture for Postoperative Nausea and Vomiting. *Journal of perianesthesia nursing : official journal of the American Society of PeriAnesthesia Nurses.* 2017;32(2):96-105.

105. Morrison K. Improving the care of stroke patients: using an evidence-based quality improvement initiative enhances outcomes for stroke patients. *American Nurse Today.* 2007;2(4):38-44.

106. Motz L. Reducing 30-day Readmissions Using the Rothman Index for Discharge Decision-Making...28th Annual Scientific Session, June 2-6, 2017, Baltimore, Maryland. *Nursing Research.* 2016;65(2):E42-E42.

107. Nichols R, Zawada E. A case study in therapeutic hypothermia treatment post-cardiac arrest in a 56-year-old male. *South Dakota medicine : the journal of the South Dakota State Medical Association.* 2008;61(10):371-373.

108. Ong A. Ripple effect: Shared governance and nurse engagement. *Nursing Management.* 2017;48(10):28-34.

109. Owen RR, Drummond KL, Viverito KM, et al. Monitoring and managing metabolic effects of antipsychotics: a cluster randomized trial of an intervention combining evidence-based quality improvement and external facilitation. *Implement Sci.* 2013;8:120.

110. Palm K, Apodaca A, Spencer D, et al. Evaluation of military trauma system practices related to complications after injury. *The journal of trauma and acute care surgery.* 2012;73(6 Suppl 5):S465-471.

111. Palm K, Apodaca A, Spencer D, et al. Evaluation of military trauma system practices related to damage-control resuscitation. *The journal of trauma and acute care surgery.* 2012;73(6 Suppl 5):S459-464.

112. Personnic S, Boudouresque CF, Astruch P, et al. An ecosystem-based approach to assess the status of a mediterranean ecosystem, the Posidonia oceanica seagrass meadow. *PLoS ONE.* 2014;9(6).

113. Post EP, Kilbourne AM, Bremer RW, Solano FX, Jr., Pincus HA, Reynolds CF, 3rd. Organizational factors and depression management in community-based primary care settings. *Implement Sci.* 2009;4:84.

114. Prince CR, Hines EJ, Chyou PH, Heegeman DJ. Finding the key to a better code: code team restructure to improve performance and outcomes. *Clinical medicine & research.* 2014;12(1-2):47-57.

115. Pulver LK, Wai A, Maxwell DJ, Robertson MB, Riddell S. Implementation and evaluation of a multisite drug usage evaluation program across Australian hospitals - a quality improvement initiative. *BMC health services research.* 2011;11:206.

116. Qureshi N, Weng S, Hex N. The role of cost-effectiveness analysis in the development of indicators to support incentive-based behaviour in primary care in England. *Journal of health services research & policy.* 2016;21(4):263-271.

117. Rahman N, Vinayakarao L, Pathak S, et al. Evaluation of training programme uptake in an attempt to reduce obstetric anal sphincter injuries: the SUPPORT programme. *International urogynecology journal.* 2017;28(3):403-407.

118. Rastorgueff PA, Bellan-Santini D, Bianchi CN, et al. An ecosystem-based approach to evaluate the ecological quality of Mediterranean undersea caves. *Ecological Indicators.* 2015;54:137-152.

119. Reiter KL, Kilpatrick KE, Greene SB, Lohr KN, Leatherman S. How to develop a business case for quality. *International Journal for Quality in Health Care.* 2007;19(1):50-55.

120. Rizzo KA. Effectiveness of Continuous Subcutaneous Insulin Infusion Therapy Education in a Clinic Setting. *Effectiveness Of Continuous Subcutaneous Insulin Infusion Therapy Education In A Clinic Setting.* 2018:1-1.

121. Rubenstein LV, Meredith LS, Parker LE, et al. Impacts of evidence-based quality improvement on depression in primary care: a randomized experiment. *J Gen Intern Med.* 2006;21(10):1027-1035.

122. Rubenstein LV, Chaney EF, Ober S, et al. Using evidence-based quality improvement methods for translating depression collaborative care research into practice. *Families, systems & health : the journal of collaborative family healthcare.* 2010;28(2):91-113.

123. Rubenstein LV, Stockdale SE, Sapir N, et al. A patient-centered primary care practice approach using evidence-based quality improvement: rationale, methods, and early assessment of implementation. *J Gen Intern Med.* 2014;29 Suppl 2:S589-597.

124. Salera-Vieira J. A Strategy to Review and Measure Retention of Key Concepts Related to Shoulder Dystocia Drills. *JOGNN: Journal of Obstetric, Gynecologic & Neonatal Nursing.* 2016;45:S40-S40.

125. Sangam SL. Quality improvement measures for early detection of severe intravenous infiltration in infants. *BMJ open quality.* 2019;8(2):e000407.

126. EB64 Enhancing Delirium Awareness and Recognition in the Medical Surgical Intensive Care Unit: An Evidence-Based Quality Improvement Initiative. *Critical care nurse.* 2012;32(2):e38-e38.

127. Saunders H. Translating knowledge into best practice care bundles: a pragmatic strategy for EBP implementation via moving postprocedural pain management nursing guidelines into clinical practice. *Journal of clinical nursing.* 2015;24(13-14):2035-2051.

128. Schultz AA, Gallant P. Evidence-based quality improvement project for determining appropriate discontinuation of peripheral intravenous cannulas. *Evidence-based nursing.* 2005;8(1):8.

129. Schwing L, Faulkner TD, Bucaro P, Herzing K, Meagher DP, Pence J. Trauma Team Activation: Accuracy of Triage When Minutes Count: A Synthesis of Literature and Performance Improvement Process. *Journal of trauma nursing : the official journal of the Society of Trauma Nurses.* 2019;26(4):208-214.

130. Sherman SE, Chapman A, Garcia D, Braslow JT. Improving recognition of depression in primary care: a study of evidence-based quality improvement. *Joint Commission journal on quality and safety.* 2004;30(2):80-88.

131. Shojania KG, Grimshaw JM. Evidence-based quality improvement: the state of the science. *Health Aff (Millwood).* 2005;24(1):138-150.

132. Smith WR. An explanation of theories that guide evidence-based interventions to improve quality. *Clinical Governance.* 2003;8(3):247-254.

133. Smith JL, Williams JW, Jr., Owen RR, Rubenstein LV, Chaney E. Developing a national dissemination plan for collaborative care for depression: QUERI Series. *Implement Sci.* 2008;3:59.

134. Starkey M, Wiest D, Qaseem A. Improving Depression Care Through an Online Learning Collaborative. *American journal of medical quality : the official journal of the American College of Medical Quality.* 2016;31(2):111-117.

135. Stearns E, Plymale MA, Davenport DL, et al. Early outcomes of an enhanced recovery protocol for open repair of ventral hernia. *Surgical endoscopy.* 2018;32(6):2914-2922.

136. Stetler CB, McQueen L, Demakis J, Mittman BS. An organizational framework and strategic implementation for system-level change to enhance research-based practice: QUERI Series. *Implement Sci.* 2008;3:30.

137. Stevans JM, Bise CG, McGee JC, Miller DL, Rockar P, Jr., Delitto A. Evidence-based practice implementation: case report of the evolution of a quality improvement program in a multicenter physical therapy organization. *Physical therapy.* 2015;95(4):588-599.

138. Stevens KR, Willard G. Evidence for evidence-based quality improvement (Re: Int J Evid Based Healthc 2010; 8(3): 109). *International journal of evidence-based healthcare.* 2011;9(1):67-68.

139. Stevens KR. The impact of evidence-based practice in nursing and the next big ideas. *Online journal of issues in nursing.* 2013;18(2):4.

140. Stockdale SE, Hamilton AB, Bergman AA, et al. Assessing fidelity to evidence-based quality improvement as an implementation strategy for patient-centered medical home transformation in the Veterans Health Administration. *Implement Sci.* 2020;15(1):18.

141. Stuart WP. Why Seek a Doctorate-Prepared Nurse to Join Your Team? *Health Care Manager.* 2018;37(3):220-224.

142. Swindle T, Johnson SL, Whiteside-Mansell L, Curran GM. A mixed methods protocol for developing and testing implementation strategies for evidence-based obesity prevention in childcare: a cluster randomized hybrid type III trial. *Implement Sci.* 2017;12(1):90.

143. Tabrizi S, Malhotra V, Turnbull ZA, Goode V. Implementation of Postoperative Nausea and Vomiting Guidelines for Female Adult Patients Undergoing Anesthesia During Gynecologic and Breast Surgery in an Ambulatory Setting. *Journal of perianesthesia nursing : official journal of the American Society of PeriAnesthesia Nurses.* 2019;34(4):851-860.

144. Tan AJQ, Rashasegaran A, Goh ML. Enhancing the verbal handover process for nurses in inpatient orthopedic wards: a best practice implementation project. *JBI database of systematic reviews and implementation reports.* 2020;18(1):200-211.

145. Teeter BS, Mosley C, Thomas JL, et al. Improving HPV vaccination using implementation strategies in community pharmacies: Pilot study protocol. *Research in social & administrative pharmacy : RSAP.* 2019.

146. Thibaut T, Blanfuné A, Boudouresque CF, et al. An ecosystem-based approach to assess the status of Mediterranean algae-dominated shallow rocky reefs. *Marine Pollution Bulletin.* 2017;117(1-2):311-329.

147. Ting JY, Goh VS, Osiovich H. Reduction of central line-associated bloodstream infection rates in a neonatal intensive care unit after implementation of a multidisciplinary evidence-based quality improvement collaborative: A four-year surveillance. *The Canadian journal of infectious diseases & medical microbiology = Journal canadien des maladies infectieuses et de la microbiologie medicale.* 2013;24(4):185-190.

148. Titsworth WL, Hester J, Correia T, et al. Reduction of catheter-associated urinary tract infections among patients in a neurological intensive care unit: a single institution's success. *Journal of neurosurgery.* 2012;116(4):911-920.

149. Tunkel DE, Anne S, Payne SC, et al. Clinical Practice Guideline: Nosebleed (Epistaxis). *Otolaryngology - Head and Neck Surgery (United States).* 2020;162(1_suppl):S1-S38.

150. Uhrig-Hitchcock LG, Granato A, Nguyen V, Holler R, Hochhalter A. Implementation of an Evidence-Based Quality Improvement Program towards Nursing Home Care...Long Term Care Medicine 2013 Conference. *Journal of the American Medical Directors Association.* 2013;14(3):B16-B16.

151. Unger-Ullmann D. Evidence-based Quality Improvement in University Language Didactics. *Language Learning in Higher Education.* 2018;8(1):173-185.

152. Unroe KT, Nazir A, Holtz LR, et al. The Optimizing Patient Transfers, Impacting Medical Quality, andImproving Symptoms:Transforming Institutional Care approach: preliminary data from the implementation of a Centers for Medicare and Medicaid Services nursing facility demonstration project. *J Am Geriatr Soc.* 2015;63(1):165-169.

153. Unutzer J, Schoenbaum M, Druss BG, Katon WJ. Transforming mental health care at the interface with general medicine: report for the presidents commission. *Psychiatric services (Washington, DC).* 2006;57(1):37-47.

154. Volpe AA. *Decreasing the Incidence of Inadvertent Perioperative Hypothermia: A Quality Improvement Study*, University of Connecticut; 2011.

155. Walker CT, Gullotti DM, Prendergast V, et al. Implementation of a Standardized Multimodal Postoperative Analgesia Protocol Improves Pain Control, Reduces Opioid Consumption, and Shortens Length of Hospital Stay After Posterior Lumbar Spinal Fusion. *Neurosurgery.* 2019.

156. Walker-Smith TL. *A Prospective Quality Improvement Project Using a Mammography Risk Assessment Tool to Increase Screening Mammogram Use with Low-income Hispanic Women: A Doctor of Nursing Practice Project Report.* Corpus Christi, Texas: Texas A&M University-Corpus Christi;2018.

157. Warren C, Medei MK, Wood B, Schutte D. A Nurse-Driven Oral Care Protocol to Reduce Hospital-Acquired Pneumonia. *The American journal of nursing.* 2019;119(2):44-51.

158. Weddle C, Thomas N, Dienemann J. Improved pupil dilation with medication-soaked pledget sponges. *AORN journal.* 2013;98(2):131-143.

159. Weiner BJ, Pignone MP, DuBard CA, et al. Advancing heart health in North Carolina primary care: the Heart Health NOW study protocol. *Implement Sci.* 2015;10:160.

160. Welch J. Building a foundation for brief motivational interviewing: communication to promote health literacy and behavior change. *Journal of continuing education in nursing.* 2014;45(12):566-572.

161. Whitfield CG. *Emergency department triage acuity ratings: Embedding ESI into the electronic medical record*, University of South Carolina; 2013.

162. Whitten SK, Stanik-Hutt J. Group cognitive behavioral therapy to improve the quality of care to opioid-treated patients with chronic noncancer pain: a practice improvement project. *Journal of the American Association of Nurse Practitioners.* 2013;25(7):368-376.

163. Wilson S, Hill L. What is the paramedic's role in smoking cessation? *Journal of Paramedic Practice.* 2019;11(3):100-105.

164. Wong X, Tung YJ, Peck SY, Goh ML. Clinical nursing handovers for continuity of safe patient care in adult surgical wards: a best practice implementation project. *JBI database of systematic reviews and implementation reports.* 2019;17(5):1003-1015.

165. Wood W, Tschannen D, Trotsky A, et al. A mobility program for an inpatient acute care medical unit. *The American journal of nursing.* 2014;114(10):34-40; quiz 41-32.

166. Yano EM, Rubenstein LV, Farmer MM, et al. Targeting primary care referrals to smoking cessation clinics does not improve quit rates: implementing evidence-based interventions into practice. *Health services research.* 2008;43(5 Pt 1):1637-1661.

167. Young LS, Crausman RS, Fulton JP. Suboptimal Opioid Prescribing: A Practice Change Project. *Rhode Island medical journal (2013).* 2018;101(2):41-44.

168. Zabari M, Suresh G, Tomlinson M, et al. Implementation and case-study results of potentially better practices for collaboration between obstetrics and neonatology to achieve improved perinatal outcomes. *Pediatrics.* 2006;118 Suppl 2:S153-158.

169. Zeeman JM, Kang I, Angelo TA. Assessing student academic time use: assumptions, predictions and realities. *Medical education.* 2019;53(3):285-295.

170. Zeyzus-Johns B, Stefanacci RG. Maternal Mortality Improvement Model with Broad Population Health Application. *Population health management.* 2019;22(1):9-11.

171. Zhang NJ, Paek SC, Wan TT. Reliability estimates of clinical measures between Minimum Data Set and Online Survey Certification and Reporting data of US nursing homes. *Medical care.* 2009;47(4):492-495.

172. Walker-Smith TL, Baldwin S. A Quality Improvement Initiative: Improving Mammogram Screening Rates Among Low-income Hispanic Women in Primary Care. *Journal of Doctoral Nursing Practice.* 2020;13(1):71-78.

173. Barclay C, Viswanathan M, Ratner S, Tompkins J, Jonas DE. Implementing Evidence-Based Screening and Counseling for Unhealthy Alcohol Use with Epic-Based Electronic Health Record Tools. *Jt Comm J Qual Patient Saf.* 2019;45(8):566-574.

174. Bowen DJ, Powers DM, Russo J, et al. Implementing collaborative care to reduce depression for rural native American/Alaska native people. *BMC health services research.* 2020;20(1):34.

175. Breaux-Shropshire TL, Huie R, Shropshire TS, et al. First Steps in Improving Blood Pressure Control Among Primary Care Hypertensive Veterans Utilizing Quality Improvement Tools. *Alabama Nurse.* 2017;44(3):19-22.

176. Brodie N, McPeak KE. Improving Human Papilloma Virus Vaccination Rates at an Urban Pediatric Primary Care Center. *Pediatr Qual Saf.* 2018;3(5):e098.

177. Burge SA, Powell W, Mazour L. A Quality Improvement Endeavor Improving Depression Screening for Rural Older Adults. *Online Journal of Rural Nursing & Health Care.* 2019;19(2):44-64.

178. Murphy Buschkoetter KL, Powell W, Mazour L. Implementation of a Comprehensive Diabetic Foot Exam Protocol in rural primary care. *Online Journal of Rural Nursing & Health Care.* 2019;19(1):43-63.

179. Camp NL, Robert RC, Nash JE, Lichtenstein CB, Dawes CS, Kelly KP. Modifying Provider Practice To Improve Assessment of Unhealthy Weight and Lifestyle in Young Children: Translating Evidence in a Quality Improvement Initiative for At-Risk Children. *Child Obes.* 2017;13(3):173-181.

180. Campbell K, Carpenter KLH, Espinosa S, et al. Use of a Digital Modified Checklist for Autism in Toddlers - Revised with Follow-up to Improve Quality of Screening for Autism. *J Pediatr.* 2017;183:133-139.e131.

181. Colborn KL, Helmkamp L, Bender BG, Kwan BM, Schilling LM, Sills MR. Colorado Asthma Toolkit Implementation Improves Some Process Measures of Asthma Care. *J Am Board Fam Med.* 2019;32(1):37-49.

182. Daaleman TP, Brock D, Gwynne M, et al. Implementing Lean in Academic Primary Care. *Qual Manag Health Care.* 2018;27(3):111-116.

183. Fabre JC, Andresen PA, Wiltz GM. Closing the Loop on Electronic Referrals: A Quality Improvement Initiative Using the Care Coordination Model. *J Ambul Care Manage.* 2020;43(1):71-80.

184. Fisher-Borne M, Preiss AJ, Black M, Roberts K, Saslow D. Early Outcomes of a Multilevel Human Papillomavirus Vaccination Pilot Intervention in Federally Qualified Health Centers. *Academic Pediatrics.* 2018;18(2):S79-S84.

185. Fortney JC, Pyne JM, Ward-Jones S, et al. Implementation of evidence-based practices for complex mood disorders in primary care safety net clinics. *Families, systems & health : the journal of collaborative family healthcare.* 2018;36(3):267-280.

186. Garza L, Dols J, Gillespie M. An initiative to improve primary prevention of cardiovascular disease in adults with type II diabetes based on the ACC/AHA (2013) and ADA (2016) guidelines. *Journal of the American Association of Nurse Practitioners.* 2017;29(10):606-611.

187. Gold R, Bunce A, Cowburn S, et al. Cardiovascular care guideline implementation in community health centers in Oregon: a mixed-methods analysis of real-world barriers and challenges. *BMC health services research.* 2017;17(1):253.

188. Gold R, Muench J, Hill C, et al. Collaborative development of a randomized study to adapt a diabetes quality improvement initiative for federally qualified health centers. *Journal of health care for the poor and underserved.* 2012;23(3 Suppl):236-246.

189. Green BB, Fuller S, Anderson ML, Mahoney C, Mendy P, Powell SL. A Quality Improvement Initiative to Increase Colorectal Cancer (CRC) Screening: Collaboration between a Primary Care Clinic and Research Team. *J Fam Med.* 2017;4(3).

190. Hanlin RB, Asif IM, Wozniak G, et al. Measure Accurately, Act Rapidly, and Partner With Patients (MAP) improves hypertension control in medically underserved patients: Care Coordination Institute and American Medical Association Hypertension Control Project Pilot Study results. *J Clin Hypertens (Greenwich).* 2018;20(1):79-87.

191. Hawk M, Nowalk MP, Moehling KK, et al. Using a Mixed Methods Approach to Examine Practice Characteristics Associated With Implementation of an Adult Immunization Intervention Using the 4 Pillars Practice Transformation Program. *J Healthc Qual.* 2017;39(3):153-167.

192. Jonas DE, Miller T, Ratner S, et al. Implementation and Quality Improvement of a Screening and Counseling Program for Unhealthy Alcohol Use in an Academic General Internal Medicine Practice. *J Healthc Qual.* 2017;39(1):15-27.

193. Knierim KE, Hall TL, Dickinson LM, et al. Primary Care Practices' Ability to Report Electronic Clinical Quality Measures in the EvidenceNOW Southwest Initiative to Improve Heart Health. *JAMA Netw Open.* 2019;2(8):e198569.

194. Implementation of psychiatric e-consultation in family medicine community health centers. *International Journal of Psychiatry in Medicine.* 2019;54(4/5):296-306.

195. Makelarski JA, DePumpo M, Boyd K, et al. Implementation of Systematic Community Resource Referrals at Small Primary Care Practices to Promote Cardiovascular Disease Self-Management. *J Healthc Qual.* 2019.

196. Minsky N, Tamler R. Endocrine eConsults improve access to care for the underserved. Paper presented at: ACM International Conference Proceeding Series2017.

197. Modica C, Lewis JH, Bay C. Colorectal Cancer: Applying the Value Transformation Framework to increase the percent of patients receiving screening in Federally Qualified Health Centers. *Prev Med Rep.* 2019;15:100894.

198. Nagykaldi ZJ, Scheid D, Zhao D, Mishra B, Greever-Rice T. An Innovative Community-based Model for Improving Preventive Care in Rural Counties. *J Am Board Fam Med.* 2017;30(5):583-591.

199. Nowalk MP, Moehling KK, Zhang S, Raviotta JM, Zimmerman RK, Lin CJ. Using the 4 Pillars to Increase Vaccination Among High-Risk Adults: Who Benefits? *American Journal of Managed Care.* 2017;23(11):651-655.

200. Ober AJ, Watkins KE, Hunter SB, et al. Assessing and improving organizational readiness to implement substance use disorder treatment in primary care: findings from the SUMMIT study. *BMC family practice.* 2017;18(1):107.

201. Quanbeck A, Brown RT, Zgierska AE, et al. A randomized matched-pairs study of feasibility, acceptability, and effectiveness of systems consultation: a novel implementation strategy for adopting clinical guidelines for Opioid prescribing in primary care. *Implement Sci.* 2018;13(1):21.

202. Regan ME. Implementing an evidence-based clinical decision support tool to improve the detection, evaluation, and referral patterns of adult chronic kidney disease patients in primary care. *Journal of the American Association of Nurse Practitioners.* 2017;29(12):741-753.

203. Richards JE, Bobb JF, Lee AK, et al. Integration of screening, assessment, and treatment for cannabis and other drug use disorders in primary care: An evaluation in three pilot sites. *Drug Alcohol Depend.* 2019;201:134-141.

204. Roderick SS, Burdette N, Hurwitz D, Yeracaris P. Integrated behavioral health practice facilitation in patient centered medical homes: A promising application. *Families, systems & health : the journal of collaborative family healthcare.* 2017;35(2):227-237.

205. Savas A, Smith E, Hay B. EHR quality indicator tracking: A process improvement pilot project to meet MACRA requirements. *Nurse Practitioner.* 2019;44(4):30-39.

206. Schaeffer AM, Jolles D. Not Missing the Opportunity: Improving Depression Screening and Follow-Up in a Multicultural Community. *Jt Comm J Qual Patient Saf.* 2019;45(1):31-39.

207. Schiff GD, Reyes Nieva H, Griswold P, et al. Randomized Trial of Reducing Ambulatory Malpractice and Safety Risk: Results of the Massachusetts PROMISES Project. *Medical care.* 2017;55(8):797-805.

208. Schurman JV, Deacy AD, Johnson RJ, et al. Using quality improvement methods to increase use of pain prevention strategies for childhood vaccination. *World J Clin Pediatr.* 2017;6(1):81-88.

209. Senger JJ. A Concussion Toolkit Educational Session: Promoting Evidence-based Management of Youth Concussion in a Rural Primary Care Setting. *Concussion Toolkit Educational Session: Promoting Evidence-Based Management Of Youth Concussion In A Rural Primary Care Setting.* 2018:1-1.

210. Shah T, Patel-Teague S, Kroupa L, Meyer AND, Singh H. Impact of a national QI programme on reducing electronic health record notifications to clinicians. *BMJ Qual Saf.* 2019;28(1):10-14.

211. Sloand E, Vangraafeiland B, Holm A, MacQueen A, Polk S. Text Message Quality Improvement Project for Influenza Vaccine in a Low-Resource Largely Latino Pediatric Population. *Journal for Healthcare Quality.* 2019;41(6):362-368.

212. van Eeghen C, Kennedy AG, Pasanen ME, MacLean CD. A New Quality Improvement Toolkit to Improve Opioid Prescribing in Primary Care. *J Am Board Fam Med.* 2020;33(1):17-26.

213. Weiner BJ, Rohweder CL, Scott JE, et al. Using Practice Facilitation to Increase Rates of Colorectal Cancer Screening in Community Health Centers, North Carolina, 2012-2013: Feasibility, Facilitators, and Barriers. *Prev Chronic Dis.* 2017;14:E66.

214. Williams MD, Sawchuk CN, Shippee ND, et al. A quality improvement project aimed at adapting primary care to ensure the delivery of evidence-based psychotherapy for adult anxiety. *BMJ open quality.* 2018;7(1):e000066.

## 215. Yusupov E, Krishnamachari B, Rand S, Abdalla M, Zwibel H. Quality of hypertension care: An improvement initiative in two outpatient health care centers. *J Eval Clin Pract.* 2019;25(3):463-468.Excluded publications with reasons for exclusion

Note: *Intervention* indicates the study did not evaluate a quality improvement initiative, *Duplicate* indicates the publication is an exact duplicate of an existing records, *Setting* indicates the study did not take place in primary care, *Study design* indicates there was no comparative data, and *Outcome* indicates the study did not report outcomes.

1. Project MATCH (Matching Alcoholism Treatment to Client Heterogeneity): rationale and methods for a multisite clinical trial matching patients to alcoholism treatment. Alcohol Clin Exp Res. 1993 Dec;17(6):1130-45. doi: 10.1111/j.1530-0277.1993.tb05219.x. PMID: 8116822. *Intervention*

2. Matching Alcoholism Treatments to Client Heterogeneity: Project MATCH posttreatment drinking outcomes. J Stud Alcohol. 1997 Jan;58(1):7-29. PMID: 8979210. *Intervention*

3. Evidence-Based Quality Improvement Training Programs: Building Staff Capability and Organizational Capacity. Journal of nursing care quality. 2020;35(2):E27-E8. doi: 10.1097/NCQ.0000000000000481. *Duplicate*

4. Acharya S, Philcox AN, Parsons M, et al. Hunter and New England Diabetes Alliance: innovative and integrated diabetes care delivery in general practice. Aust J Prim Health. 2019 Jun 21. doi: 10.1071/py18179. PMID: 31221243. *Setting*

5. Addington DE, McKenzie E, Norman R, et al. Essential evidence-based components of first-episode psychosis services. Psychiatr Serv. 2013 May 1;64(5):452-7. doi: 10.1176/appi.ps.201200156. PMID: 23370444. *Study design*

6. Afonso AM, Alfonso S, Morgan TO. Short-term Impact of Meaningful Use Stage 1 Implementation: A Comparison of Health Outcomes in 2 Primary Care Clinics. J Ambul Care Manage. 2017 Oct/Dec;40(4):316-26. doi: 10.1097/jac.0000000000000179. PMID: 28350638. *Intervention*

7. Anton RF, O'Malley SS, Ciraulo DA, et al. Combined pharmacotherapies and behavioral interventions for alcohol dependence: the COMBINE study: a randomized controlled trial. JAMA. 2006 May 3;295(17):2003-17. doi: 10.1001/jama.295.17.2003. PMID: 16670409. *Intervention*

8. Anton RF, Oroszi G, O'Malley S, et al. An evaluation of mu-opioid receptor (OPRM1) as a predictor of naltrexone response in the treatment of alcohol dependence: results from the Combined Pharmacotherapies and Behavioral Interventions for Alcohol Dependence (COMBINE) study. Arch Gen Psychiatry. 2008 Feb;65(2):135-44. doi: 10.1001/archpsyc.65.2.135. PMID: 18250251. *Intervention*

9. Apter AJ, Wang X, Bogen DK, et al. Problem solving to improve adherence and asthma outcomes in urban adults with moderate or severe asthma: a randomized controlled trial. J Allergy Clin Immunol. 2011 Sep;128(3):516-23 e1-5. doi: 10.1016/j.jaci.2011.05.010. PMID: 21704360. *Intervention*

10. Arends I, Bultmann U, Nielsen K, et al. Process evaluation of a problem solving intervention to prevent recurrent sickness absence in workers with common mental disorders. Soc Sci Med. 2014 Jan;100:123-32. doi: 10.1016/j.socscimed.2013.10.041. PMID: 24444847. *Study design*

11. Atfeh MS, Richardson-May J, Rainsbury J. Day case paediatric tonsillectomy: a quality improvement project. Eur J Pediatr. 2018 Nov;177(11):1603-8. doi: 10.1007/s00431-018-3220-0. PMID: 30058010. *Setting*

12. Bailie R, Matthews V, Larkins S, et al. Impact of policy support on uptake of evidence-based continuous quality improvement activities and the quality of care for Indigenous Australians: a comparative case study. BMJ Open. 2017 Oct 5;7(10):e016626. doi: 10.1136/bmjopen-2017-016626. PMID: 28982818. *Intervention*

13. Barnett AW. Structured Physical Activity and Dietary Education Program for Obese Adolescents: An Evaluation of a Quality Improvement Project at a Rural Primary Care Clinic. Structured Physical Activity & Dietary Education Program for Obese Adolescents: An Evaluation of a Quality Improvement Project at a Rural Primary Care Clinic. 2017:1-. PMID: 129593024. Language: English. Entry Date: 20180516. Revision Date: 20180516. Publication Type: Article. *Intervention*

14. Becker WC, Edmond SN, Cervone DJ, et al. Evaluation of an integrated, multidisciplinary program to address unsafe use of opioids prescribed for pain. Pain Medicine (United States). 2018;19(7):1419-24. doi: 10.1093/pm/pnx041. *Intervention*

15. Bergman AA, Hamilton AB, Chrystal JG, et al. Primary Care Providers' Perspectives on Providing Care to Women Veterans with Histories of Sexual Trauma. Womens Health Issues. 2019 Jul - Aug;29(4):325-32. doi: 10.1016/j.whi.2019.03.001. PMID: 31027706. *Intervention*

16. Bernhardsson S, Larsson MEH. Does a tailored guideline implementation strategy have an impact on clinical physiotherapy practice? A nonrandomized controlled study. J Eval Clin Pract. 2019 Aug;25(4):575-84. doi: 10.1111/jep.12958. PMID: 29806112. *Setting*

17. Bhagwat MM, Woods JA, Dronavalli M, et al. Evidence-based interventions in primary care following acute coronary syndrome in Australia and New Zealand: a systematic scoping review. BMC Cardiovasc Disord. 2016 Nov 9;16(1):214. doi: 10.1186/s12872-016-0388-y. PMID: 27829379. *Study design*

18. Binkley CJ, Johnson KW, Abadi M, et al. Improving the oral health of residents with intellectual and developmental disabilities: an oral health strategy and pilot study. Eval Program Plann. 2014 Dec;47:54-63. doi: 10.1016/j.evalprogplan.2014.07.003. PMID: 25137553. *Intervention*

19. Bird ML, Mortenson WB, Eng JJ. Evaluation and facilitation of intervention fidelity in community exercise programs through an adaptation of the TIDier framework. BMC Health Serv Res. 2020 Jan 30;20(1):68. doi: 10.1186/s12913-020-4919-y. PMID: 32000776. *Intervention*

20. Bohl DD, Ondeck NT, Basques BA, et al. What Is the Timing of General Health Adverse Events That Occur After Total Joint Arthroplasty? Clin Orthop Relat Res. 2017 Dec;475(12):2952-9. doi: 10.1007/s11999-016-5224-2. PMID: 28054326. *Intervention*

21. Bond G, Williams J, Evans L, et al. Psychiatric Rehabilitation Fidelity Toolkit Human Services Research Institute

U.S. Department of Health and Human Services. Cambridge, MA: 2000. *Duplicate*

22. Bond GR, Picone J, Mauer E. The Quality of Supported Employment Implementation Scale. 1998. *Intervention*

23. Booth HP, Gallagher AM, Mullett D, et al. Quality improvement of prescribing safety: a pilot study in primary care using UK electronic health records. Br J Gen Pract. 2019 Sep;69(686):e605-e11. doi: 10.3399/bjgp19X704597. PMID: 31262845. *Setting*

24. Booth R. HIV and HCV Risk Reduction Interventions in Drug Detoxification and Treatment Settings (Protocol for NIDA-CTN-0017) Addiction Research and Treatment Services, Department of Psychiatry, University of Colorado Health Sciences Center. Denver, CO: 2005. http://ctndisseminationlibrary.org/protocols/ctn0017.htm. *Intervention*

25. Booth RE, Campbell BK, Mikulich-Gilbertson SK, et al. Reducing HIV-related risk behaviors among injection drug users in residential detoxification. AIDS Behav. 2011 Jan;15(1):30-44. doi: 10.1007/s10461-010-9751-7. PMID: 20652630. *Intervention*

26. Brody AM, Miller J, Polevoy R, et al. Institutional Pathways to Improve Care of Patients with Elevated Blood Pressure in the Emergency Department. Curr Hypertens Rep. 2018 Apr 10;20(4):30. doi: 10.1007/s11906-018-0831-9. PMID: 29637311. *Study design*

27. Brower LH, Wilson PM, Murtagh Kurowski E, et al. Using Quality Improvement to Implement a Standardized Approach to Neonatal Herpes Simplex Virus. Pediatrics. 2019 Aug;144(2). doi: 10.1542/peds.2018-0262. PMID: 31345997. *Setting*

28. Brug J, Spikmans F, Aartsen C, et al. Training dietitians in basic motivational interviewing skills results in changes in their counseling style and in lower saturated fat intakes in their patients. J Nutr Educ Behav. 2007 Jan-Feb;39(1):8-12. doi: 10.1016/j.jneb.2006.08.010. PMID: 17276321. *Intervention*

29. Brunner J, Cain CL, Yano EM, et al. Local Leaders' Perspectives on Women Veterans' Health Care: What Would Ideal Look Like? Womens Health Issues. 2019 Jan - Feb;29(1):64-71. doi: 10.1016/j.whi.2018.10.005. PMID: 30455089. *Intervention*

30. Brunner J, Chuang E, Washington DL, et al. Patient-Rated Access to Needed Care: Patient-Centered Medical Home Principles Intertwined. Womens Health Issues. 2018 Mar - Apr;28(2):165-71. doi: 10.1016/j.whi.2017.12.001. PMID: 29339012. *Intervention*

31. Bruyndonckx R, Verhoeven V, Anthierens S, et al. The implementation of academic detailing and its effectiveness on appropriate prescribing of pain relief medication: a real-world cluster randomized trial in Belgian general practices. Implement Sci. 2018 Jan 10;13(1):6. doi: 10.1186/s13012-017-0703-8. PMID: 29316945. *Setting*

32. Bryant C, VanGraafeiland B. Screening for Adverse Childhood Experiences in Primary Care: A Quality Improvement Project. J Pediatr Health Care. 2020 Mar - Apr;34(2):122-7. doi: 10.1016/j.pedhc.2019.09.001. PMID: 31704177. *Intervention*

33. Burt MR, Duke AE, Hargreaves WA. The Program Environment Scale: assessing client perceptions of community-based programs for the severely mentally ill. Am J Community Psychol. 1998 Dec;26(6):853-79. doi: 10.1023/a:1022246112973. PMID: 10085535. *Intervention*

34. Calo WA, Gilkey MB, Leeman J, et al. Coaching primary care clinics for HPV vaccination quality improvement: Comparing in-person and webinar implementation. Transl Behav Med. 2019 Jan 1;9(1):23-31. doi: 10.1093/tbm/iby008. PMID: 29471460. *Intervention*

35. Calsyn DA. HIV/STD Safer Sex Skills Groups for Men in Methadone Maintenance or Drug-free Outpatient Treatment Programs (Protocol for NIDA-CTN-0019) Alcohol and Drug Abuse Institute, University of Washington. Seattle, WA: 2004. *Intervention*

36. Carroll KM, Ball SA, Nich C, et al. Motivational interviewing to improve treatment engagement and outcome in individuals seeking treatment for substance abuse: a multisite effectiveness study. Drug Alcohol Depend. 2006 Feb 28;81(3):301-12. doi: 10.1016/j.drugalcdep.2005.08.002. PMID: 16169159. *Intervention*

37. Carroll KM, Farentinos C, Ball SA, et al. MET meets the real world: design issues and clinical strategies in the Clinical Trials Network. J Subst Abuse Treat. 2002 Sep;23(2):73-80. doi: 10.1016/s0740-5472(02)00255-6. PMID: 12220604. *Intervention*

38. Chalasani S, Peiris DP, Usherwood T, et al. Reducing cardiovascular disease risk in diabetes: a randomised controlled trial of a quality improvement initiative. Med J Aust. 2017 Jun 5;206(10):436-41. doi: 10.5694/mja16.00332. PMID: 28566070. *Setting*

39. Cheung KS, Lau BH, Wong PW, et al. Multicomponent intervention on enhancing dementia caregiver well-being and reducing behavioral problems among Hong Kong Chinese: a translational study based on REACH II. Int J Geriatr Psychiatry. 2015 May;30(5):460-9. doi: 10.1002/gps.4160. PMID: 25043378. *Intervention*

40. Chuang E, Brunner J, Mak S, et al. Challenges with Implementing a Patient-Centered Medical Home Model for Women Veterans. Womens Health Issues. 2017 Mar - Apr;27(2):214-20. doi: 10.1016/j.whi.2016.11.005. PMID: 28063848. *Intervention*

41. Crits-Christoph P, Siqueland L, Blaine J, et al. The National Institute on Drug Abuse Collaborative Cocaine Treatment Study. Rationale and methods. Arch Gen Psychiatry. 1997 Aug;54(8):721-6. doi: 10.1001/archpsyc.1997.01830200053007. PMID: 9283507. *Intervention*

42. Crits-Christoph P, Siqueland L, Chittams J, et al. Training in cognitive, supportive-expressive, and drug counseling therapies for cocaine dependence. J Consult Clin Psychol. 1998 Jun;66(3):484-92. doi: 10.1037//0022-006x.66.3.484. PMID: 9642886. *Intervention*

43. Cucciare MA, Curran GM, Craske MG, et al. Assessing fidelity of cognitive behavioral therapy in rural VA clinics: design of a randomized implementation effectiveness (hybrid type III) trial. Implement Sci. 2016 May 10;11:65. doi: 10.1186/s13012-016-0432-4. PMID: 27164866. *Study design*

44. Cykert S, Lefebvre A, Bacon T, et al. Meaningful Use in Chronic Care: Improved Diabetes Outcomes Using a Primary Care Extension Center Model. N C Med J. 2016 Nov-Dec;77(6):378-83. doi: 10.18043/ncm.77.6.378. PMID: 27864481. *Study design*

45. Dale V, Heather N, Adamson S, et al. Predicting drinking outcomes: Evidence from the United Kingdom Alcohol Treatment Trial (UKATT). Addict Behav. 2017 Aug;71:61-7. doi: 10.1016/j.addbeh.2017.02.023. PMID: 28273487. *Intervention*

46. Dennis M, Godley SH, Diamond G, et al. The Cannabis Youth Treatment (CYT) Study: main findings from two randomized trials. J Subst Abuse Treat. 2004 Oct;27(3):197-213. doi: 10.1016/j.jsat.2003.09.005. PMID: 15501373. *Intervention*

47. Dennis M, Titus JC, Diamond G, et al. The Cannabis Youth Treatment (CYT) experiment: rationale, study design and analysis plans. Addiction. 2002 Dec;97 Suppl 1:16-34. doi: 10.1046/j.1360-0443.97.s01.2.x. PMID: 12460126. *Intervention*

48. Deri Armstrong C, Taljaard M, Hogg W, et al. Practice facilitation for improving cardiovascular care: secondary evaluation of a stepped wedge cluster randomized controlled trial using population-based administrative data. Trials. 2016 Sep 5;17(1):434. doi: 10.1186/s13063-016-1547-2. PMID: 27596224. *Study design*

49. Di Mario S, Gagliotti C, Buttazzi R, et al. Observational pre-post study showed that a quality improvement project reduced paediatric antibiotic prescribing rates in primary care. Acta Paediatr. 2018 Oct;107(10):1805-9. doi: 10.1111/apa.14381. PMID: 29723913. *Setting*

50. Dickinson WP, Dickinson LM, Jortberg BT, et al. A Cluster Randomized Trial Comparing Strategies for Translating Self-Management Support into Primary Care Practices. J Am Board Fam Med. 2019 May-Jun;32(3):341-52. doi: 10.3122/jabfm.2019.03.180254. PMID: 31068398. *Intervention*

51. Djalali S, Senn O. [Trends in family medicine--how to sort the wheat from the chaff]. Praxis (Bern 1994). 2015 Nov 11;104(23):1251-8. doi: 10.1024/1661-8157/a002176. PMID: 26558930. *Study design*

52. Doane J, Buu J, Penrod MJ, et al. Measuring and Managing Blood Pressure in a Primary Care Setting: A Pragmatic Implementation Study. J Am Board Fam Med. 2018 May-Jun;31(3):375-88. doi: 10.3122/jabfm.2018.03.170450. PMID: 29743221. *Intervention*

53. Donovan DM, Anton RF, Miller WR, et al. Combined pharmacotherapies and behavioral interventions for alcohol dependence (The COMBINE Study): examination of posttreatment drinking outcomes. J Stud Alcohol Drugs. 2008 Jan;69(1):5-13. doi: 10.15288/jsad.2008.69.5. PMID: 18080059. *Intervention*

54. Drake RE, Goldman HH, Leff HS, et al. Implementing evidence-based practices in routine mental health service settings. Psychiatr Serv. 2001 Feb;52(2):179-82. doi: 10.1176/appi.ps.52.2.179. PMID: 11157115. *Study design*

55. Duff J, Walker K, Omari A, et al. Educational outreach visits to improve nurses' use of mechanical venous thromboembolism prevention in hospitalized medical patients. J Vasc Nurs. 2013 Dec;31(4):139-49. doi: 10.1016/j.jvn.2013.04.002. PMID: 24238096. *Intervention*

56. Epstein JN, Langberg JM, Lichtenstein PK, et al. The myADHDportal.com improvement program: An innovative quality improvement intervention for improving the quality of ADHD care among community-based pediatricians. Clinical Practice in Pediatric Psychology. 2013;1(1):55-67. doi: 10.1037/cpp0000004. *Duplicate*

57. Etxeberria A, Alcorta I, Perez I, et al. Results from the CLUES study: a cluster randomized trial for the evaluation of cardiovascular guideline implementation in primary care in Spain. BMC Health Serv Res. 2018 Feb 8;18(1):93. doi: 10.1186/s12913-018-2863-x. PMID: 29422049. *Setting*

58. Evans LJ, Resnick SG, Bond GR. The Psychiatric Rehabilitation Environment Scale. 1998. *Study design*

59. Farkas MD, Cohen MR, Nemec PB. Psychiatric rehabilitation programs: putting concepts into practice? Community Ment Health J. 1988 Spring;24(1):7-21. doi: 10.1007/bf00755049. PMID: 3370939. *Intervention*

60. Farmer A, Wade A, Goyder E, et al. Impact of self monitoring of blood glucose in the management of patients with non-insulin treated diabetes: open parallel group randomised trial. BMJ. 2007 Jul 21;335(7611):132. doi: 10.1136/bmj.39247.447431.BE. PMID: 17591623. *Intervention*

61. Feaster DJ, Robbins MS, Horigian V, et al. Statistical issues in multisite effectiveness trials: the case of brief strategic family therapy for adolescent drug abuse treatment. Clin Trials. 2004;1(5):428-39. doi: 10.1191/1740774504cn041oa. PMID: 16279281. *Intervention*

62. Forgatch MS, Patterson GR, DeGarmo DS. Evaluating fidelity: predictive validity for a measure of competent adherence to the Oregon model of parent management training. Behav Ther. 2005;36(1):3-13. doi: 10.1016/s0005-7894(05)80049-8. PMID: 16718302. *Intervention*

63. Foster-Staples PA. Implementation of the National Asthma Guidelines in a Residential Pediatric Clinic. Implementation of the National Asthma Guidelines in a Residential Pediatric Clinic. 2017:1-. PMID: 129432718. Language: English. Entry Date: 20180511. Revision Date: 20180511. Publication Type: Article. *Intervention*

64. Fox AB, Hamilton AB, Frayne SM, et al. Effectiveness of an evidence-based quality improvement approach to cultural competence training: The veterans affairs’ “Caring for Women Veterans” program. Journal of Continuing Education in the Health Professions. 2016;36(2):96-103. doi: 10.1097/CEH.0000000000000073. *Duplicate*

65. Gabbay RA, Anel-Tiangco RM, Dellasega C, et al. Diabetes nurse case management and motivational interviewing for change (DYNAMIC): results of a 2-year randomized controlled pragmatic trial. J Diabetes. 2013 Sep;5(3):349-57. doi: 10.1111/1753-0407.12030. PMID: 23368423. *Study design*

66. Gabel F, Chambers R, Cox T, et al. An evaluation of a multifaceted, local Quality Improvement Framework for long-term conditions in UK primary care. Fam Pract. 2018 Dec 21. doi: 10.1093/fampra/cmy128. PMID: 30576438. *Setting*

67. Gellert GA, Davenport CM, Minard CG, et al. Reducing pediatric asthma hospital length of stay through evidence-based quality improvement and deployment of computerized provider order entry. Journal of Asthma. 2020;57(2):123-35. doi: 10.1080/02770903.2018.1553053. *Duplicate*

68. Gleeson H, Calderon A, Swami V, et al. Systematic review of approaches to using patient experience data for quality improvement in healthcare settings. BMJ Open. 2016 Aug 16;6(8):e011907. doi: 10.1136/bmjopen-2016-011907. PMID: 27531733. *Study design*

69. Goyder E, Hind D, Breckon J, et al. A randomised controlled trial and cost-effectiveness evaluation of 'booster' interventions to sustain increases in physical activity in middle-aged adults in deprived urban neighbourhoods. Health Technol Assess. 2014 Feb;18(13):1-210. doi: 10.3310/hta18130. PMID: 24571932. *Intervention*

70. Group CSR. Testing combined pharmacotherapies and behavioral interventions in alcohol dependence: rationale and methods. Alcohol Clin Exp Res. 2003 Jul;27(7):1107-22. doi: 10.1097/00000374-200307000-00011. PMID: 12878917. *Intervention*

71. Group CSR. Testing combined pharmacotherapies and behavioral interventions for alcohol dependence (the COMBINE study): a pilot feasibility study. Alcohol Clin Exp Res. 2003 Jul;27(7):1123-31. doi: 10.1097/01.ALC.0000078020.92938.0B. PMID: 12878918. *Intervention*

72. Grubbs KM, Cheney AM, Fortney JC, et al. The role of gender in moderating treatment outcome in collaborative care for anxiety. Psychiatr Serv. 2015 Mar 1;66(3):265-71. doi: 10.1176/appi.ps.201400049. PMID: 25727114. *Intervention*

73. Hains I, Meyers J, Sterling K, et al. Difficult-to-treat and severe asthma in general practice: delivery and evaluation of an educational program. BMC Fam Pract. 2019 Jul 13;20(1):99. doi: 10.1186/s12875-019-0991-y. PMID: 31301742. *Intervention*

74. Hanna-Bull D. Preventing heel pressure ulcers. Journal of Wound, Ostomy and Continence Nursing. 2016;43(2):129-32. doi: 10.1097/WON.0000000000000181. *Duplicate*

75. Hardeman W, Michie S, Fanshawe T, et al. Fidelity of delivery of a physical activity intervention: predictors and consequences. Psychol Health. 2008;23(1):11-24. doi: 10.1080/08870440701615948. PMID: 25159904. *Intervention*

76. Harris MF, Parker SM, Litt J, et al. An Australian general practice based strategy to improve chronic disease prevention, and its impact on patient reported outcomes: evaluation of the preventive evidence into practice cluster randomised controlled trial. BMC Health Serv Res. 2017 Sep 8;17(1):637. doi: 10.1186/s12913-017-2586-4. PMID: 28886739. *Setting*

77. Harting J, van Assema P, van der Molen HT, et al. Quality assessment of health counseling: performance of health advisors in cardiovascular prevention. Patient Educ Couns. 2004 Jul;54(1):107-18. doi: 10.1016/S0738-3991(03)00194-0. PMID: 15210268. *Study design*

78. Hartveit M, Hovlid E, Nordin MHA, et al. Measuring implementation: development of the implementation process assessment tool (IPAT). BMC Health Serv Res. 2019 Oct 21;19(1):721. doi: 10.1186/s12913-019-4496-0. PMID: 31638967. *Intervention*

79. Hein D. Women’s Treatment for Trauma and Substance Use Disorders: A Randomized Clinical Trial (NIDA-CTN-0015). Social Intervention Group, Columbia University School of Social Work. New York, NY: 2005. https://adai.uw.edu/research_project/nida-ctn-0015-womens-treatment-for-trauma-and-substance-use-disorders/. *Intervention*

80. Henggeler SW, Schoenwald SK, Liao JG, et al. Transporting efficacious treatments to field settings: the link between supervisory practices and therapist fidelity in MST programs. J Clin Child Adolesc Psychol. 2002 Jun;31(2):155-67. doi: 10.1207/S15374424JCCP3102_02. PMID: 12056100. *Intervention*

81. Herbert C, Winkler H, Moore TA. Outcomes of mental health pharmacist-managed electronic consults at a Veterans Affairs health care system. Ment Health Clin. 2017 May;7(3):131-6. doi: 10.9740/mhc.2017.05.131. PMID: 29955511. *Study design*

82. Hermens RP, Hak E, Hulscher ME, et al. Adherence to guidelines on cervical cancer screening in general practice: programme elements of successful implementation. Br J Gen Pract. 2001 Nov;51(472):897-903. PMID: 11761203. *Intervention*

83. Hien DA, Wells EA, Jiang H, et al. Multisite randomized trial of behavioral interventions for women with co-occurring PTSD and substance use disorders. J Consult Clin Psychol. 2009 Aug;77(4):607-19. doi: 10.1037/a0016227. PMID: 19634955. *Intervention*

84. Hunt K, Stiller K. Dietetic and educational interventions improve clinical outcomes of diabetic and obese clients with mental impairment. Nutr Diet. 2017 Jul;74(3):236-42. doi: 10.1111/1747-0080.12340. PMID: 28731608. *Setting*

85. Hunt MK, Lobb R, Delichatsios HK, et al. Process evaluation of a clinical preventive nutrition intervention. Prev Med. 2001 Aug;33(2 Pt 1):82-90. doi: 10.1006/pmed.2001.0863. PMID: 11493040. *Intervention*

86. Ike B, Baldwin LM, Sutton S, et al. Staff and Clinician Work-Life Perceptions after Implementing Systems-Based Improvements to Opioid Management. J Am Board Fam Med. 2019 Sep-Oct;32(5):715-23. doi: 10.3122/jabfm.2019.05.190027. PMID: 31506367. *Intervention*

87. Jager C, Freund T, Steinhauser J, et al. Impact of a tailored program on the implementation of evidence-based recommendations for multimorbid patients with polypharmacy in primary care practices-results of a cluster-randomized controlled trial. Implement Sci. 2017 Jan 13;12(1):8. doi: 10.1186/s13012-016-0535-y. PMID: 28086976. *Setting*

88. Jansink R, Braspenning J, Keizer E, et al. No identifiable Hb1Ac or lifestyle change after a comprehensive diabetes programme including motivational interviewing: a cluster randomised trial. Scand J Prim Health Care. 2013 Jun;31(2):119-27. doi: 10.3109/02813432.2013.797178. PMID: 23659710. *Intervention*

89. Kanamori S, Shibanuma A, Jimba M. Applicability of the 5S management method for quality improvement in health-care facilities: a review. Trop Med Health. 2016;44:21. doi: 10.1186/s41182-016-0022-9. PMID: 27462185. *Study design*

90. Keith RE, Crosson JC, O'Malley AS, et al. Using the Consolidated Framework for Implementation Research (CFIR) to produce actionable findings: a rapid-cycle evaluation approach to improving implementation. Implement Sci. 2017 Feb 10;12(1):15. doi: 10.1186/s13012-017-0550-7. PMID: 28187747. *Outcome*

91. Kennedy S, Bailey R, Jaffee K, et al. Effectiveness of Evidence-Based Asthma Interventions. Pediatrics. 2017 Jun;139(6). doi: 10.1542/peds.2016-4221. PMID: 28562279. *Intervention*

92. Kidd TL. Improving Asthma in Pediatric Practice. Journal of Pediatric Nursing. 2019;49:109-. doi: 10.1016/j.pedn.2019.09.013. PMID: 140957531. Language: English. Entry Date: 20200103. Revision Date: 20200106. Publication Type: Article. Journal Subset: Core Nursing. *Study design*

93. Kingma AEC, van Stel HF, Oudega R, et al. Multi-faceted implementation strategy to increase use of a clinical guideline for the diagnosis of deep venous thrombosis in primary care. Fam Pract. 2017 Aug 1;34(4):446-51. doi: 10.1093/fampra/cmw066. PMID: 27471223. *Setting*

94. Kraft S, Caplan W, Trowbridge E, et al. Building the learning health system: Describing an organizational infrastructure to support continuous learning. Learn Health Syst. 2017 Oct;1(4):e10034. doi: 10.1002/lrh2.10034. PMID: 31245569. *Setting*

95. Kramer BJ, Creekmur B, Howe JL, et al. Veterans Affairs Geriatric Scholars Program: Enhancing Existing Primary Care Clinician Skills in Caring for Older Veterans. J Am Geriatr Soc. 2016 Nov;64(11):2343-8. doi: 10.1111/jgs.14382. PMID: 27696341. *Study design*

96. Lane C, Huws-Thomas M, Hood K, et al. Measuring adaptations of motivational interviewing: the development and validation of the behavior change counseling index (BECCI). Patient Educ Couns. 2005 Feb;56(2):166-73. doi: 10.1016/j.pec.2004.01.003. PMID: 15653245. *Study design*

97. Lawrence W, Black C, Tinati T, et al. 'Making every contact count': Evaluation of the impact of an intervention to train health and social care practitioners in skills to support health behaviour change. J Health Psychol. 2016 Feb;21(2):138-51. doi: 10.1177/1359105314523304. PMID: 24713156. *Study design*

98. Lawton R, Heyhoe J, Louch G, et al. Using the Theoretical Domains Framework (TDF) to understand adherence to multiple evidence-based indicators in primary care: a qualitative study. Implement Sci. 2016 Aug 8;11:113. doi: 10.1186/s13012-016-0479-2. PMID: 27502590. *Study design*

99. Lazovich D, Curry SJ, Beresford SA, et al. Implementing a dietary intervention in primary care practice: a process evaluation. Am J Health Promot. 2000 Nov-Dec;15(2):118-25. doi: 10.4278/0890-1171-15.2.118. PMID: 11194695. *Intervention*

100. Leggio L, Ray LA, Kenna GA, et al. Blood glucose level, alcohol heavy drinking, and alcohol craving during treatment for alcohol dependence: results from the Combined Pharmacotherapies and Behavioral Interventions for Alcohol Dependence (COMBINE) Study. Alcohol Clin Exp Res. 2009 Sep;33(9):1539-44. doi: 10.1111/j.1530-0277.2009.00982.x. PMID: 19485973. *Intervention*

101. Lewis CL, Dalton AF, Drake L, et al. Developing and Evaluating a Clinic-Based Decision Aid Delivery System. MDM Policy Pract. 2016 Jul-Dec;1(1):2381468316656850. doi: 10.1177/2381468316656850. PMID: 30288402. *Study design*

102. Liddy C, Bello A, Cook J, et al. Supporting the spread and scale-up of electronic consultation across Canada: cross-sectional analysis. BMJ Open. 2019 May 30;9(5):e028888. doi: 10.1136/bmjopen-2018-028888. PMID: 31152043. *Study design*

103. Lipshutz AKM, Fee C, Schell H, et al. Strategies for success: A PDSA analysis of three QI initiatives in critical care. Joint Commission Journal on Quality and Patient Safety. 2008;34(8):435-44. doi: 10.1016/S1553-7250(08)34054-9. *Duplicate*

104. Liss DT, Peprah YA, Brown T, et al. Using Electronic Health Records to Measure Quality Improvement Efforts: Findings from a Large Practice Facilitation Initiative. Joint Commission Journal on Quality & Patient Safety. 2020;46(1):11-7. doi: 10.1016/j.jcjq.2019.09.006. PMID: 140293779. Language: English. Entry Date: 20191214. Revision Date: 20191214. Publication Type: Article. Journal Subset: Editorial Board Reviewed. *Study design*

105. Loeb DF, Kline DM, Kroenke K, et al. Designing the relational team development intervention to improve management of mental health in primary care using iterative stakeholder engagement. BMC Fam Pract. 2019 Sep 6;20(1):124. doi: 10.1186/s12875-019-1010-z. PMID: 31492096. *Outcome*

106. Loeb DF, Monson SP, Lockhart S, et al. Mixed method evaluation of Relational Team Development (RELATED) to improve team-based care for complex patients with mental illness in primary care. BMC Psychiatry. 2019 Oct 15;19(1):299. doi: 10.1186/s12888-019-2294-1. PMID: 31615460. *Outcome*

107. Lorencatto F, West R, Bruguera C, et al. A method for assessing fidelity of delivery of telephone behavioral support for smoking cessation. J Consult Clin Psychol. 2014 Jun;82(3):482-91. doi: 10.1037/a0035149. PMID: 24294836. *Intervention*

108. Maas MJ, Nijhuis-van der Sanden MW, Driehuis F, et al. Feasibility of peer assessment and clinical audit to self-regulate the quality of physiotherapy services: a mixed methods study. BMJ Open. 2017 Feb 10;7(2):e013726. doi: 10.1136/bmjopen-2016-013726. PMID: 28188156. *Setting*

109. Machline-Carrion MJ, Soares RM, Damiani LP, et al. Effect of a Multifaceted Quality Improvement Intervention on the Prescription of Evidence-Based Treatment in Patients at High Cardiovascular Risk in Brazil: The BRIDGE Cardiovascular Prevention Cluster Randomized Clinical Trial. JAMA Cardiol. 2019 May 1;4(5):408-17. doi: 10.1001/jamacardio.2019.0649. PMID: 30942842. *Setting*

110. Mader EM, Fox CH, Epling JW, et al. A Practice Facilitation and Academic Detailing Intervention Can Improve Cancer Screening Rates in Primary Care Safety Net Clinics. J Am Board Fam Med. 2016 Sep-Oct;29(5):533-42. doi: 10.3122/jabfm.2016.05.160109. PMID: 27613786. *Study design*

111. Marang-Van De Mheen PJ, Van Bodegom-Vos L. Meta-analysis of the central line bundle for preventing catheterrelated infections: A case study in appraising the evidence in quality improvement. BMJ Quality and Safety. 2016;25(2):118-29. doi: 10.1136/bmjqs-2014-003787. *Duplicate*

112. Marshall M, Pfeifer N, de Silva D, et al. An evaluation of a safety improvement intervention in care homes in England: a participatory qualitative study. J R Soc Med. 2018 Nov;111(11):414-21. doi: 10.1177/0141076818803457. PMID: 30235053. *Setting*

113. Maru DSR, Andrews J, Schwarz D, et al. Crossing the quality chasm in resource-limited settings. Globalization and Health. 2012;8. doi: 10.1186/1744-8603-8-41. *Duplicate*

114. McCarthy MM, Dickson VV, Katz SD, et al. Process evaluation of an exercise counseling intervention using motivational interviewing. Appl Nurs Res. 2015 May;28(2):156-62. doi: 10.1016/j.apnr.2014.09.006. PMID: 25448059. *Intervention*

115. McGillion MH, Watt-Watson J, Stevens B, et al. Randomized controlled trial of a psychoeducation program for the self-management of chronic cardiac pain. J Pain Symptom Manage. 2008 Aug;36(2):126-40. doi: 10.1016/j.jpainsymman.2007.09.015. PMID: 18395397. *Intervention*

116. McGrew JH, Pescosolido B, Wright E. Case managers’ perspectives on critical ingredients of assertive community treatment and on its implementation. Psychiatric Services. 2003;54(370-376). *Intervention*

117. Meredith LS, Azhar G, Okunogbe A, et al. Primary Care Providers with More Experience and Stronger Self-Efficacy Beliefs Regarding Women Veterans Screen More Frequently for Interpersonal Violence. Womens Health Issues. 2017 Sep - Oct;27(5):586-91. doi: 10.1016/j.whi.2017.06.003. PMID: 28754476. *Intervention*

118. Meredith LS, Wang Y, Okunogbe A, et al. Attitudes, Practices, and Experiences with Implementing a Patient-Centered Medical Home for Women Veterans. Womens Health Issues. 2017 Mar - Apr;27(2):221-7. doi: 10.1016/j.whi.2016.11.008. PMID: 28087130. *Intervention*

119. Miller WR, Moyers TB, Arciniega L, et al. Training, supervision and quality monitoring of the COMBINE Study behavioral interventions. J Stud Alcohol Suppl. 2005 Jul(15):188-95; discussion 68-9. doi: 10.15288/jsas.2005.s15.188. PMID: 16223070. *Intervention*

120. Mills SC, Ragan TJ. A tool for analyzing implementation fidelity of an integrated learning system (ILS). Educational Technology Research and Development. 2000;48:21-41. *Setting*

121. Montejo Fernandez M, Benito Manrique I, Montiel Eguia A, et al. [An initiative to reduce the use of unnecessary medication in infants with bronchiolitis in primary care]. An Pediatr (Barc). 2019 Jan;90(1):19-25. doi: 10.1016/j.anpedi.2018.02.016. PMID: 29803642. *Setting*

122. Morad Hameed S, Brenneman FD, Ball CG, et al. General surgery 2.0: The emergence of acute care surgery in Canada. Canadian Journal of Surgery. 2010;53(2):79-83. *Duplicate*

123. Moseng T, Dagfinrud H, Osteras N. Implementing international osteoarthritis guidelines in primary care: uptake and fidelity among health professionals and patients. Osteoarthritis Cartilage. 2019 Aug;27(8):1138-47. doi: 10.1016/j.joca.2019.03.010. PMID: 31075423. *Setting*

124. Moyers TB, Martin T, Manuel JK, et al. The Motivational Interviewing Treatment Integrity (MITI) Code: Version 2.0. In: Mexico UoN, editor Center on Alcoholism, Substance Abuse and Addictions (CASAA); 2003. *Study design*

125. Nagykaldi ZJ, Dave A, Kristof CJ, et al. Improving Patient-Clinician Conversations During Annual Wellness Visits. J Am Board Fam Med. 2017 Mar-Apr;30(2):161-9. doi: 10.3122/jabfm.2017.02.160229. PMID: 28379822. *Intervention*

126. Parker CN, Shuter P, Maresco-Pennisi D, et al. Implementation of the Champions for Skin Integrity model to improve leg and foot ulcer care in the primary healthcare setting. J Clin Nurs. 2019 Jul;28(13-14):2517-25. doi: 10.1111/jocn.14826. PMID: 30791154. *Setting*

127. Phillips A, Harmon JL, Bera J, et al. Integrating Preexposure Prophylaxis (PrEP) Into a Network of Community Health Centers. Journal for Nurse Practitioners. 2020. doi: 10.1016/j.nurpra.2019.09.010. *Intervention*

128. Pill R, Stott NC, Rollnick SR, et al. A randomized controlled trial of an intervention designed to improve the care given in general practice to Type II diabetic patients: patient outcomes and professional ability to change behaviour. Fam Pract. 1998 Jun;15(3):229-35. doi: 10.1093/fampra/15.3.229. PMID: 9694180. *Study design*

129. Piper AK. Facilitating the use of asthma action plans in primary care: A quality improvement project. Facilitating the Use of Asthma Action Plans in Primary Care: A Quality Improvement Project. 2017:1-. PMID: 123313480. Language: English. Entry Date: 20180427. Revision Date: 20180427. Publication Type: Abstract. *Outcome*

130. Post EP, Kilbourne AM, Bremer RW, et al. Organizational factors and depression management in community-based primary care settings. Implementation Science. 2009;4(1). doi: 10.1186/1748-5908-4-84. *Duplicate*

131. Potts S, Shields S, Upshur C. Preparing Future Leaders: An Integrated Quality Improvement Residency Curriculum. Fam Med. 2016 Jun;48(6):477-81. PMID: 27272426. *Study design*

132. Price JD, Jayaprakash M, McKay CM, et al. Evidence-Based Interventions for High Blood Pressure and Glycemic Control Among Illinois Health Systems. Prev Chronic Dis. 2020 Jan 23;17:E08. doi: 10.5888/pcd17.190058. PMID: 31971896. *Intervention*

133. Reichert SM, Harris SB, Tompkins JW, et al. Impact of a primary healthcare quality improvement program on diabetes in Canada: evaluation of the Quality Improvement and Innovation Partnership (QIIP). BMJ Open Diabetes Res Care. 2017;5(1):e000392. doi: 10.1136/bmjdrc-2017-000392. PMID: 29435348. *Setting*

134. Reid M. Smoking Cessation Treatment With Transdermal Nicotine Replacement Therapy in Substance Abuse Rehabilitation Programs (Protocol for NIDA-CTN-0009) Department of Psychiatry, New York University School of Medicine. New York, NY: 2004. http://ctndisseminationlibrary.org/protocols/ctn0009.htm. *Intervention*

135. Reid MS, Fallon B, Sonne S, et al. Smoking cessation treatment in community-based substance abuse rehabilitation programs. J Subst Abuse Treat. 2008 Jul;35(1):68-77. doi: 10.1016/j.jsat.2007.08.010. PMID: 17951021. *Intervention*

136. Robbins MS, Feaster DJ, Horigian VE, et al. Therapist adherence in brief strategic family therapy for adolescent drug abusers. J Consult Clin Psychol. 2011 Feb;79(1):43-53. doi: 10.1037/a0022146. PMID: 21261433. *Intervention*

137. Robbins MS, Horigian VE, Szapocznik J. [Brief strategic family therapy: an empirically-validated intervention for reducing adolescent behavior problems]. Prax Kinderpsychol Kinderpsychiatr. 2008;57(5):381-400. doi: 10.13109/prkk.2008.57.5.381. PMID: 18605440. *Intervention*

138. Robbins MS, Mayorga CC, Mitrani VB, et al. Adolescent and parent alliances with therapists in Brief Strategic Family Therapy with drug-using Hispanic adolescents. J Marital Fam Ther. 2008 Jul;34(3):316-28. doi: 10.1111/j.1752-0606.2008.00075.x. PMID: 18717922. *Intervention*

139. Robbins MS, Szapocznik J, Horigian VE, et al. Brief strategic family therapy for adolescent drug abusers: a multi-site effectiveness study. Contemp Clin Trials. 2009 May;30(3):269-78. doi: 10.1016/j.cct.2009.01.004. PMID: 19470315. *Intervention*

140. Rocker GM, Amar C, Laframboise WL, et al. Spreading improvements for advanced COPD care through a Canadian Collaborative. Int J Chron Obstruct Pulmon Dis. 2017;12:2157-64. doi: 10.2147/copd.S140043. PMID: 28794620. *Setting*

141. Roy-Byrne P, Craske MG, Sullivan G, et al. Delivery of evidence-based treatment for multiple anxiety disorders in primary care: a randomized controlled trial. JAMA. 2010 May 19;303(19):1921-8. doi: 10.1001/jama.2010.608. PMID: 20483968. *Intervention*

142. Russell G, Lane R, Parker S, et al. Preventive Evidence into Practice: what factors matter in a facilitation intervention to prevent vascular disease in family practice? BMC Fam Pract. 2019 Aug 8;20(1):113. doi: 10.1186/s12875-019-0995-7. PMID: 31395020. *Intervention*

143. Sanchez A, Farahi N, Flower KB, et al. Improved Breastfeeding Outcomes Following an On-site Support Intervention in an Academic Family Medicine Center. Fam Med. 2019 Nov;51(10):836-40. doi: 10.22454/FamMed.2019.698323. PMID: 31722101. *Intervention*

144. Sanchez A, Grandes G, Cortada JM, et al. Feasibility of an implementation strategy for the integration of health promotion in routine primary care: a quantitative process evaluation. BMC Fam Pract. 2017 Feb 17;18(1):24. doi: 10.1186/s12875-017-0585-5. PMID: 28212621. *Setting*

145. Sanchez A, Grandes G, Pablo S, et al. Engaging primary care professionals in collaborative processes for optimising type 2 diabetes prevention practice: the PREDIAPS cluster randomised type II hybrid implementation trial. Implement Sci. 2018 Jul 11;13(1):94. doi: 10.1186/s13012-018-0783-0. PMID: 29996928. *Study design*

146. Sarff L, O'Brien R. Evidence-Based Quality Improvement Training Programs: Building Staff Capability and Organizational Capacity. J Nurs Care Qual. 2020 Apr/Jun;35(2):95-101. doi: 10.1097/ncq.0000000000000416. PMID: 31136532. *Duplicate*

147. Schierhout G, Matthews V, Connors C, et al. Improvement in delivery of type 2 diabetes services differs by mode of care: a retrospective longitudinal analysis in the Aboriginal and Torres Strait Islander Primary Health Care setting. BMC Health Serv Res. 2016 Oct 7;16(1):560. doi: 10.1186/s12913-016-1812-9. PMID: 27717351. *Study design*

148. Schneider EC, Sorbero ME, Haas A, et al. Erratum to: Does a quality improvement campaign accelerate take-up of new evidence? A ten-state cluster-randomized controlled trial of the IHI's Project JOINTS. Implement Sci. 2017 May 10;12(1):59. doi: 10.1186/s13012-017-0591-y. PMID: 28490372. *Study design*

149. Schnipper JL, Mixon A, Stein J, et al. Effects of a multifaceted medication reconciliation quality improvement intervention on patient safety: final results of the MARQUIS study. BMJ Qual Saf. 2018 Dec;27(12):954-64. doi: 10.1136/bmjqs-2018-008233. PMID: 30126891. *Setting*

150. Schultz AA, Gallant P. Implementation forum. Evidence-based quality improvement project for determining appropriate discontinuation of peripheral intravenous cannulas. Evidence Based Nursing. 2005;8(1):8-. PMID: 106625720. Language: English. Entry Date: 20050506. Revision Date: 20150820. Publication Type: Journal Article. Journal Subset: Core Nursing. *Duplicate*

151. Sherwood G, Nickel B. Integrating Quality and Safety Competencies to Improve Outcomes: Application in Infusion Therapy Practice. J Infus Nurs. 2017 Mar/Apr;40(2):116-22. doi: 10.1097/nan.0000000000000210. PMID: 28248812. *Setting*

152. Sinha SK, Bennett J, Ramsden R, et al. Delivering improved patient and system outcomes for hospitalized older adults through an Acute Care for Elders Strategy. Healthc Manage Forum. 2018 Jul;31(4):126-32. doi: 10.1177/0840470418773108. PMID: 29952256. *Setting*

153. Skidmore ER, Dawson DR, Whyte EM, et al. Developing complex interventions: lessons learned from a pilot study examining strategy training in acute stroke rehabilitation. Clin Rehabil. 2014 Apr;28(4):378-87. doi: 10.1177/0269215513502799. PMID: 24113727. *Intervention*

154. Smith JL, Ritchie MJ, Kim B, et al. Getting to Fidelity: Identifying Core Components of Implementation Facilitation Strategies. VA HSR&D/QUERI National Conference, ‘Innovation to Impact: Research to Advance VA’s Learning Healthcare Community; 2019 October; Washington DC. *Study design*

155. Stanley MA, Calleo J, Bush AL, et al. The peaceful mind program: a pilot test of a cognitive-behavioral therapy-based intervention for anxious patients with dementia. Am J Geriatr Psychiatry. 2013 Jul;21(7):696-708. doi: 10.1016/j.jagp.2013.01.007. PMID: 23567399. *Intervention*

156. Stevans JM, Bise CG, McGee JC, et al. Knowledge Translation and Implementation Special Series. Evidence-Based Practice Implementation: Case Report of the Evolution of a Quality Improvement Program in a Multicenter Physical Therapy Organization. Physical Therapy. 2015;95(4):588-99. doi: 10.2522/ptj.20130541. PMID: 102061956. Language: English. Entry Date: 20150414. Revision Date: 20191111. Publication Type: Article. *Duplicate*

157. Svikis D. Job Seekers Training for Patients With Drug Dependence (Protocol for NIDA-CTN-0020). Department of Psychology, Virginia Commonwealth University. Richmond, VA: 2003. http://ctndisseminationlibrary.org/protocols/ctn0020.htm. *Intervention*

158. Svikis DS, Keyser-Marcus L, Stitzer M, et al. Randomized multi-site trial of the Job Seekers' Workshop in patients with substance use disorders. Drug Alcohol Depend. 2012 Jan 1;120(1-3):55-64. doi: 10.1016/j.drugalcdep.2011.06.024. PMID: 21802222. *Intervention*

159. Swinkels RA, Meerhoff GM, Custers JW, et al. Using Outcome Measures in Daily Practice: Development and Evaluation of an Implementation Strategy for Physiotherapists in the Netherlands. Physiother Can. 2015 Fall;67(4):357-64. doi: 10.3138/ptc.2014-28. PMID: 27504035. *Study design*

160. Szapocznik J. Brief Strategic Family Therapy for Adolescent Drug Abusers (Protocol for NIDA-CTN-0014). Center for Family Studies, University of Miami School of Medicine. Miami, FL: 2004. *Intervention*

161. Szapocznik J, Muir JA, Duff JH, et al. Brief Strategic Family Therapy: implementing evidence-based models in community settings. Psychother Res. 2015;25(1):121-33. doi: 10.1080/10503307.2013.856044. PMID: 24274187. *Intervention*

162. Szapocznik J, Schwartz SJ, Muir JA, et al. Brief Strategic Family Therapy: An Intervention to Reduce Adolescent Risk Behavior. Couple Family Psychol. 2012 Jun;1(2):134-45. doi: 10.1037/a0029002. PMID: 23936750. *Intervention*

163. Szapocznik J, Zarate M, Duff J, et al. Brief strategic family therapy: engaging drug using/problem behavior adolescents and their families in treatment. Soc Work Public Health. 2013;28(3-4):206-23. doi: 10.1080/19371918.2013.774666. PMID: 23731415. *Intervention*

164. Tate ML, Hopper S, Bergeron SP. Clinical and Economic Benefits of Pharmacist Involvement in a Community Hospital-Affiliated Patient-Centered Medical Home. J Manag Care Spec Pharm. 2018 Feb;24(2):160-4. doi: 10.18553/jmcp.2018.24.2.160. PMID: 29384022. *Intervention*

165. Teague GB, Bond GR, Drake RE. Program fidelity in assertive community treatment: development and use of a measure. Am J Orthopsychiatry. 1998 Apr;68(2):216-32. doi: 10.1037/h0080331. PMID: 9589760. *Duplicate*

166. Team UR. United Kingdom Alcohol Treatment Trial (UKATT): hypotheses, design and methods. Alcohol Alcohol. 2001 Jan-Feb;36(1):11-21. doi: 10.1093/alcalc/36.1.11. PMID: 11139410. *Intervention*

167. Teeter BS, Mosley C, Thomas JL, et al. Improving HPV vaccination using implementation strategies in community pharmacies: Pilot study protocol. Research in Social and Administrative Pharmacy. 2020;16(3):336-41. doi: 10.1016/j.sapharm.2019.05.022. *Duplicate*

168. Thyrian JR, Freyer-Adam J, Hannover W, et al. Adherence to the principles of Motivational Interviewing, clients' characteristics and behavior outcome in a smoking cessation and relapse prevention trial in women postpartum. Addict Behav. 2007 Oct;32(10):2297-303. doi: 10.1016/j.addbeh.2007.01.024. PMID: 17307300. *Intervention*

169. Thyrian JR, Freyer-Adam J, Hannover W, et al. Population-based smoking cessation in women post partum: adherence to motivational interviewing in relation to client characteristics and behavioural outcomes. Midwifery. 2010 Apr;26(2):202-10. doi: 10.1016/j.midw.2008.04.004. PMID: 18653261. *Intervention*

170. Ting JY, Goh VSK, Osiovich H. Reduction of central line-associated bloodstream infection rates in a neonatal intensive care unit after implementation of a multidisciplinary evidence-based quality improvement collaborative: A four-year surveillance. Canadian Journal of Infectious Diseases and Medical Microbiology. 2013;24(4):185-90. doi: 10.1155/2013/781690. *Duplicate*

171. Titsworth WL, Hester J, Correia T, et al. Reduction of catheter-associated urinary tract infections among patients in a neurological intensive care unit: A single institution's success: Clinical article. Journal of Neurosurgery. 2012;116(4):911-20. doi: 10.3171/2011.11.JNS11974. *Duplicate*

172. Tober G, Godfrey C, Parrott S, et al. Setting standards for training and competence: the UK alcohol treatment trial. Alcohol Alcohol. 2005 Sep-Oct;40(5):413-8. doi: 10.1093/alcalc/agh181. PMID: 16027128. *Intervention*

173. Tomasone JR, Martin Ginis KA, Estabrooks PA, et al. 'Changing minds': determining the effectiveness and key ingredients of an educational intervention to enhance healthcare professionals' intentions to prescribe physical activity to patients with physical disabilities. Implement Sci. 2014 Mar 1;9:30. doi: 10.1186/1748-5908-9-30. PMID: 24581329. *Study design*

174. Tross S. HIV/STD Safer Sex Skills Groups for Women in Methadone Maintenance or Drug-free Outpatient Treatment Programs (Protocol for NIDA-CTN-0019) HIV Center, New York State Psychiatric Institute. New York, NY: 2005. http://ctndisseminationlibrary.org/protocols/ctn0019.htm. *Intervention*

175. Tross S, Campbell AN, Cohen LR, et al. Effectiveness of HIV/STD sexual risk reduction groups for women in substance abuse treatment programs: results of NIDA Clinical Trials Network Trial. J Acquir Immune Defic Syndr. 2008 Aug 15;48(5):581-9. doi: 10.1097/QAI.0b013e31817efb6e. PMID: 18645513. *Intervention*

176. Unützer J, Schoenbaum M, Druss BG, et al. Transforming mental health care at the interface with general medicine: Report for the President's Commission. Psychiatric Services. 2006;57(1):37-47. doi: 10.1176/appi.ps.57.1.37. *Duplicate*

177. Urbiztondo I, Bjerrum L, Caballero L, et al. Decreasing Inappropriate Use of Antibiotics in Primary Care in Four Countries in South America-Cluster Randomized Controlled Trial. Antibiotics (Basel). 2017 Dec 14;6(4). doi: 10.3390/antibiotics6040038. PMID: 29240687. *Setting*

178. Wahlberg H, Braaten T, Broderstad AR. Impact of referral templates on patient experience of the referral and care process: a cluster randomised trial. BMJ Open. 2016 Oct 24;6(10):e011651. doi: 10.1136/bmjopen-2016-011651. PMID: 27797992. *Study design*

179. Wallace JI, Buchner DM, Grothaus L, et al. Implementation and effectiveness of a community-based health promotion program for older adults. J Gerontol A Biol Sci Med Sci. 1998 Jul;53(4):M301-6. doi: 10.1093/gerona/53a.4.m301. PMID: 18314570. *Intervention*

180. Warren C, Medei MK, Wood B, et al. A nurse-driven oral care protocol to reduce hospital-acquired pneumonia: Using evidence-based practice to create a high-priority, high-impact daily intervention. American Journal of Nursing. 2019;119(2):44-51. doi: 10.1097/01.NAJ.0000553204.21342.01. *Duplicate*

181. Weinberger M, Murray MD, Marrero DG, et al. Effectiveness of pharmacist care for patients with reactive airways disease: a randomized controlled trial. JAMA. 2002 Oct 2;288(13):1594-602. doi: 10.1001/jama.288.13.1594. PMID: 12350190. *Intervention*

182. Weisman A, Tompson MC, Okazaki S, et al. Clinicians’ fidelity to a manual-based family treatment as a predictor of the one-year course of bipolar disorder. Family Process. 2002;41:123-31. *Intervention*

183. Weisman AG, Okazaki S, Gregory J, et al. Evaluating therapist competency and adherence to behavioral family management with bipolar patients. Fam Process. 1998 Spring;37(1):107-21. doi: 10.1111/j.1545-5300.1998.00107.x. PMID: 9589285. *Intervention*

184. Welch G, Zagarins SE, Feinberg RG, et al. Motivational interviewing delivered by diabetes educators: does it improve blood glucose control among poorly controlled type 2 diabetes patients? Diabetes Res Clin Pract. 2011 Jan;91(1):54-60. doi: 10.1016/j.diabres.2010.09.036. PMID: 21074887. *Intervention*

185. Wells EA, Calsyn DA, Clark LL, et al. Retention in methadone maintenance is associated with reductions in different HIV risk behaviors for women and men. Am J Drug Alcohol Abuse. 1996 Nov;22(4):509-21. doi: 10.3109/00952999609001677. PMID: 8911589. *Intervention*

186. Wells EA, Clark LL, Calsyn DA, et al. Reporting of HIV risk behaviors by injection drug using heterosexual couples in methadone maintenance. Drug Alcohol Depend. 1994 Aug;36(1):33-8. doi: 10.1016/0376-8716(94)90007-8. PMID: 7988357. *Intervention*

187. Wensing M. The Tailored Implementation in Chronic Diseases (TICD) project: introduction and main findings. Implement Sci. 2017 Jan 10;12(1):5. doi: 10.1186/s13012-016-0536-x. PMID: 28069029. *Intervention*

188. West DS, DiLillo V, Bursac Z, et al. Motivational interviewing improves weight loss in women with type 2 diabetes. Diabetes Care. 2007 May;30(5):1081-7. doi: 10.2337/dc06-1966. PMID: 17337504. *Intervention*

189. Windsor R, Clark J, Cleary S, et al. Effectiveness of the Smoking Cessation and Reduction in Pregnancy Treatment (SCRIPT) dissemination project: a science to prenatal care practice partnership. Matern Child Health J. 2014 Jan;18(1):180-90. doi: 10.1007/s10995-013-1252-7. PMID: 23483412. *Intervention*

190. Wood W, Tschannen D, Trotsky A, et al. A mobility program for an inpatient acute care medical unit: A quality improvement project to mitigate the adverse effects of bed rest shows promise. American Journal of Nursing. 2014;114(10):34-40. *Duplicate*

191. Woolfenden S, Altman L, Breen C. Kids GPS Integrated Care - Leaving the Ivory Tower. International Journal of Integrated Care (IJIC). 2017;17(3):47-8. doi: 10.5334/ijic.3141. PMID: 126048390. Language: English. Entry Date: In Process. Revision Date: 20171108. Publication Type: Abstract. Supplement Title: 2017 Supplement. Journal Subset: Europe. *Setting*

192. Wright WL, Bruns DP, Feeney AS, et al. Improving vaccination rates in older adults: A quality improvement project. Nurse Pract. 2019 Apr;44(4):40-9. doi: 10.1097/01.Npr.0000554085.13073.37. PMID: 30889109. *Intervention*

193. Yamato TP, Maher CG, Saragiotto BT, et al. How completely are physiotherapy interventions described in reports of randomised trials? Physiotherapy. 2016 Jun;102(2):121-6. doi: 10.1016/j.physio.2016.03.001. PMID: 27033780. *Study design*

194. Zabari M, Suresh G, Tomlinson M, et al. Implementation and case-study results of potentially better practices for collaboration between obstetrics and neonatology to achieve improved perinatal outcomes. Pediatrics. 2006;118(SUPPL. 2):S153-S8. doi: 10.1542/peds.2006-0913M. *Duplicate*

195. Zare M, Mejia De Grubb MC, Klawans MR, et al. Multidisciplinary diabetes care in a safety net clinic: Lessons learned from a quality improvement initiative. Journal of Clinical Outcomes Management. 2018;25(5). *Intervention*

196. Zhang NJ, Paek SC, Wan TTH. Reliability estimates of clinical measures between minimum data set and online survey certification and reporting data of us nursing homes. Medical Care. 2009;47(4):492-5. doi: 10.1097/MLR.0b013e31818c014b. *Duplicate*

## Background

Some publications were retained as Background information.

1. Alagoz E, Chih MY, Hitchcock M, et al. The use of external change agents to promote quality improvement and organizational change in healthcare organizations: a systematic review. BMC Health Serv Res. 2018 Jan 25;18(1):42. doi: 10.1186/s12913-018-2856-9. PMID: 29370791. *Background*

2. Alexander KE, Brijnath B, Biezen R, et al. Preventive healthcare for young children: A systematic review of interventions in primary care. Prev Med. 2017 Jun;99:236-50. doi: 10.1016/j.ypmed.2017.02.024. PMID: 28279679. *Background*

3. Blakely CH, Mayer JP, Gottschalk RG, et al. The fidelity-adaptation debate: Implications for the implementation of public sector social programs. American Journal of Community Psychology. 1987;15:253-68. *Background*

4. Calsyn DA, Campbell AN, Crits-Christoph P, et al. Men in methadone maintenance versus psychosocial outpatient treatment: differences in sexual risk behaviors and intervention effectiveness from a multisite HIV prevention intervention trial. J Addict Dis. 2010 Jul;29(3):370-82. doi: 10.1080/10550887.2010.489451. PMID: 20635286. *Background*

5. Crits-Christoph P, Siqueland L, Blaine J, et al. Psychosocial treatments for cocaine dependence: National Institute on Drug Abuse Collaborative Cocaine Treatment Study. Arch Gen Psychiatry. 1999 Jun;56(6):493-502. doi: 10.1001/archpsyc.56.6.493. PMID: 10359461. *Background*

6. Dzidowska M, Lee KSK, Wylie C, et al. A systematic review of approaches to improve practice, detection and treatment of unhealthy alcohol use in primary health care: a role for continuous quality improvement. BMC Fam Pract. 2020 Feb 13;21(1):33. doi: 10.1186/s12875-020-1101-x. PMID: 32054450. *Background*

7. Eames C, Daley D, Hutchings J, et al. The Leader Observation Tool: a process skills treatment fidelity measure for the Incredible Years parenting programme. Child Care Health Dev. 2008 May;34(3):391-400. doi: 10.1111/j.1365-2214.2008.00828.x. PMID: 18410645. *Background*

8. Grandes G, Pinnock H, Bazemore A, et al. Improving the Quality of Primary Care by Optimizing Implementation Research Reporting. J Am Board Fam Med. 2018 May-Jun;31(3):484-7. doi: 10.3122/jabfm.2018.03.170195. PMID: 29743231. *Background*

9. Hankonen N, Sutton S, Prevost AT, et al. Which behavior change techniques are associated with changes in physical activity, diet and body mass index in people with recently diagnosed diabetes? Ann Behav Med. 2015 Feb;49(1):7-17. doi: 10.1007/s12160-014-9624-9. PMID: 24806469. *Background*

10. Hempel S, Newberry S, Wang Z, et al. Hospital fall prevention: a systematic review of implementation, components, adherence, and effectiveness. J Am Geriatr Soc. 2013 Apr;61(4):483-94. doi: 10.1111/jgs.12169. PMID: 23527904. *Background*

11. Hermann RC, Finnerty M, Provost S, et al. Process measures for the assessment and improvement of quality of care for schizophrenia. Schizophr Bull. 2002;28(1):95-104. doi: 10.1093/oxfordjournals.schbul.a006930. PMID: 12047026. *Background*

12. Miake-Lye IM, Hempel S, Ganz DA, et al. Inpatient fall prevention programs as a patient safety strategy: a systematic review. Ann Intern Med. 2013 Mar 5;158(5 Pt 2):390-6. doi: 10.7326/0003-4819-158-5-201303051-00005. PMID: 23460095. *Background*

13. Miller WR, Moyers TB, Ernst D, et al. Manual for the motivational interviewing skill code (MISC). Center on Alcoholism, Substance Abuse and Addictions. Albuquerque, NM: University of New Mexico; 2003. *Background*

14. Moyers TB, Martin T, Manual JK, et al. Revised Global Scales: Motivational Interviewing Treatment Integrity 3.0 (MITI 3.0). In: Mexico UoN, editor Center on Alcoholism, Substance Abuse and Addictions (CASAA). Albuquerque, NM; 2007. *Background*

15. O'Donnell CL. Defining, conceptualizing, and measuring fidelity of implementation and its relationship to outcomes in K–12 curriculum intervention research. Rev Educ Res. 2008;78:33-84. *Background*

16. Pedersen E, Kandrack R, Danz M, et al. Provider Interventions to Increase Uptake of Evidence-Based Treatment for Depression: A Systematic Review. Santa Monica, CA: 2018 2018. https://www.rand.org/pubs/research_reports/RR2111.html. *Background*

17. Pedersen ER, Rubenstein L, Kandrack R, et al. Elusive search for effective provider interventions: a systematic review of provider interventions to increase adherence to evidence-based treatment for depression. Implement Sci. 2018 Jul 20;13(1):99. doi: 10.1186/s13012-018-0788-8. PMID: 30029676. *Background*

18. Roy R, Colquhoun H, Byrne M, et al. Addressing fidelity within complex health behaviour change interventions: A protocol of a scoping review of intervention fidelity frameworks and models. [version 1; peer review: 2 approved]. HRB Open Research. 2018;1(25). doi: 10.12688/hrbopenres.12892.1. *Background*

19. Rubenstein L, Hempel S, Liu JL, et al. The Minimum Quality Criteria Set (QI-MQCS) for Critical Appraisal: Advancing the Science of Quality Improvement. Implementation Science. 2015 August 2015(Suppl. 1):A19. *Background*

20. Rubenstein LV, Hempel S, Farmer MM, et al. Finding order in heterogeneity: types of quality-improvement intervention publications. Qual Saf Health Care. 2008 Dec;17(6):403-8. doi: 10.1136/qshc.2008.028423. PMID: 19064654. *Background*

21. Soban LM, Hempel S, Munjas BA, et al. Preventing pressure ulcers in hospitals: A systematic review of nurse-focused quality improvement interventions. Jt Comm J Qual Patient Saf. 2011 Jun;37(6):245-52. PMID: 21706984. *Background*

22. Terens N, Vecchi S, Bargagli AM, et al. Quality improvement strategies at primary care level to reduce inequalities in diabetes care: an equity-oriented systematic review. BMC Endocr Disord. 2018 May 29;18(1):31. doi: 10.1186/s12902-018-0260-4. PMID: 29843692. *Background*

23. Walker MF, Hoffmann TC, Brady MC, et al. Improving the Development, Monitoring and Reporting of Stroke Rehabilitation Research: Consensus-Based Core Recommendations from the Stroke Recovery and Rehabilitation Roundtable. Neurorehabil Neural Repair. 2017 Oct-Nov;31(10-11):877-84. doi: 10.1177/1545968317732686. PMID: 29233072. *Background*
